# Repetitive DNA content in the maize genome is uncoupled from population stratification at SNP loci

**DOI:** 10.1186/s12864-020-6517-0

**Published:** 2020-01-30

**Authors:** Simon Renny-Byfield, Andy Baumgarten

**Affiliations:** Corteva Agriscience, 62nd Ave, Johnston, USA

**Keywords:** Repetitive DNA, Population structure, Plant breeding

## Abstract

**Background:**

Repetitive DNA is a major component of plant genomes and is thought to be a driver of evolutionary novelty. Describing variation in repeat content among individuals and between populations is key to elucidating the evolutionary significance of repetitive DNA. However, the cost of producing references genomes has limited large-scale intraspecific comparisons to a handful of model organisms where multiple reference genomes are available.

**Results:**

We examine repeat content variation in the genomes of 94 elite inbred maize lines using graph-based repeat clustering, a reference-free and rapid assay of repeat content. We examine population structure using genome-wide repeat profiles, and demonstrate the stiff-stalk and non-stiff-stalk heterotic populations are homogenous with regard to global repeat content. In contrast, and similar to previously reported results, the same individuals show clear differentiation, and aggregate into two populations when examining population structure using genome-wide SNPs. Additionally, we develop a novel kmer based technique to examine the chromosomal distribution of repeat clusters in silico and show a cluster dependent association with gene density.

**Conclusion:**

Our results indicate global repeat content variation in the heterotic populations of maize has not diverged, and is uncoupled from population stratification at SNP loci. We show that repeat families exhibit divergent patterns with regard to chromosomal distribution, some repeat clusters accumulate in regions of high gene density, whereas others aggregate in regions of low gene density.

## Background

Maize is a commercially important hybrid crop grown globally for livestock consumption, food, and fuel. Early maize breeding programs observed that crosses between maize populations resulted in increased heterosis where F1 progeny or hybrids demonstrate significantly higher performance than parental lines [1, 2]. Public and private industry breeding programs have exploited heterosis in maize to maximize genetic gain for grain yield [3]. These programs employ reciprocal recurrent selection to develop specific germplasm pools, called heterotic groups, that maximize heterosis. Improvement occurs with the development of inbred lines that maximize heterosis in crosses between heterotic groups. Inbred lines are then recycled to create new crosses within each heterotic pool.

Several studies have demonstrated that reciprocal recurrent selection drives differences in allele frequencies between heterotic groups. These studies have coupled genotyping and population genetics to demonstrate there are three distinct heterotic pools within current North American elite maize germplasm. These pools, referred to as Stiff-Stalk (SS), Non-Stiff-Stalk (NSS), and Iodent (IOD), show increasing allele frequency divergence over time with recently developed lines having the greatest divergence [4].

Previous studies examining the genetic divergence, allelic frequency, and linkage disequilibria between maize heterotic pools have focused solely on SNPs found in genic regions of the maize genome. However, the maize genome consists primarily of various repetitive DNA families, similar to other species with similar genome size. Studies have demonstrated that repetitive DNA dynamics can influence gene content changes [5], changes in genome size [6], and gene expression activation within plants [7, 8]. A study comparing the divergence and abundance of repeat variation between maize heterotic pools could provide insight to whether repeats follow the same divergence as SNP-based diversity studies.

Unfortunately, the cost and complexity of producing high-quality reference genomes limits reference-based comparisons of repeat content to a few species and individuals with fully sequenced genomes (although the cost is rapidly declining). These cost and analysis restrictions limit the ability to compare repeat content and its association to population and phenotypic variation. However, reference-free approaches using low coverage short-read sequencing and graph-based repeat clustering have been used to efficiently assay repeat content in eukaryotic genomes [9]. Reference-free, skim-sequencing methods exploit repeat sequence abundance to estimate genome composition across hundreds of genomes without the use of a genomic reference [10–14]. The highly repetitive nature of the maize genome ensures the majority of genomic short-read sequences will be repetitive in nature.

Reference-free approaches, using skim sequencing and repeat clustering, were successfully used to assay repeat content in allopolyploid tobacco and demonstrated the biased removal of repetitive DNA from one of the two sub-genomes [13]. Furthermore, similar clustering, along with empirical wet-lab validation, was used to demonstrate the rapid elimination of tandem repeat sequences in synthetic allopolyploids [10]. Other studies in *Nicotiana* have shown reductions in genome size occur via the loss of low copy-number repeats while genome expansion results from the increase of already highly abundant repeats [6]. Additionally, repeat clustering revealed repeat content of rye B-chromosomes [15] and the giant genomes of *Fritilaria* [11]. More importantly, repeat profiles generated by repeat-clustering exhibit phylogenetic signal consistent with that observed for plastid and nuclear markers in a variety of taxa [16], suggesting signals from skim sequencing assays can track long-term evolutionary trajectories.

In this study, we take advantage of skim sequencing and graph-based repeat clustering to screen the genomes of 94 elite ex-PVP maize inbreds. Ex-PVP lines are inbreds developed and patented by private or public institutions but have had their patents expire allowing public breeding and genotyping use. Many of the lines included in this study represent the base germplasm used to establish current industry and public North American maize breeding programs [17]. We identify and annotate repeat families de novo and examine their differential abundance within and between heterotic groups. The chromosomal distribution of repeat families was also compared between several high-quality reference maize genomes using a novel k-mer based method.

Our analysis revealed no significant differentiation in abundance of the two major classes of repeats present in the maize genome, *Gypsy* and *Copia* LTR-retroelements, when comparing two maize heterotic populations. We observed that chromosomal distribution varies between repeat families and statistically significant association exists between the chromosome distribution of repeat clusters and gene density. Using SNP data we demonstrate significant population structure between the two maize heterotic pools, in contrast, similar population structure was not found using repeat clustering data. This suggests divergence of the heterotic groups has occurred at the SNP level, but this process is not mirrored at the level of repeat abundance or genomic distribution.

## Methods

### Plant material, data preparation and read sampling

Ninety four *Zea mays* ssp. *mays* ex-PVP lines were used in this study (see Additional file 1). The provenance, collection date, collector, voucher certification, and seed for all samples are publicly available at the U.S. National Plant Germplasm System (https://www.ars-grin.gov/pvp).

The material used was categorized into stiff-stalk (SS) and non-stiff-stalk (NSS + IOD) heterotic groups. DNA was extracted with V5 stage leaf tissue from greenhouse grown accession using the CTAB method. Illumina HiSeq 2000 libraries where prepared from purified DNA according to the manufacturers instructions and samples were sequenced at 20–60x coverage using the Illumina HiSeq 2000 instrumentation resulting in 150 bp paired-end reads. For each sample, the resulting sequencing data were randomly down-sampled, resulting in a skim sequence dataset of 50,000 whole genome shotgun (WGS) reads per individual, taking only one read from a pair. Sequence reads are available at the NCBI SRA under the BioProject number PRJNA530574.

### Graph-based repeat clustering

We implemented graph-based clustering, based on previously published methods [9], using data from all sequenced ex-PVP inbreds as input. The method from [9] was re-written in Python 2.7 using the Python version of igraph [18] to increase analysis speed. Graph-based repeat clusters was performed by pooling sequencing reads from all ex-PVP inbreds into a single dataset. Sequence reads from each individual were tracked in the combined pool to allow each read to be linked to the individual inbred it came from. Repeat families were then identified using a graph-based clustering approach similar to previously described methods [9].

Briefly, a complete pair-wise comparison is performed between all reads using megablast [19]. Using this data a simple, undirected graph of the form

1 $$ G=(v,e)  $$

was constructed from the resulting pair-wise relationships such that vertices (*v*) of the graph (*G*) represent reads from our dataset and edges (*e*) between the vertices correspond to megablast hits between the reads. Edges were weighted according to the bit-score of the corresponding blast hit and edges with bit-score less than 100 were excluded from further analysis. The graph object was reduced to its largest connected component and a community detection process was performed using the fast_greedy algorithm [20] to detect repeat families. This process of community detection established clusters of reads closely connected in the graph representing highly-related repetitive DNA families. The number of reads contributed from the sequence of each ex-PVP inbred was quantified for each cluster. For the 25 largest clusters we extracted the cluster sub-graph *G*_*s*_ from the graph *G* and derived the sub-graph layout using the Fruchterman and Reingold algorithm [21], positioning vertices (reads) such that reads containing similar sequence are placed close together in 2D space.

We generated a cluster graph (*G*_*c*_) from the graph *G* of the form:

2 $$ G_{c}=(v,e)  $$

where vertex *v*_*i*_ represents the *i*th cluster, and the edge *e*(*v*_*i*_,*v*_*j*_) represent the sum edge weight of all edges between *v*_*i*_ and *v*_*j*_ in the graph *G*. This graph describes the relationships and connections between clusters, where each cluster has been reduced to a single vertex.

### Repeat cluster annotation

Each cluster was annotated by extracting all sequence reads and using RepeatMasker [22], with the cross_match option, to search against the *plant collections* of the RepBase database [23]. Each cluster was then defined by the most common repeat match. We counted all the reads attributed to each cluster, and each annotation, on a per individual basis. We examined for statistical differences in mean cluster and mean annotation abundance in the SS and NSS population using a two-sided t-test, correcting for multiple comparisons using the method of Benjamini and Hockberg [24].

### K-mer analysis and chromosome painting

For each of the 25 largest clusters we identified the 100 most abundant k-mers (of length 12) using JellyFish [25]. From each of the largest 25 clusters the 100 most abundant k-mers were mapped to several representative reference genomes using the *fuzznuc* function of the EMBOSS package [26]. These genomes were B73 RefGen_v4 (SS founder, downloaded https://www.maizegdb.org/ accessed 19/9/2017), B73 RefGen_v3.23 (SS founder, downloaded https://www.maizegdb.org/ accessed 19/9/2017), PH207 (Iodent, downloaded https://www.maizegdb.org/ accessed 19/9/2017), Mo17 (NSS founder, downloaded https://www.maizegdb.org/ accessed 1/1/2020), and CML247 (tropical line, downloaded https://www.maizegdb.org/ accessed 1/1/2020) reference genomes. We then counted the number of k-mers mapping to 200 kb windows of each genome (step-size 100 kb) using bedtools, scaled the data and loess smoothed over profiles using the scipy package in python 2.7. For each reference sequence we generated gene density traces (where annotations were available) using the *coverage* tool of the *bedtools* package over the same 200 kb windows of the genome. Using scaled data for each of the 25 largest clusters, we calculated the Pearson’s and Spearman’s correlation coefficient between gene density and kmer mapping density using the scipy package in python 2.7.

### PCA analysis

Using cluster abundance estimates from each of the 94 inbreds, we performed PCA analysis. Abundance measures were normalized on a cluster by cluster basis using minimum/maximum scaling of the statistics package sklearn in python 2.7, such that values for each cluster ranged from zero to one. Using the normalized data we fit a PCA with 2 components using functionality of the sklearn package in Python 2.7. We performed the same analysis, but in place of cluster abundances used 1585 genome-wide SNP markers, encoding the major allele as 1, the minor allele as 0, and masking heterozygous sites.

## Results

### Graph-based repeat clustering

Graph-based repeat clustering methods were used to analyze low coverage sequence data from 94 elite, ex-PVP maize inbreds. The resulting graph (*G*) contained 2,956,096 vertices (reads) and 244,031,339 edges (sequence similarity hits between reads), with a mean degree of 165.10. Community detection using the fast_greedy algorithm grouped vertices into 728 repeat clusters with the largest cluster containing 207,094 vertices and the smallest containing two.

**Fig. 1:**
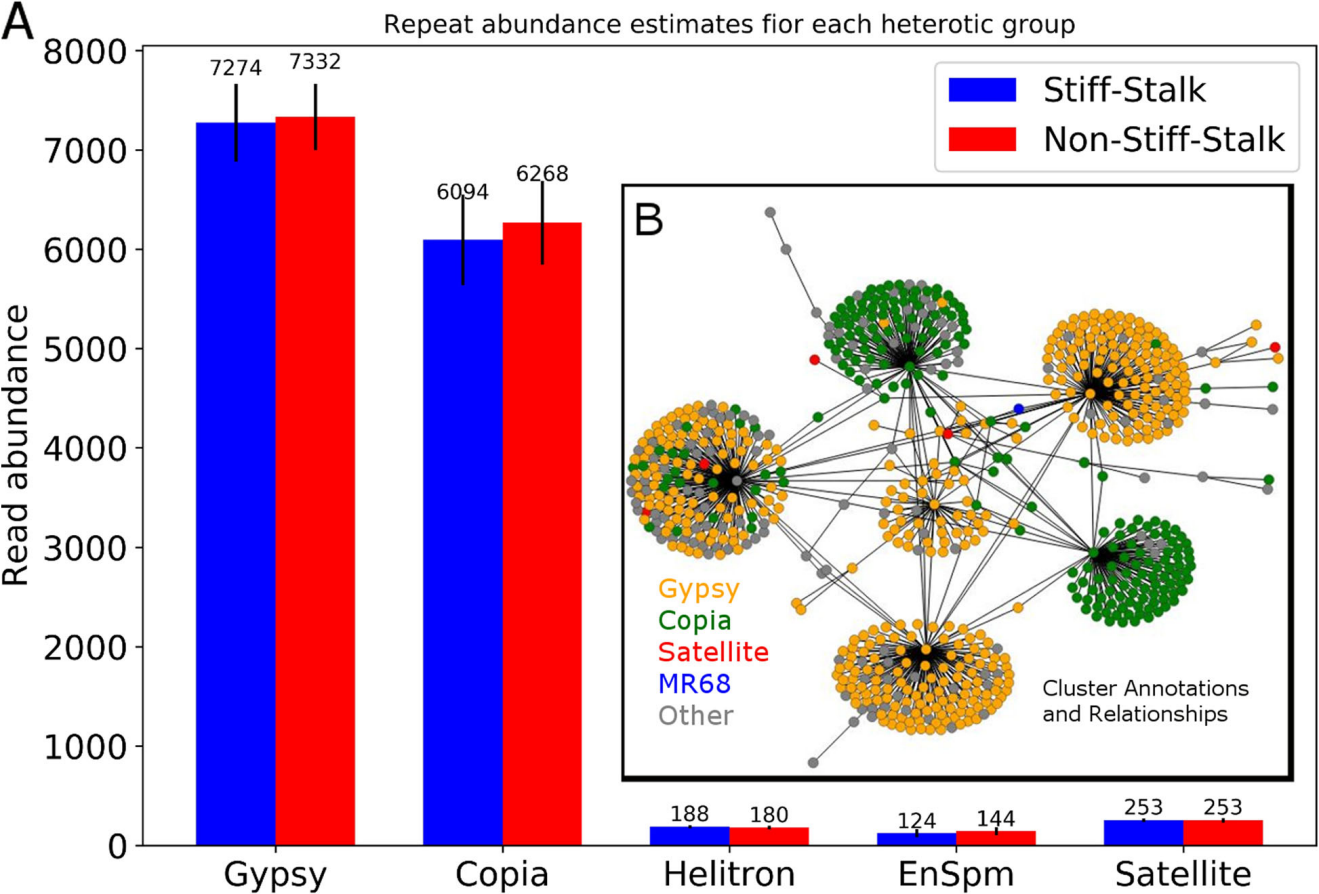
Repeat family abundance estimates and cluster relationships. **a** Using the annotation associated with each cluster we summed the total number of reads from each inbred attributed to a given annotation type, and display the mean (bars) and standard deviation (whiskers) for the SS and NSS populations for select repeat annotations. In **b** is displayed the cluster graph *G*_*c*_, where nodes are clusters and the edges indicate cross-cluster megablast hits, nodes are coloured according to annotation type

Annotation of the clusters using the Repbase repeat library revealed a diverse collection of repeat families with the majority of repeats families categorized as either *Gypsy* or *Copia* LTR retroelements (Fig. 1a and Additional files 2 and 3). A higher abundance of *Gypsy*-like elements was observed, when compared with *Copia*-like elements. This was true for both the overall dataset (NSS and SS populations combined; t-statistic = 21.23, *p*-value <0.0001), and when considering SS (t-statistic = 14.14, *p*-value <0.0001) and NSS (t-statistic = 15.83, *p*-value <0.0001) populations separately. No significant difference was seen when comparing the abundance of *Gypsy*-like repeat families between SS and NSS inbreds (t-statistic = 15.83, *p*-value= 0.38). However, marginally significant differences were observed in the total abundance of *Copia*-like repeats between SS and NSS inbreds (t-statistic = -2.12, *p*-value = 0.045).

We tested for significant differences in the abundance of individual repeat clusters between the NSS and SS populations. After correcting for multiple comparisons, we found that only three of the 728 clusters exhibited evidence of differential abundance between SS and NSS inbreds (*p*-value <0.05).

Generating a meta-graph, where each individual cluster is collapsed to a single vertex and connections between clusters is indicated by edges, we see that there are dense and numerous connections between repeats of the same type (i.e. within and between *Gypsy*-like clusters, or within and between *Copia*-like clusters), and rather fewer between clusters belonging to alternate families (Fig. 1b).

### Chromosomal distribution and correlation with gene density

**Fig. 2:**
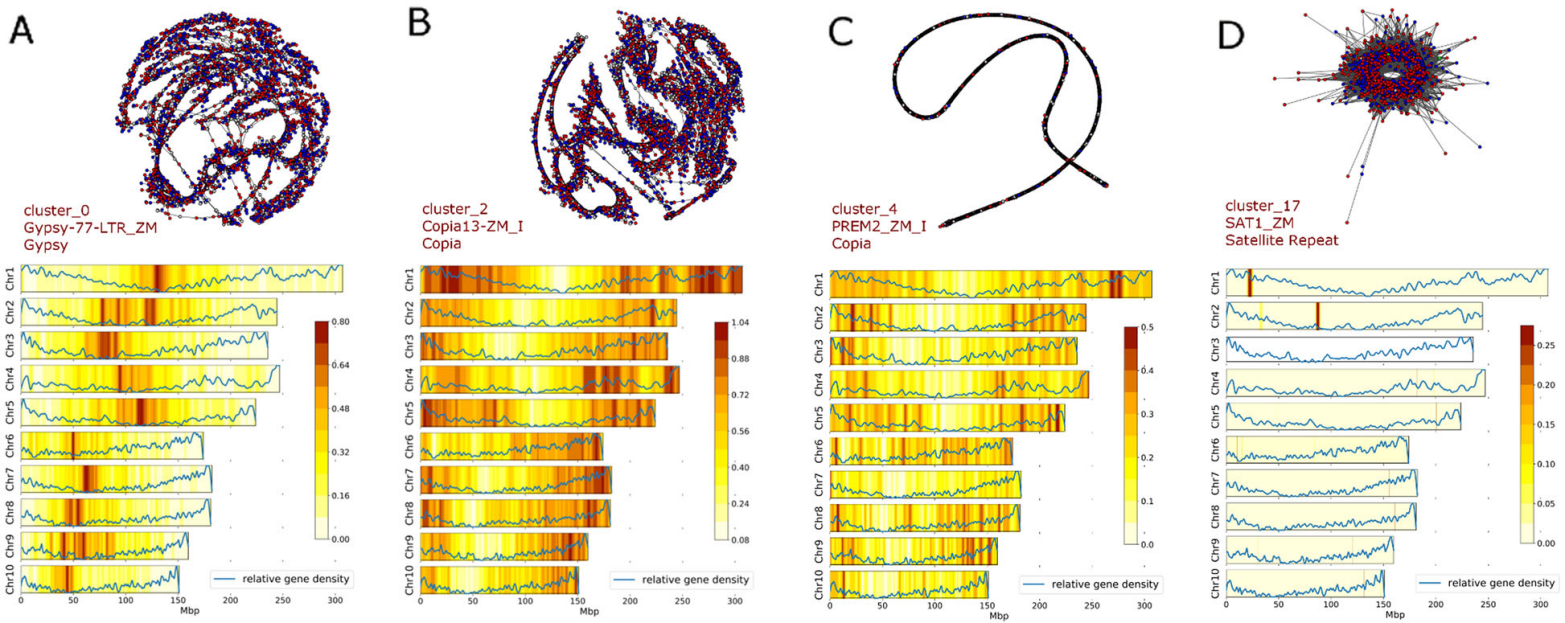
Repeat Cluster layouts and Chromosomal Distributions. Graph layouts and chromosomal distributions are displayed for clusters 0, 2, 4 and 17 (**a**–**d** respectively). Cluster layouts, in 2D space, are calculated using the Fruchterman-Reingold algorithm and are shown in the top of each panel. Reads deriving from Stiff-stalk (SS) and Non-stiff-stalk (NSS) inbreds are indicated by blue and red vertices respectively. Edges, indicated in gray, represent sequence similarity hits between reads (vertices). The cluster name, most common hit to RepBase and the repeat family to which each cluster belongs is indicated. The most abundant k-mers from each repeat cluster were mapped to the maize B73 RefGen_v3 genome sequence, and the resulting loess smoothed profiles for each chromosome are displayed in the lower portion of each panel. Gene density profiles are also shown in blue, overlaying the repeat profiles

For each of the 25 most abundant clusters we calculated the cluster layout and placed vertices (reads) in 2D space. This analysis revealed incredible diversity in cluster layout (Fig. 2a-d) within the maize genome. Tightly compressed clusters represent collections of highly similar reads since each vertex is positioned based on the edge weight of connections to other vertices.

**Fig. 3:**
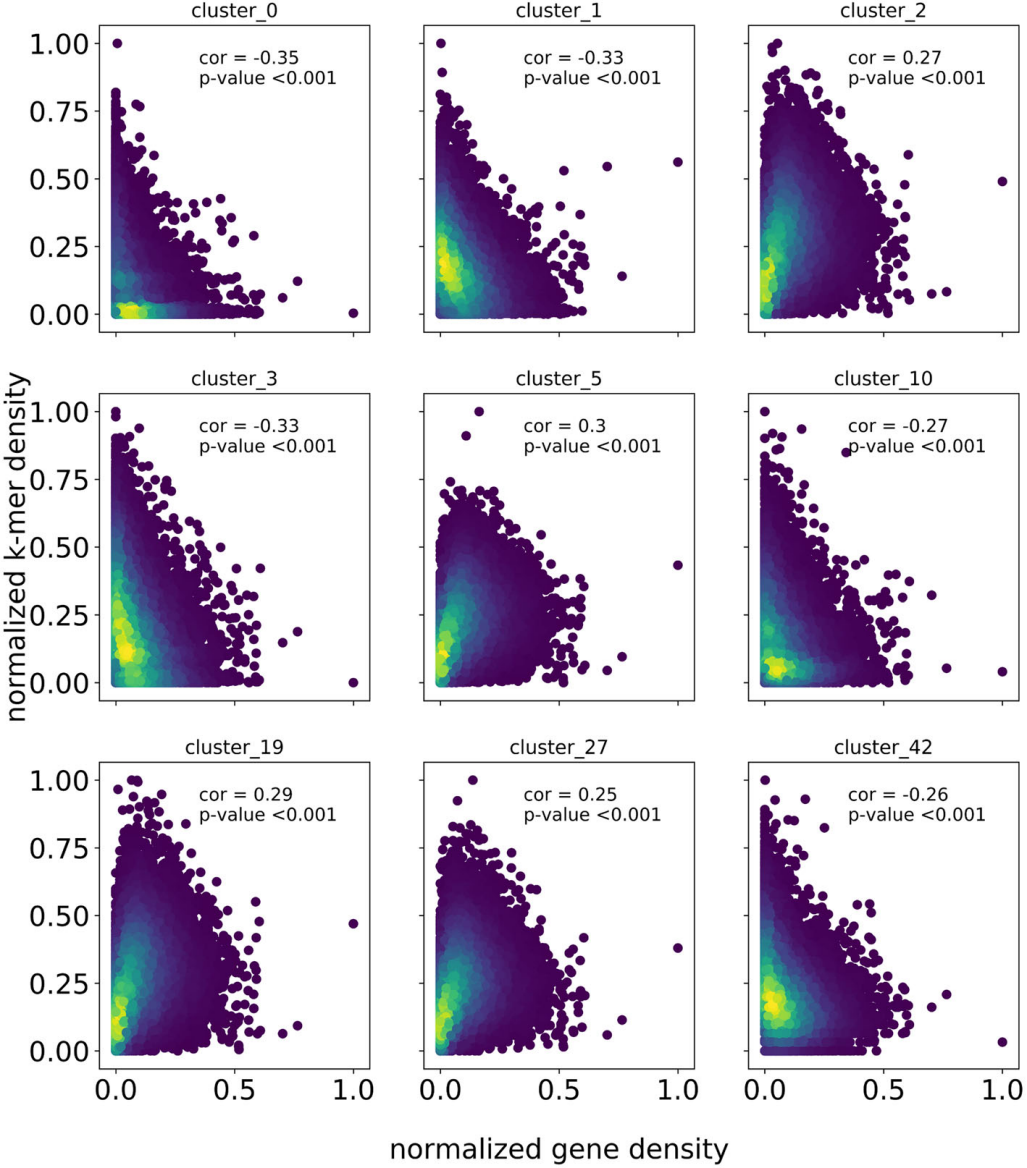
Scatter plots showing the relationship between gene density and kmer mapping density in 200 kb windows of the genome for examplar repeat clusters. For each example the Pearson’s correlation coefficient and associated *p*-value are given. For cluster 0, 1, 3, 10 and 42 there is a significant negative correlation, whereas a positive correlation is observed for clusters 2, 5, 19 and 27. Each data point is colored by a two dimensional kernel density estimation, where the purple to yellow color indicates ever greater density of data points

We deploy a novel in silico chromosome painting technique to investigate the chromosomal distribution of individual clusters. We identified the 100 most common k-mer sequences within each cluster and mapped these to the genome to establish the chromosomal distribution of each cluster (Fig. 2 and Additional files 4, 5, 6, 7, and 8). Repeats from several clusters preferentially mapped to gene poor pericentromeric regions (Fig. 2a) whereas k-mers from other clusters map to gene rich regions of the genome, and have lower abundance in gene poor centromeric regions (Fig. 2b). We quantified this trend by determining the correlation between kmer mapping density and the proportion of genic sequence in the chromosomal region of interest (Fig. 3). Forty four of the 50 largest repeat clusters showed statistically significant (*p*-value <0.05) association with gene density.

In addition, we mapped k-mers to various maize reference assemblies to determine if we could identify differential cluster distribution between individual lines (Additional files 4, 5, 6, 7, and 8). The analysis revealed striking similarities between the representative genomes; although clusters did display some micro-variation across chromosomes most clusters shared very similar chromosomal distributions across lines and heterotic groups.

### Population structure: a tale of two datasets

**Fig. 4:**
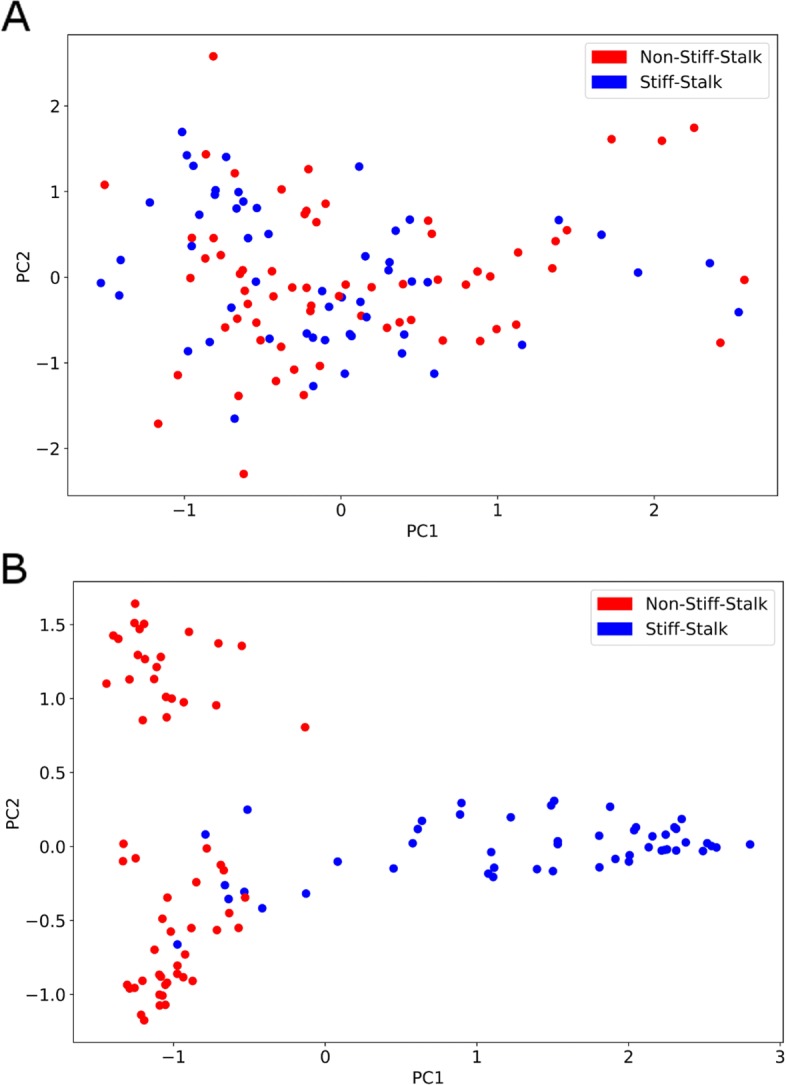
Population structure in the two heterotic groups of maize. In (**a**) the first two dimensions of a PCA analysis are used to describe the population structure as inferred by repeat content of the genome (i.e. abundance estimates of the clusters). In (**b**) PCA is used to infer population structure, but using genome wide SNP markers

We examined population structure among 94 ex-PVP inbred maize lines using global repeat abundance profiles and separately using standard SNP genotypes (Fig. 4). A PCA analysis using repeat abundance estimates revealed that ex-PVP inbreds do not appear to cluster into known heterotic groups (Fig. 4a). The SS and NSS individuals form a single homogeneous group with SS and NSS individuals intermixed. In contrast population structure, as estimated by a traditional approach using genotype data and PCA analysis, reveals that the SS and NSS heterotic pools are distinct, with almost no overlap between the NSS and SS groups (Fig. 4b).

## Discussion

### Global repeat abundance profiles

Graph-based repeat clustering is a computational approach that identifies and quantifies repeat families within a genome without the need for a reference genome. The approach utilizes low-coverage sequence data to construct a graph of relationships between reads based on sequence similarity. This graph was analyzed using community detection approaches to group similar reads into clusters, each cluster representing a different repeat family. These methods were used to analyze low coverage sequence from 94 elite, ex-PVP maize inbreds.

Annotation of the clusters revealed that the majority of the repeat content of NSS and SS genomes is comporised of *Gypsy* and *Copia* LTR retroelements (See Additional files 2 and 3). These results are similar to, and expected, given previous studies using reference quality genome assemblies in maize [27, 28]. A higher abundance of *Gypsy*-like elements was observed, when compared with *Copia*-like elements, across the population as a whole, as well as within each heterotic sub-population.

We tested for significant differences in the abundance of individual repeat clusters between the NSS and SS populations. After correcting for multiple comparisons, we found that only three of the 728 clusters exhibited evidence of differential abundance between SS and NSS inbreds. This observation suggests that the NSS and SS populations are very similar in terms of global repeat abundance profiles, although differences between individuals exist. It should be stressed that the similarities in repeat abundance estimated by graph-based clustering are only representative of global repeat content in the genome. In contrast, local repeat and TE variation in and around genes is incredibly diverse between maize inbreds [29]. Such micro-scale variation is not reflected in our global repeat profiles.

The observation of little to no differentiation between maize heterotic groups is in contrast to other comparisons using the same methodology. For example, graph-based repeat clustering is able to detect changes in repeat abundance between closely related species in a number of plant families [6, 12, 14]. Other studies have identified and confirmed differential repeat content in rye accessions segregating for B-chromosomes [15]. Similarly, clustering analysis and empirical validation tracked the fate of individual repeat families in early generation synthetic allopolyploid tobacco and demonstrated alteration in the chromosomal distribution and abundance of those clusters [10]. As such, we know graph-based clustering is a suitable method to quickly assay repeat content data, and detect differences between individuals of the same species or closely related taxa.

### Chromosomal distribution and correlation with gene density

For the largest clusters we calculated a cluster layout, placing vertices (reads) and edges in 2D space (Fig. 2a-d). The layout of each cluster can reveal interesting biological properties. For example sequence uniformity in reads from a repeat cluster may indicate a recent copy-number expansion, and this is indicated in the tight-knit layout of vertices (Fig. 2c). In contrast, clusters with a more dispersed layout (Fig. 2a,b) contain more divergent reads and likely represent older populations of repeats, where extensive sequence divergence has accumulated between members of the family. Several clusters, including cluster_17 (Fig. 2d), exhibit a ring like structure, a layout resulting in repeating units arranged in tandem, a hallmark of tandem repeat sequences [10]. Importantly, such clusters typically exhibit highly localized chromosomal distributions, as might be expected of a tandem repeat sequence (Fig. 2d, bottom panel).

Using k-mer mapping we established the chromosomal distribution of several of the largest repeat clusters revealing substantial variation in chromosomal distribution, depending on the cluster (Fig. 2). We also demonstrate that 44 of the 50 largest clusters have statistically significant association with gene density (*p*-value <0.05). Using cluster_0 as an example the density of mapped kmers is significantly negatively correlated with gene density, suggesting this repeat family preferentially insert into gene poor regions of the genome, or alternatively that copies are removed more easily from genic regions (Figs. 2 and 3). Interestingly we see the opposite pattern for kmers derived from cluster 2, where a positive association is observed between kmer mapping density and gene density suggesting this repeat family aggregates in relatively gene rich regions. It also worth noting that, while there is a positive relationship between gene density and kmer mapping density for some clusters, we tend not to see the accumulation of kmer mapping events in the most gene dense regions of the genome (Fig. 3).

The observation that chromosomal distribution varies between clusters and is associated with gene density is similar to previous studies on repeat sequences in maize [30, 31] and other plant species [32–34]. For example *Ds* elements have accumulated in the sub-telomeric regions of maize chromosomes, but are relativity rare in pericentromeric regions [30]. *Gypsy*, CACTA, and *Copia* elements have varying chromosomal distribution in the *Setaria* genome [35]. More broadly, these observation likely reflect differences in the insertion preferences between repeat families and bias in the removal of repeat copies related to gene density.

Using kmer painting we investigated the chromosomal distribution of clusters in different maize inbred lines (Additional files 4, 5, 6, 7, and 8). The data demonstrate that individual clusters share remarkably similar distributions across individuals, and indeed, across heterotic groups; broadly speaking, the distribution of a given clusters is very similar, regardless of the individual line in question. This may indicate the global patterns of repeat distribution were laid down before the divergence of maize founder lines. Furthermore, it is possible that, as repeat families have waxed and waned since the founding of elite maize heterotic groups, the global distribution of repeats has remained relatively unchanged.

### Population structure: a tale of two datasets

Examining population structure using SNP genotypes reveals the two heterotic groups, SS and NSS are divergent and easily distinguished (Fig. 4). However, when we examine population structure as measured by repeat abundance profiles we see that the two heterotic pools are intermixed. This suggests that the SS and NSS populations are not distinct with in regard to patterns of global repeat abundance, even though repeat differences exist between individuals. In contrast, a similar PCA analysis performed using SNP information from the same inbreds separated known SS and NSS inbreds into distinguishable groups, especially on the first principle component (Fig. 4b). The results of the analysis using SNPs are highly similar to that observed in previous studies using the elite breeding lines and historical material [4, 36].

The population structure observed in maize ex-PVP germplasm is expected and is reflective of the reciprocal recurrent selection breeding methods used to drive heterosis between heterotic pools [36]. This trend is not observed when using global repeat abundance profiles, nor when considering chromosomal distributions of repeat families (Additional files 4, 5, 6, 7, and 8). This suggests that allele frequency alterations, caused by selection or drift, do not result in a corresponding divergence in global repeat abundance. One possible explanation is that segregating haplotypes often contain similar repeat content but vary in SNP content. In this case selection for beneficial SNP alleles could change SNP frequencies within the population but have limited effect on global repeat profiles. This observation could be compounded because current elite maize germplasm pools were developed from a collection of similar landrace founders [36]. Potentially, global repeat content was rather uniform among founders and has not yet diverged in modern breeding populations.

Our result contradicts the observation that inbred maize genomes show high variability in repeat co-linearity in and around genes [29] and across the genome [37]. One might imagine that this variation should track with linked SNPs and over time become evident in global repeat profiles. However, the difference in signal from SNP and repeat abundance profiles could be driven by the ability of repeats, particularly transposable elements, to readily break linkage associations (via transposition), limiting the effect of breeding history on their distribution and abundance. Lastly, the possibility remains that graph-based clustering and quantification is not adequate to detect the variation we know exists. As mentioned previously, this seems unlikely given the wealth of previous studies where variation in repeat content has been readily detected [6, 10, 12, 15], and indeed, can follow close relationships between taxa [16].

## Conclusion

We demonstrate the utility of graph-based clustering for repeat identification and quantification in maize, and develop a kmer-based approach to analyzing repeat content distribution across chromosomes in silico. Kmer painting of repeats reveals various chromosomal distribution patterns and we provide evidence that repeat clusters accumulate along chromosomes in a cluster dependent manner that can be negatively, or positively associated with local gene density. This novel analysis provides an additional avenue of investigation for researchers using graph-based repeat identification.

Additionally, we reveal that genetic population structure, as indicated by genotype data, distinguishes two well-known heterotic groups on elite maize, whereas global repeat populations are homogeneous between the two populations. These observations suggest that the highly dynamic repeat fraction of the inbred maize genome has not diverged in concert with SNP profiles in the two populations, SNP and repeat abundance profiles are uncoupled.

## Supplementary information

**Additional file 1** List of ex-PVP *Zea mays* spp *mays* inbreds used in this study. A list of the public names for ex-PVP inbreds used in this study, as well as their SRA sample names.

**Additional file 2** Cluster annotation information. File listing clusters and their associated annotation as determined by sequence matches to RepBase

**Additional file 3** Cluster read counts by inbred. A file listing cluster name and the number of reads from each inbred that contributed to the given cluster.

**Additional file 4** Genome-wide kmer distribution in b73 v3.23. The genome-wide K-mer distribution for each of the 25 largest repeat clusters. A Kernel density estimation is indicated by color, and associated gene density estimates are also given.

**Additional file 5** Genome-wide kmer distribution in b73 v4.0. The genome-wide K-mer distribution for each of the 25 largest repeat clusters. A Kernel density estimation is indicated by color, and associated gene density estimates are also given.

**Additional file 6** Genome-wide kmer distribution in pH207. The genome-wide K-mer distribution for each of the 25 largest repeat clusters. A Kernel density estimation is indicated by color, and associated gene density estimates are also given.

**Additional file 7** Genome-wide kmer distribution in mo17. The genome-wide K-mer distribution for each of the 25 largest repeat clusters. A Kernel density estimation is indicated by color, and associated gene density estimates are also given.

**Additional file 8** Genome-wide kmer distribution in CML247. The genome-wide K-mer distribution for each of the 25 largest repeat clusters. A Kernel density estimation is indicated by color, and associated gene density estimates are also given.

## Abbreviations

ex-PVP: ex Plant Variety Protection
IOD: Iodent
NSS: Non-Stiff-Stalk
PCA: Principal Component Analysis
SS: Stiff-Stalk
