# Supplementary material for: Repetitive DNA content in the maize genome is uncoupled from population stratification at SNP loci
Source: BMC Genomics. 2020 Jan 30;21:98. doi: 10.1186/s12864-020-6517-0 (PMC6993463; doi:10.1186/s12864-020-6517-0)
Supplement: Supplementary file 6 — Additional file 6 Genome-wide kmer distribution in pH207. The genome-wide K-mer distribution for each of the 25 largest repeat clusters. A Kernel density estimation is indicated by color, and associated gene density estimates are also given. [file 12864_2020_6517_MOESM6_ESM.pdf]

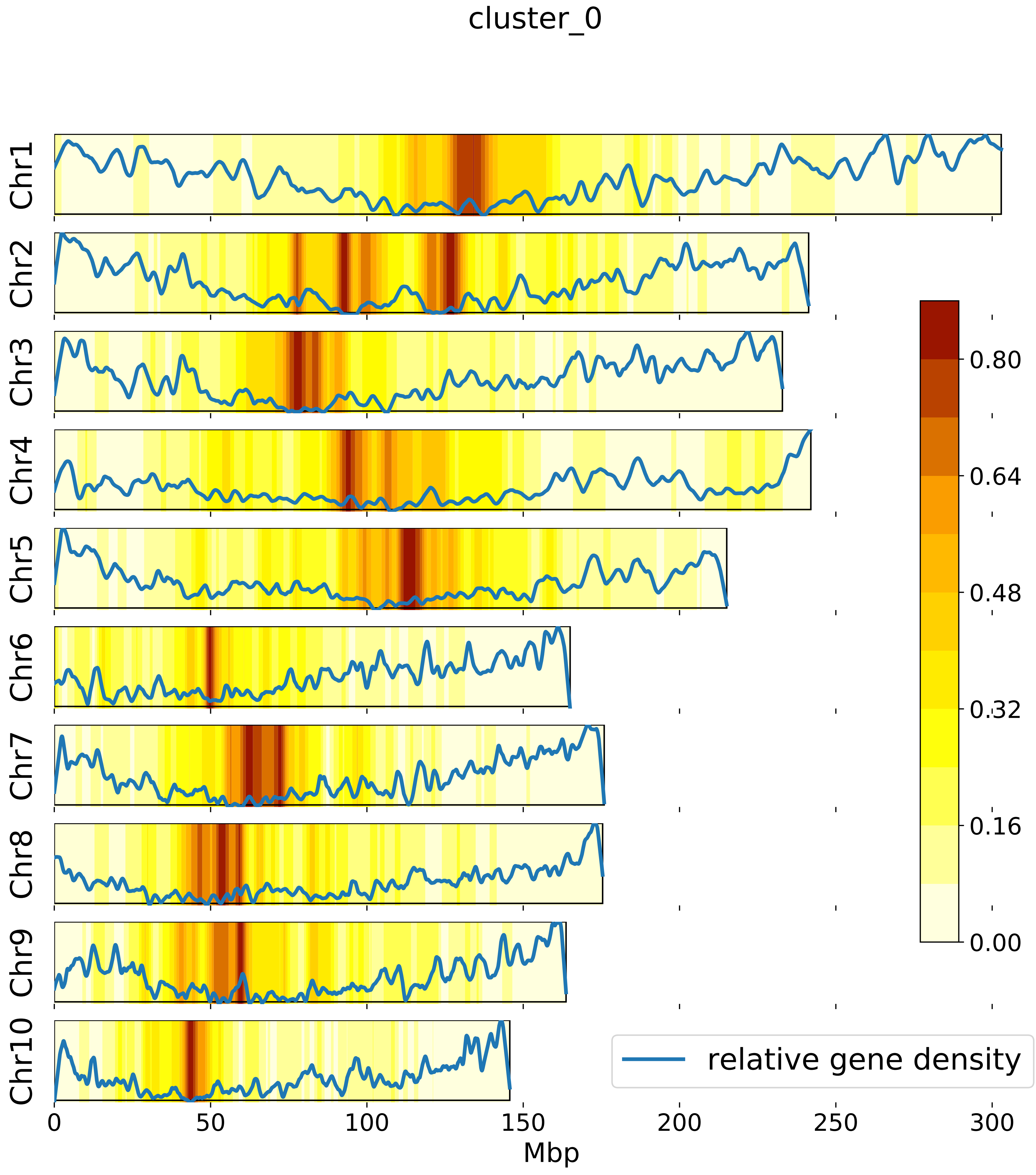
cluster_0
Chr1
Chr2
Chr3
Chr4
Chr5
Chr6
Chr7
Chr8
Chr9
Chr10
0
50
100
150
200
250
300
Mbp
0.80
0.64
0.48
0.32
0.16
0.00
relative gene density

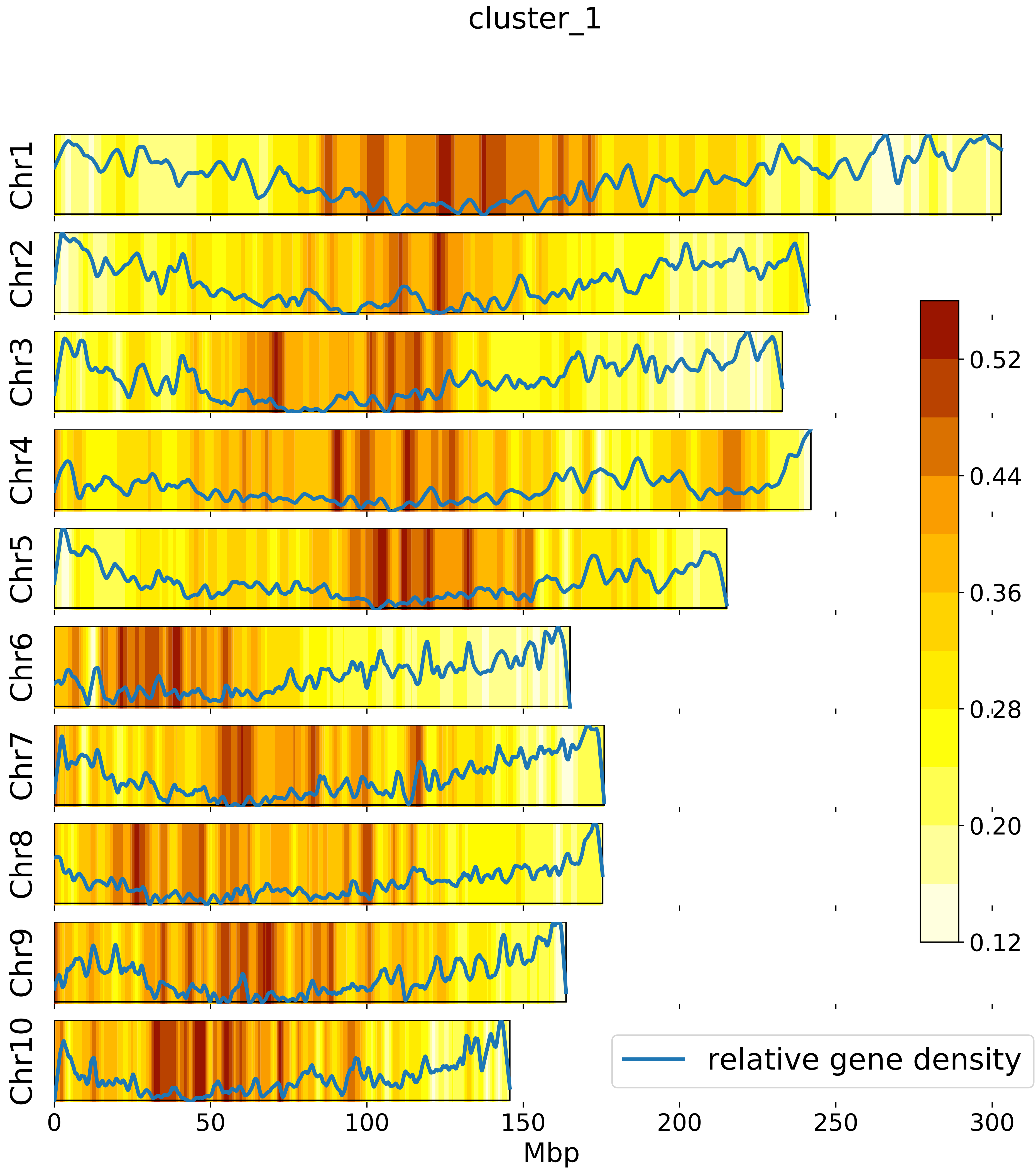

cluster_1
Chr1
Chr2
Chr3
Chr4
Chr5
Chr6
Chr7
Chr8
Chr9
Chr10
0.52
0.44
0.36
0.28
0.20
0.12
relative gene density
0
50
100
150
200
250
300
Mbp

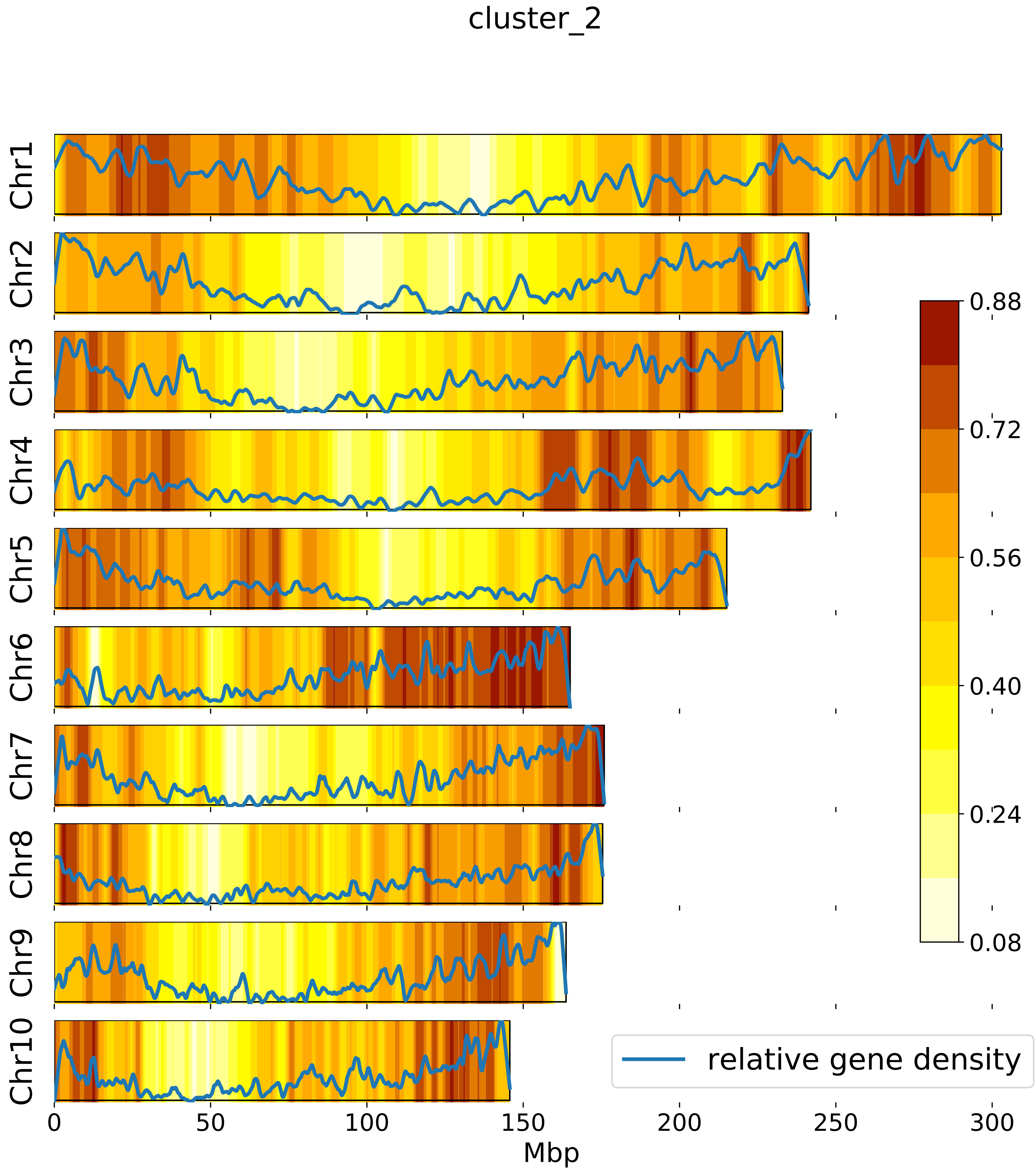

cluster_2
Chr1
Chr2
Chr3
Chr4
Chr5
Chr6
Chr7
Chr8
Chr9
Chr10
0
50
100
150
200
250
300
Mbp
0.88
0.72
0.56
0.40
0.24
0.08
relative gene density

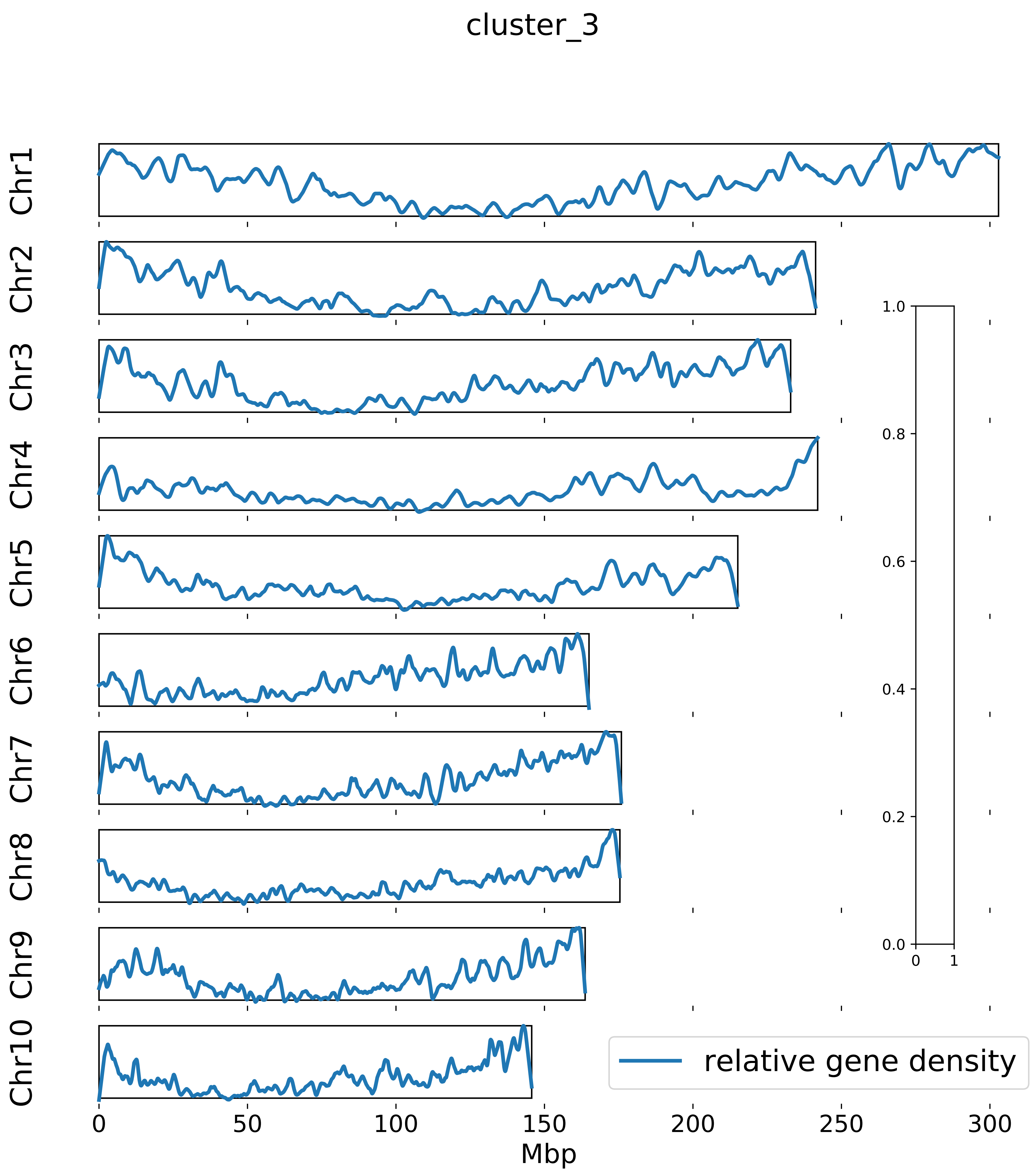

cluster_3
Chr1
Chr2
Chr3
Chr4
Chr5
Chr6
Chr7
Chr8
Chr9
Chr10
0
50
100
150
200
250
300
Mbp
1.0
0.8
0.6
0.4
0.2
0.0
0
1
relative gene density

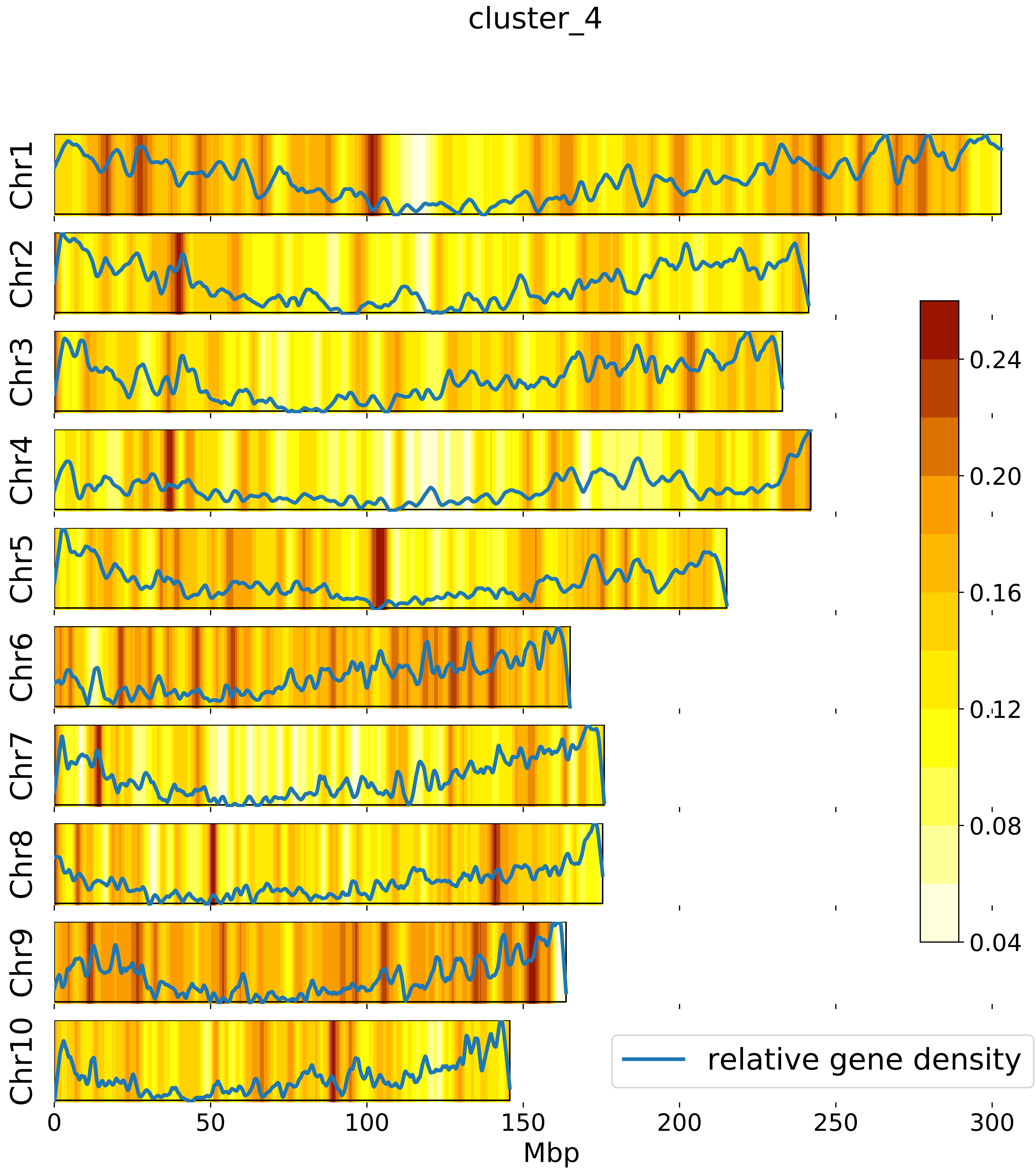
cluster_4
Chr1
Chr2
Chr3
Chr4
Chr5
Chr6
Chr7
Chr8
Chr9
Chr10
0
50
100
150
200
250
300
Mbp
0.24
0.20
0.16
0.12
0.08
0.04
relative gene density

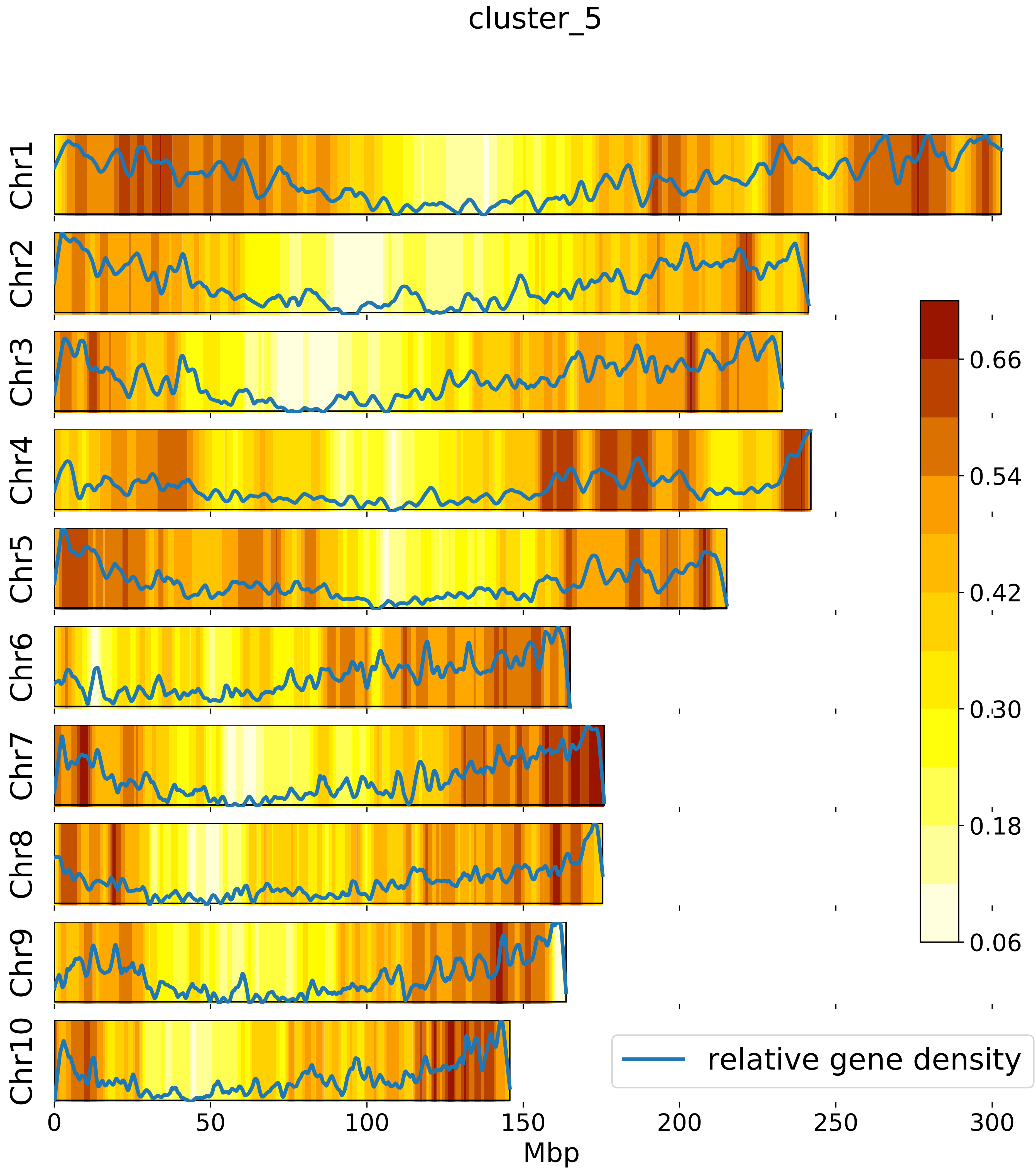
cluster_5
Chr1
Chr2
Chr3
Chr4
Chr5
Chr6
Chr7
Chr8
Chr9
Chr10
0.66
0.54
0.42
0.30
0.18
0.06
relative gene density
0
50
100
150
200
250
300
Mbp

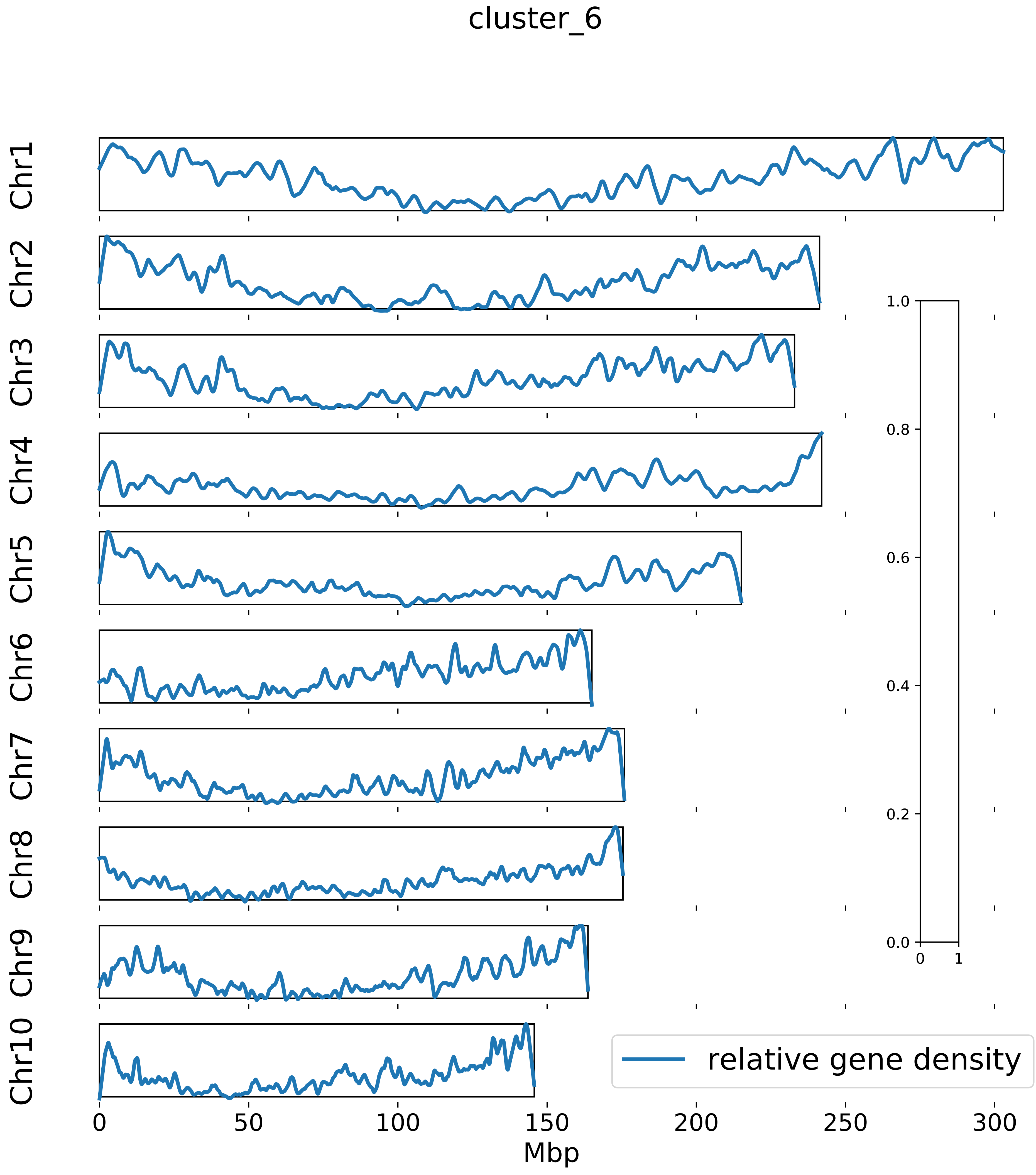
cluster_6
Chr1
Chr2
Chr3
Chr4
Chr5
Chr6
Chr7
Chr8
Chr9
Chr10
0
50
100
150
200
250
300
Mbp
relative gene density
1.0
0.8
0.6
0.4
0.2
0.0
0
1

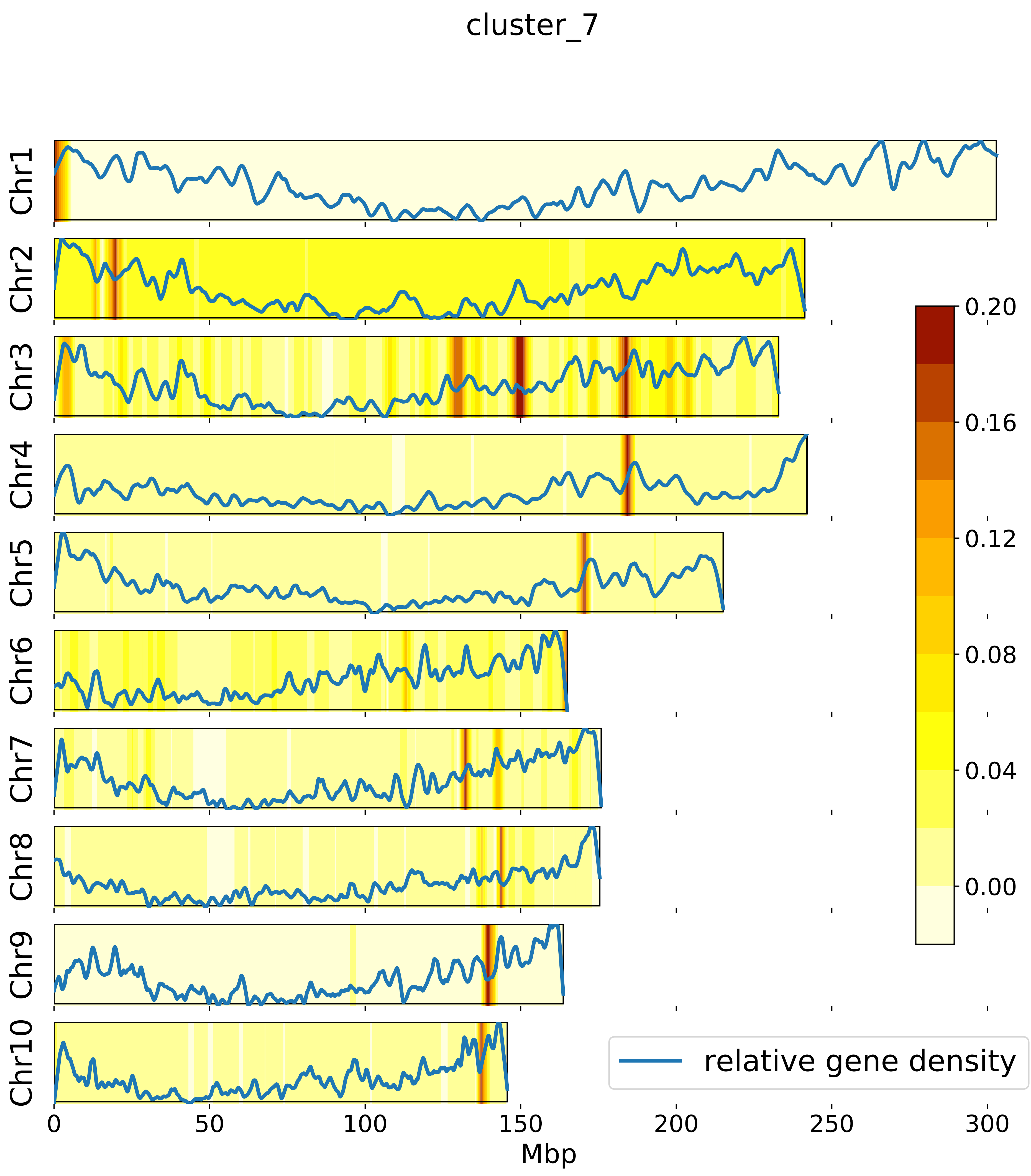

cluster_7
Chr1
Chr2
Chr3
Chr4
Chr5
Chr6
Chr7
Chr8
Chr9
Chr10
0
50
100
150
200
250
300
Mbp
0.20
0.16
0.12
0.08
0.04
0.00
relative gene density

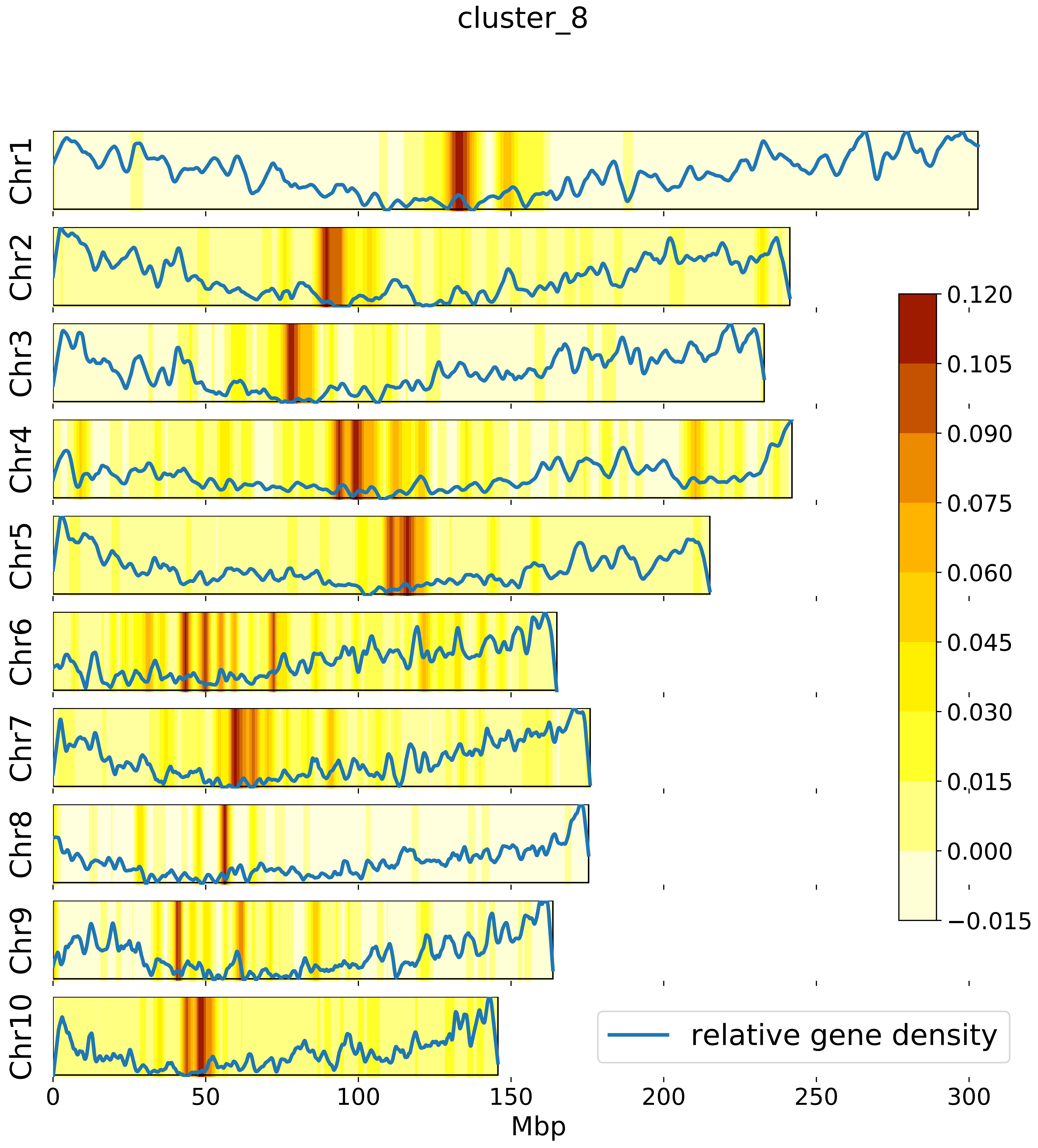

cluster_8
Chr1
Chr2
Chr3
Chr4
Chr5
Chr6
Chr7
Chr8
Chr9
Chr10
0
50
100
150
200
250
300
Mbp
0.120
0.105
0.090
0.075
0.060
0.045
0.030
0.015
0.000
−0.015
relative gene density

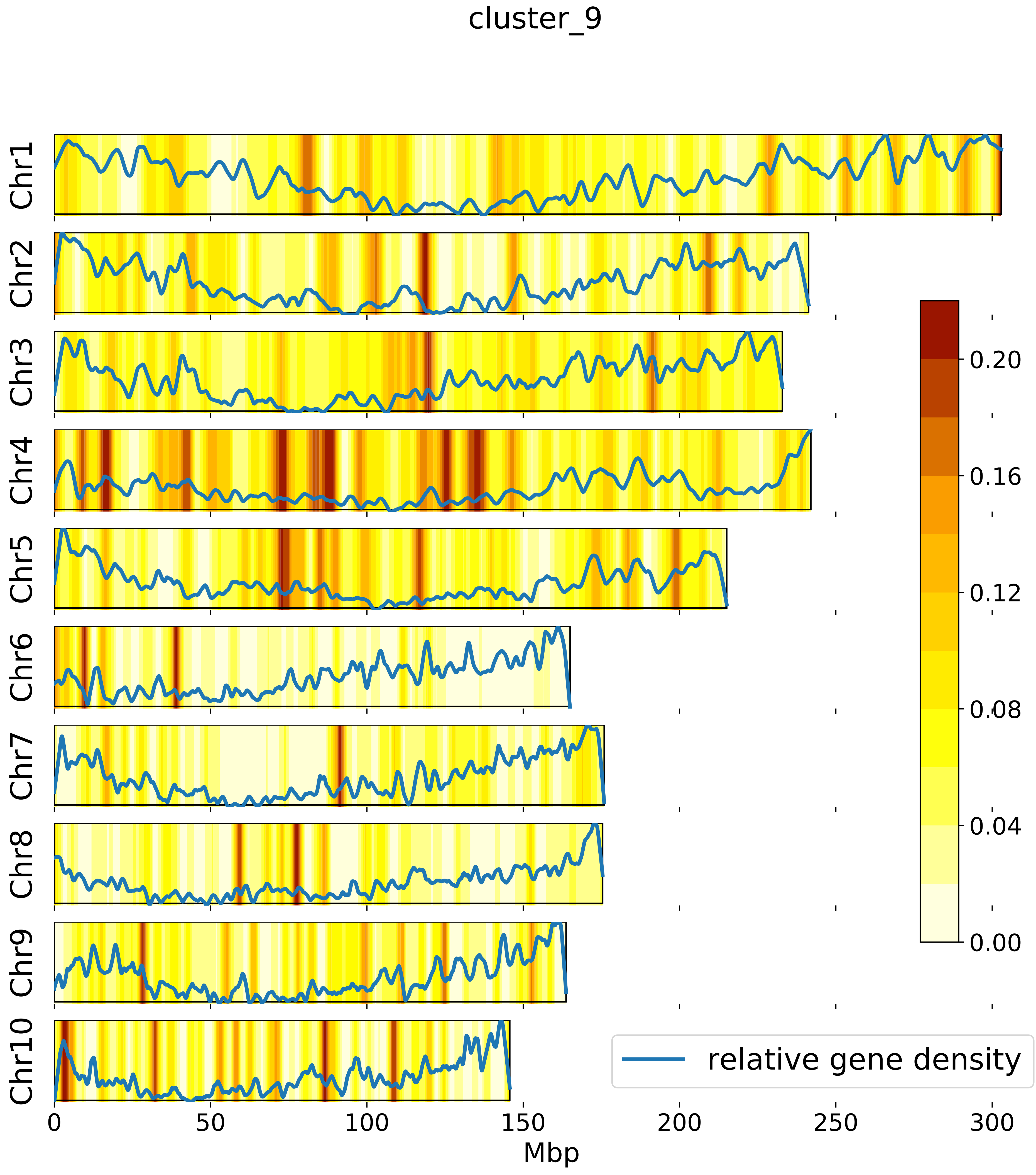

cluster_9
Chr1
Chr2
Chr3
Chr4
Chr5
Chr6
Chr7
Chr8
Chr9
Chr10
0
50
100
150
200
250
300
Mbp
0.20
0.16
0.12
0.08
0.04
0.00
relative gene density

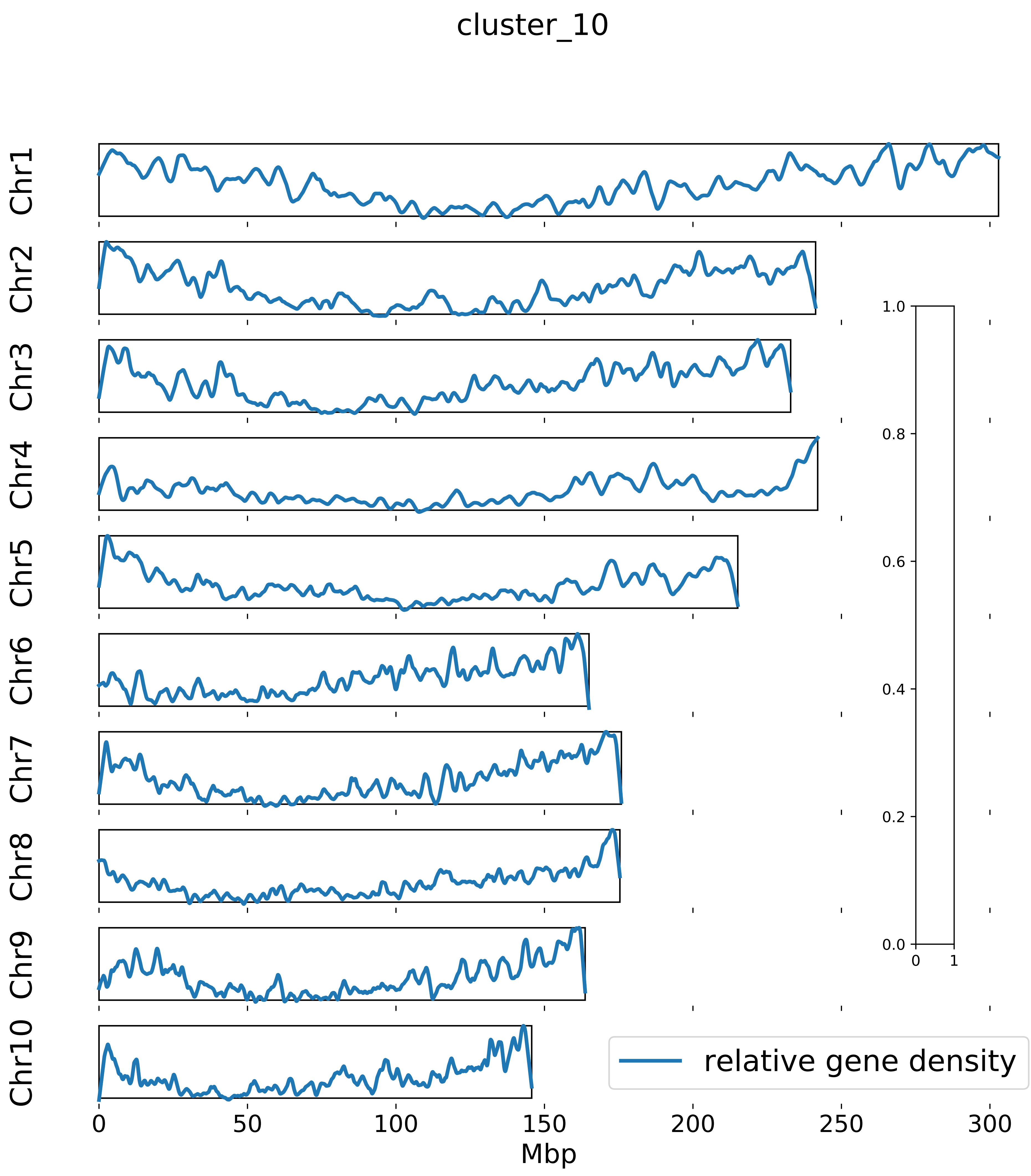
cluster_10
Chr1
Chr2
Chr3
Chr4
Chr5
Chr6
Chr7
Chr8
Chr9
Chr10
0
50
100
150
200
250
300
Mbp
1.0
0.8
0.6
0.4
0.2
0.0
0
1
relative gene density

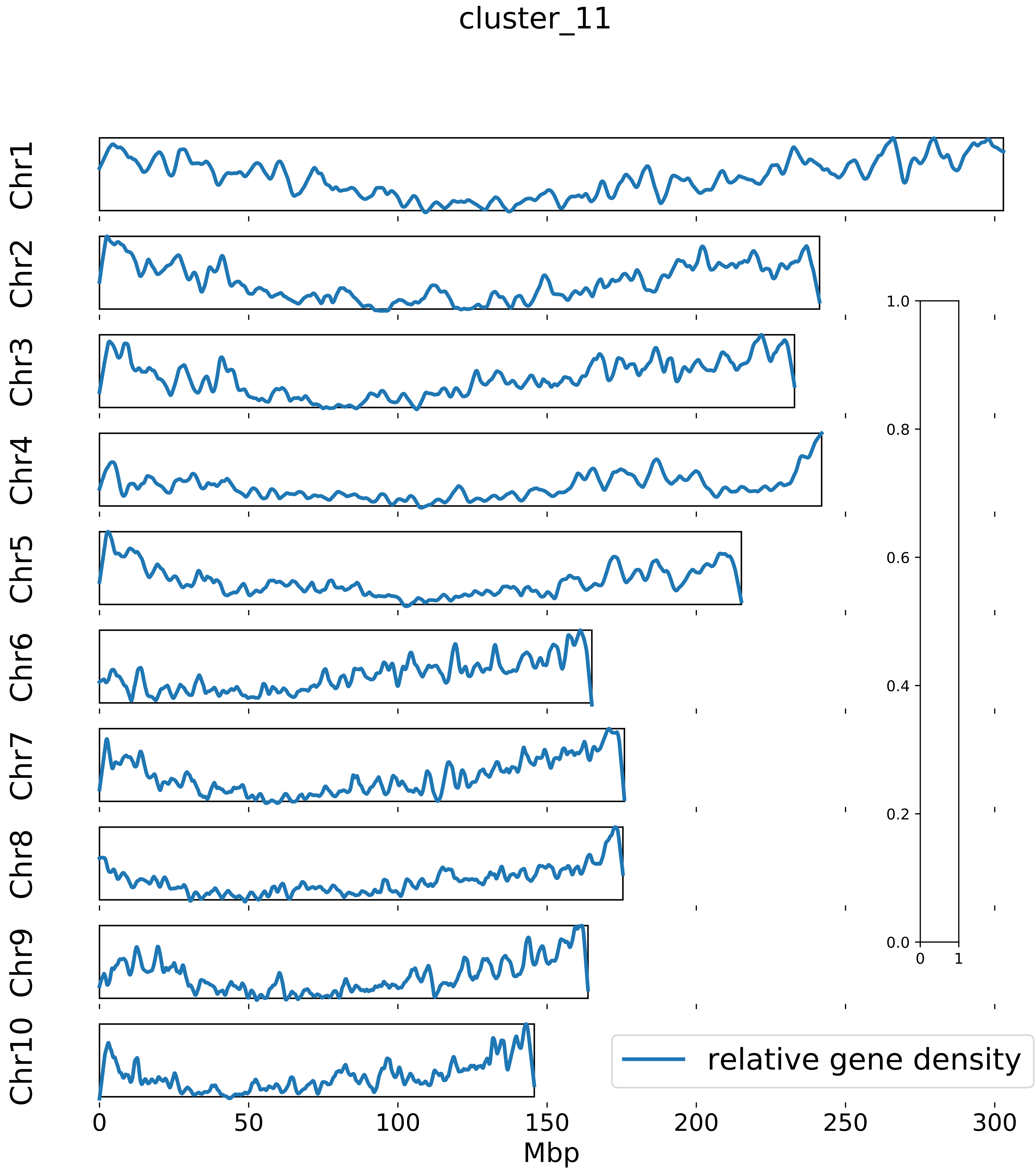
cluster_11
Chr1
Chr2
Chr3
Chr4
Chr5
Chr6
Chr7
Chr8
Chr9
Chr10
0
50
100
150
200
250
300
Mbp
1.0
0.8
0.6
0.4
0.2
0.0
0
1
relative gene density

cluster_12

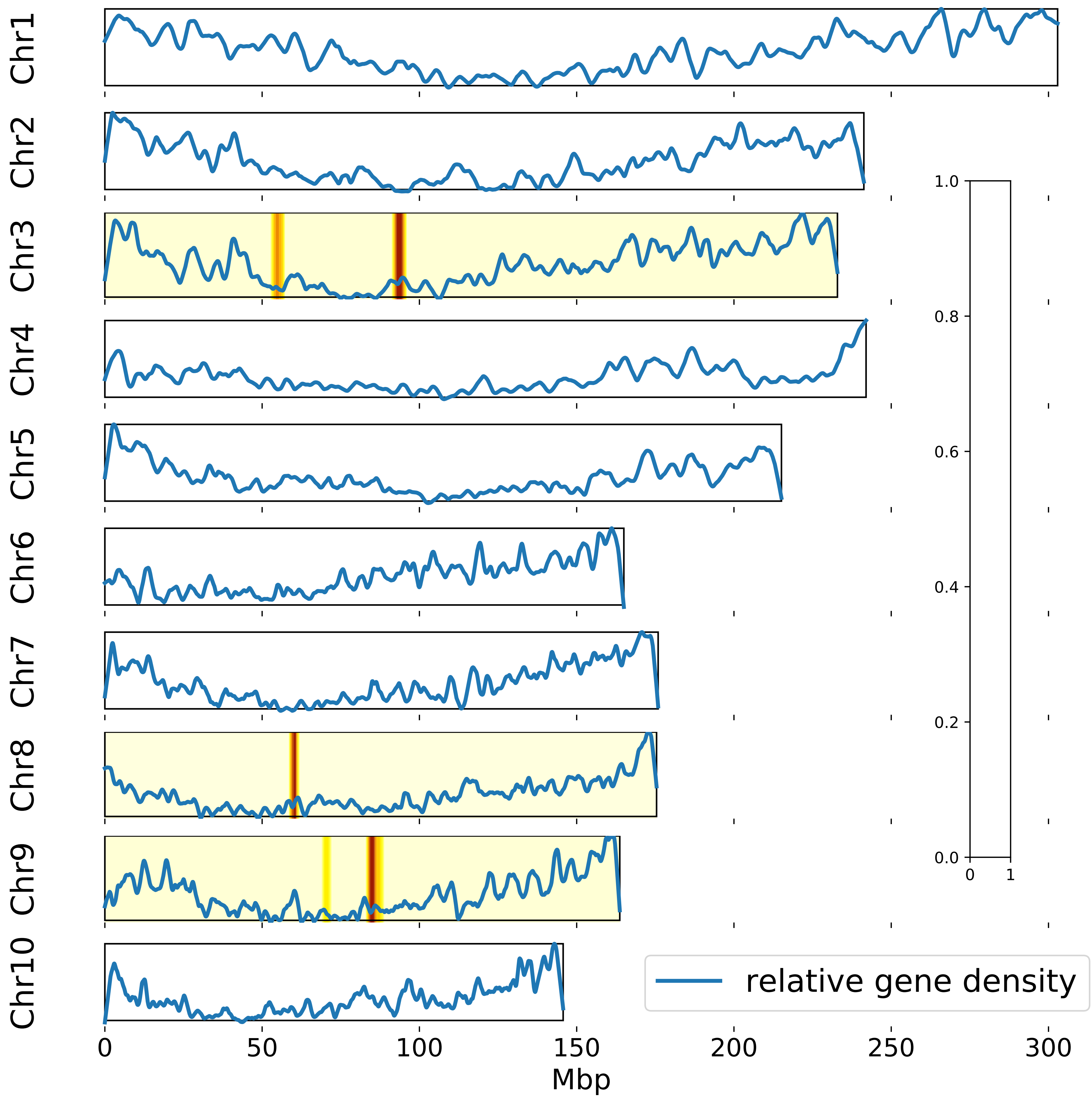

Chr1
Chr2
Chr3
Chr4
Chr5
Chr6
Chr7
Chr8
Chr9
Chr10
0
50
100
150
200
250
300
Mbp
relative gene density
1.0
0.8
0.6
0.4
0.2
0.0
0
1

cluster_13

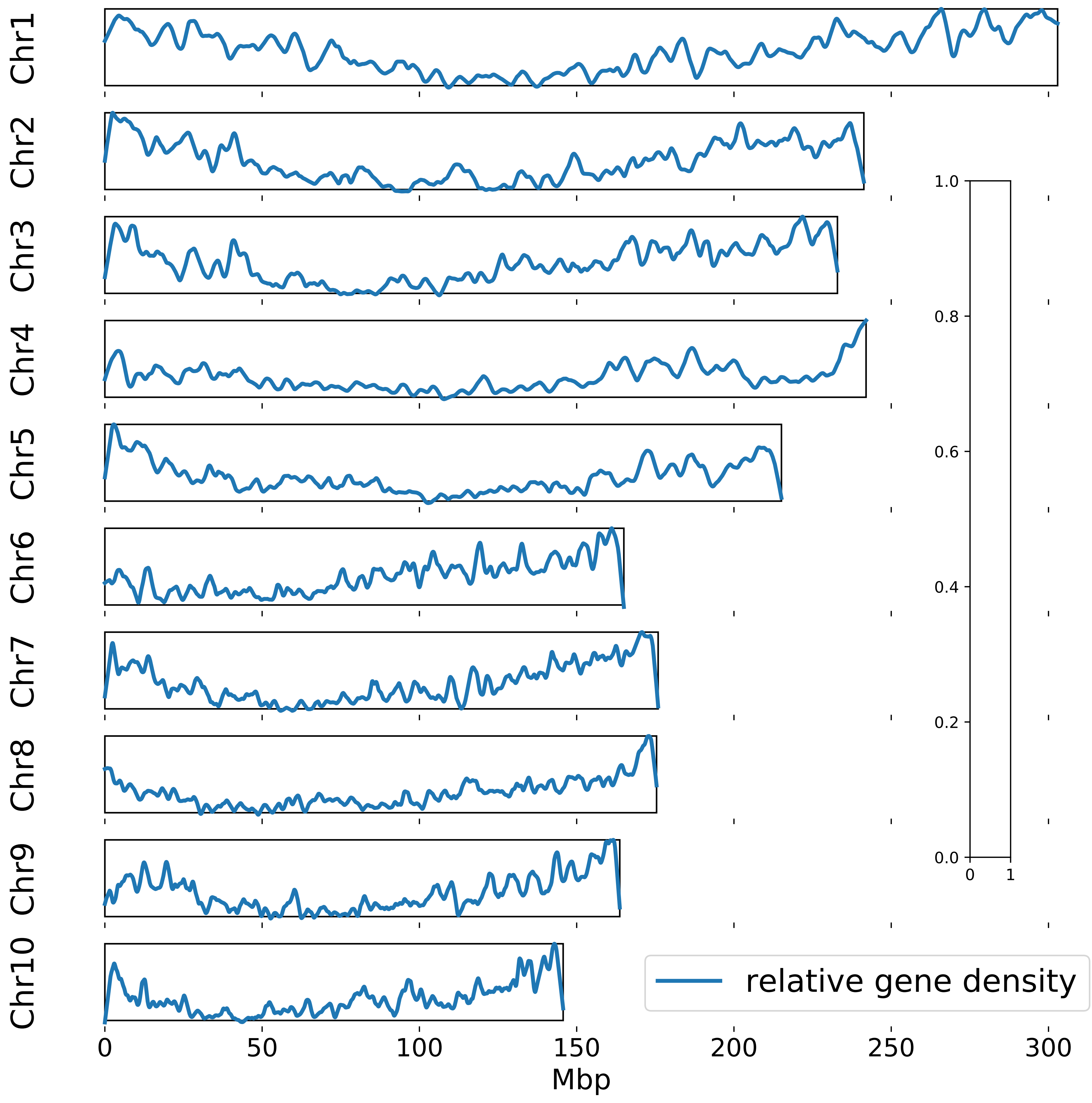
Chr1
Chr2
Chr3
Chr4
Chr5
Chr6
Chr7
Chr8
Chr9
Chr10
0
50
100
150
200
250
300
Mbp
1.0
0.8
0.6
0.4
0.2
0.0
0
1
relative gene density

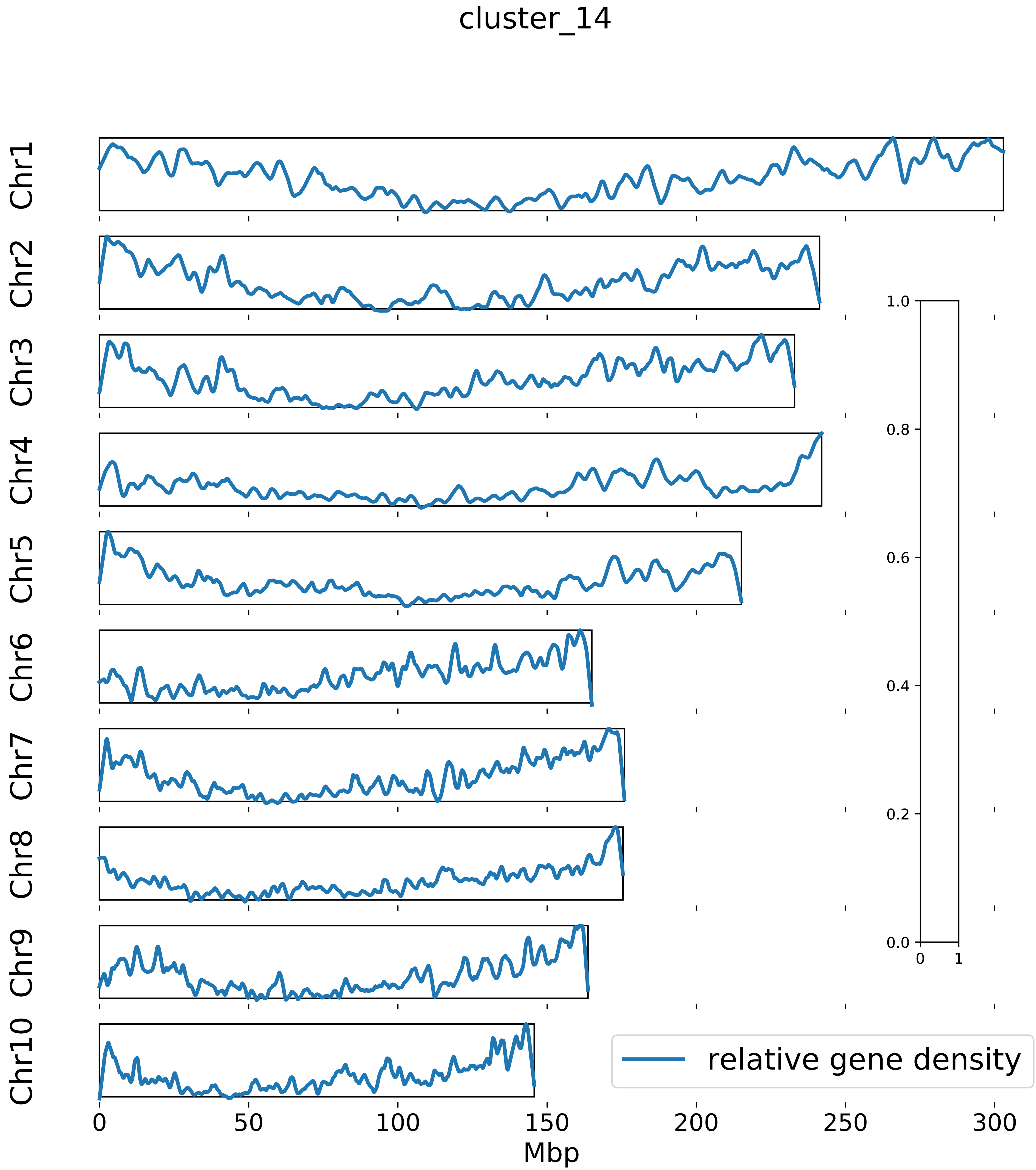
cluster_14
Chr1
Chr2
Chr3
Chr4
Chr5
Chr6
Chr7
Chr8
Chr9
Chr10
0
50
100
150
200
250
300
Mbp
1.0
0.8
0.6
0.4
0.2
0.0
0
1
relative gene density

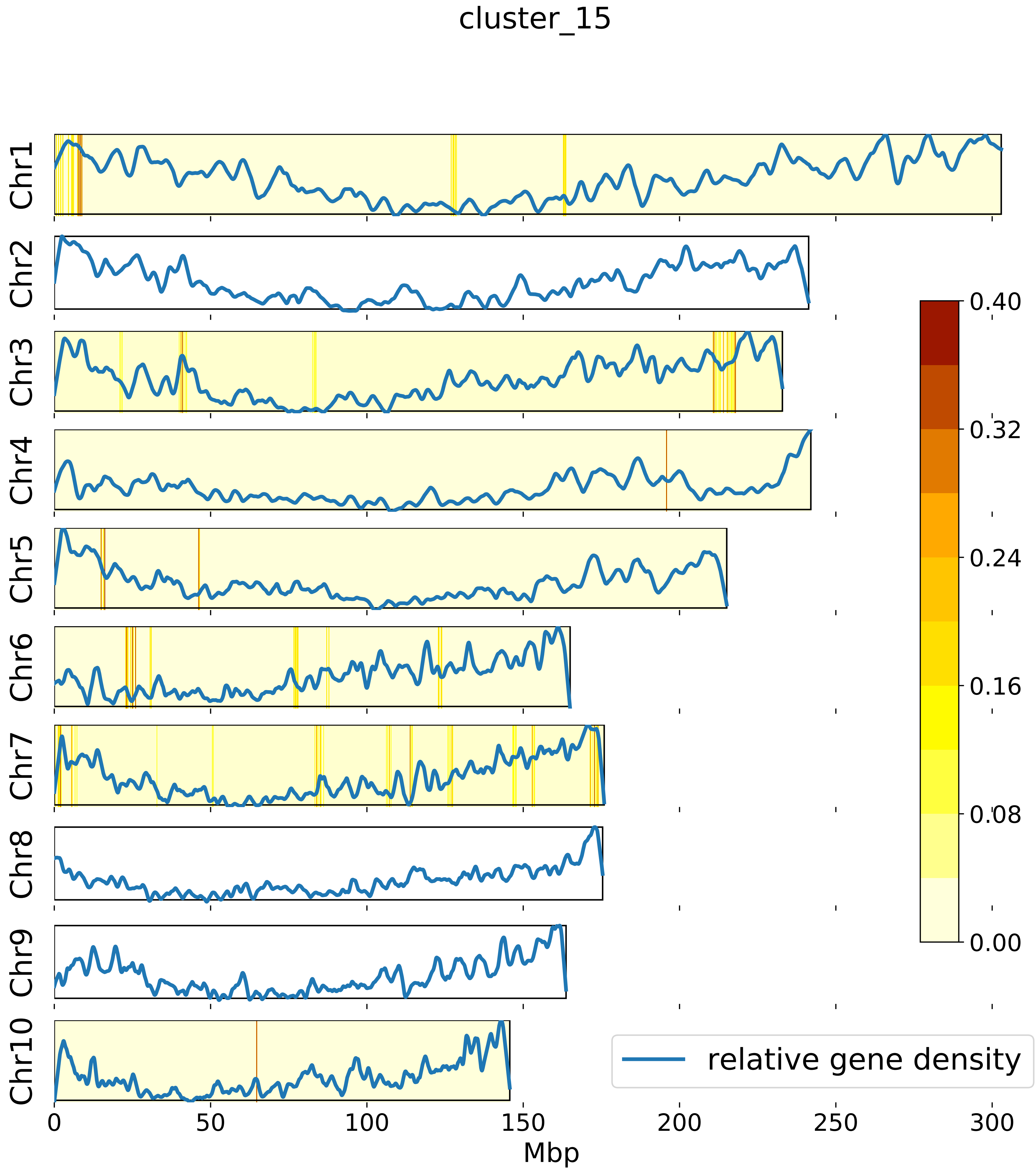
cluster_15
Chr1
Chr2
Chr3
Chr4
Chr5
Chr6
Chr7
Chr8
Chr9
Chr10
0
50
100
150
200
250
300
Mbp
0.40
0.32
0.24
0.16
0.08
0.00
relative gene density

cluster_16

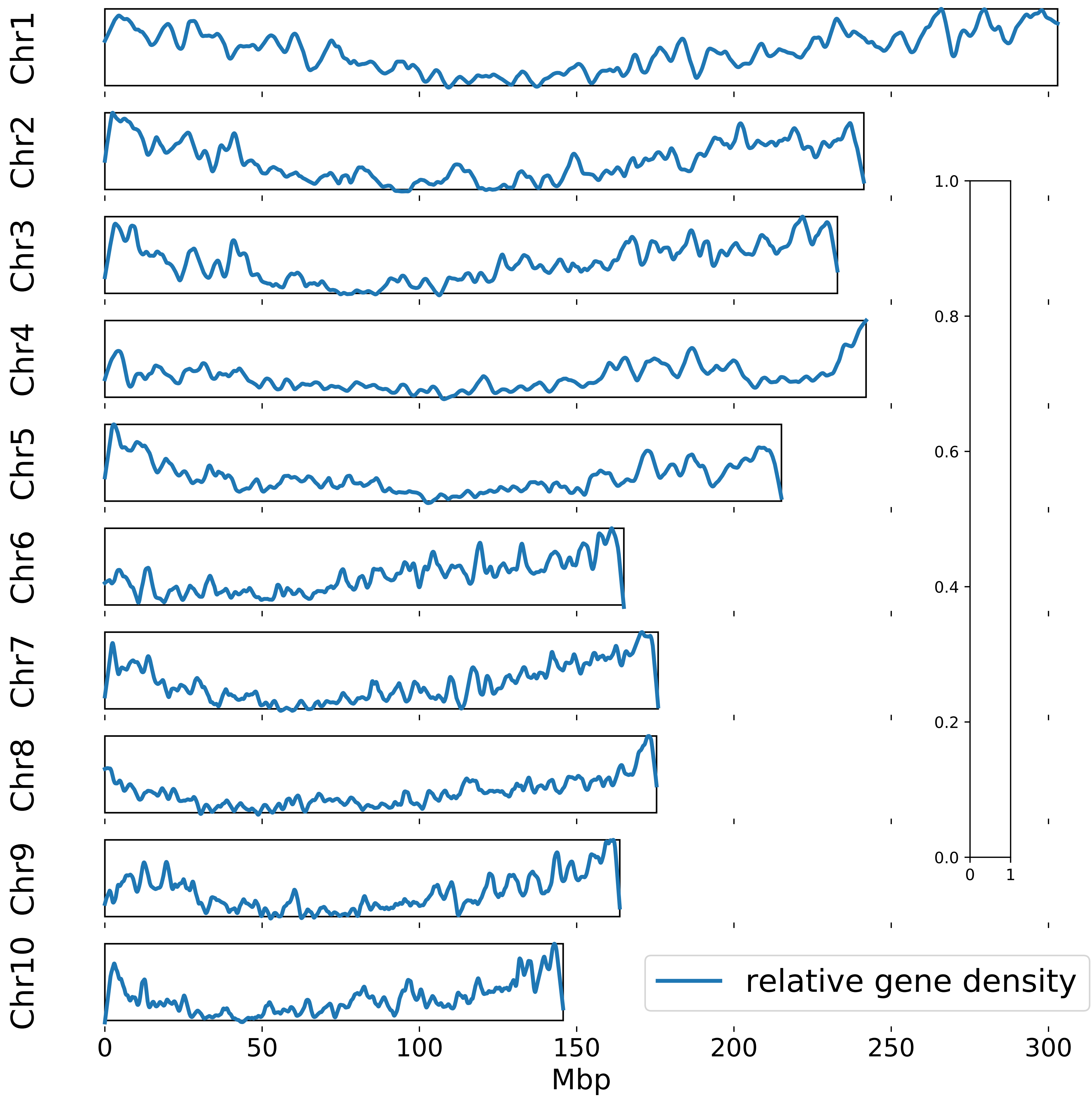

Chr1
Chr2
Chr3
Chr4
Chr5
Chr6
Chr7
Chr8
Chr9
Chr10
0
50
100
150
200
250
300
Mbp
1.0
0.8
0.6
0.4
0.2
0.0
0
1
relative gene density

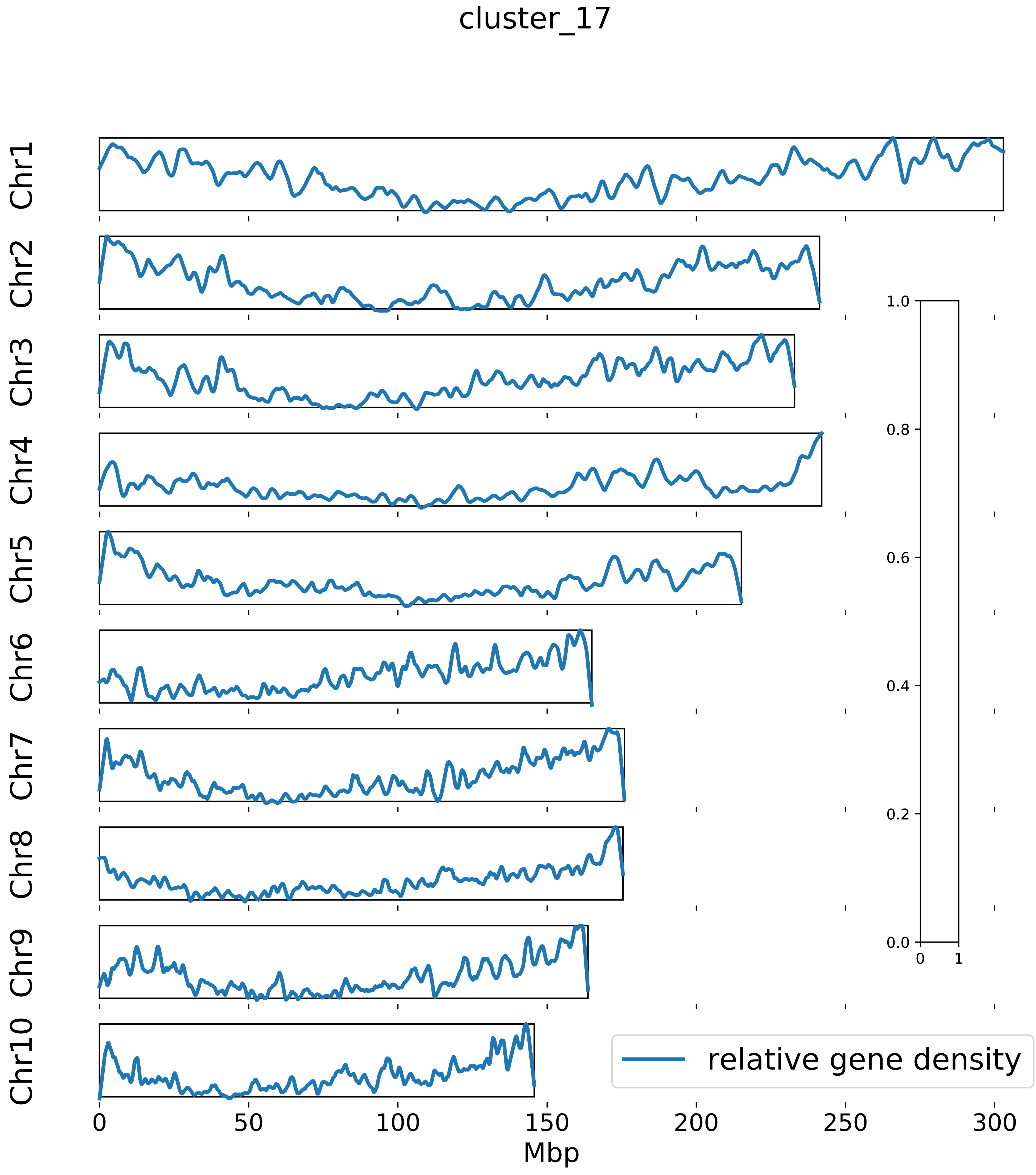
cluster_17
Chr1
Chr2
Chr3
Chr4
Chr5
Chr6
Chr7
Chr8
Chr9
Chr10
0
50
100
150
200
250
300
Mbp
1.0
0.8
0.6
0.4
0.2
0.0
0
1
relative gene density

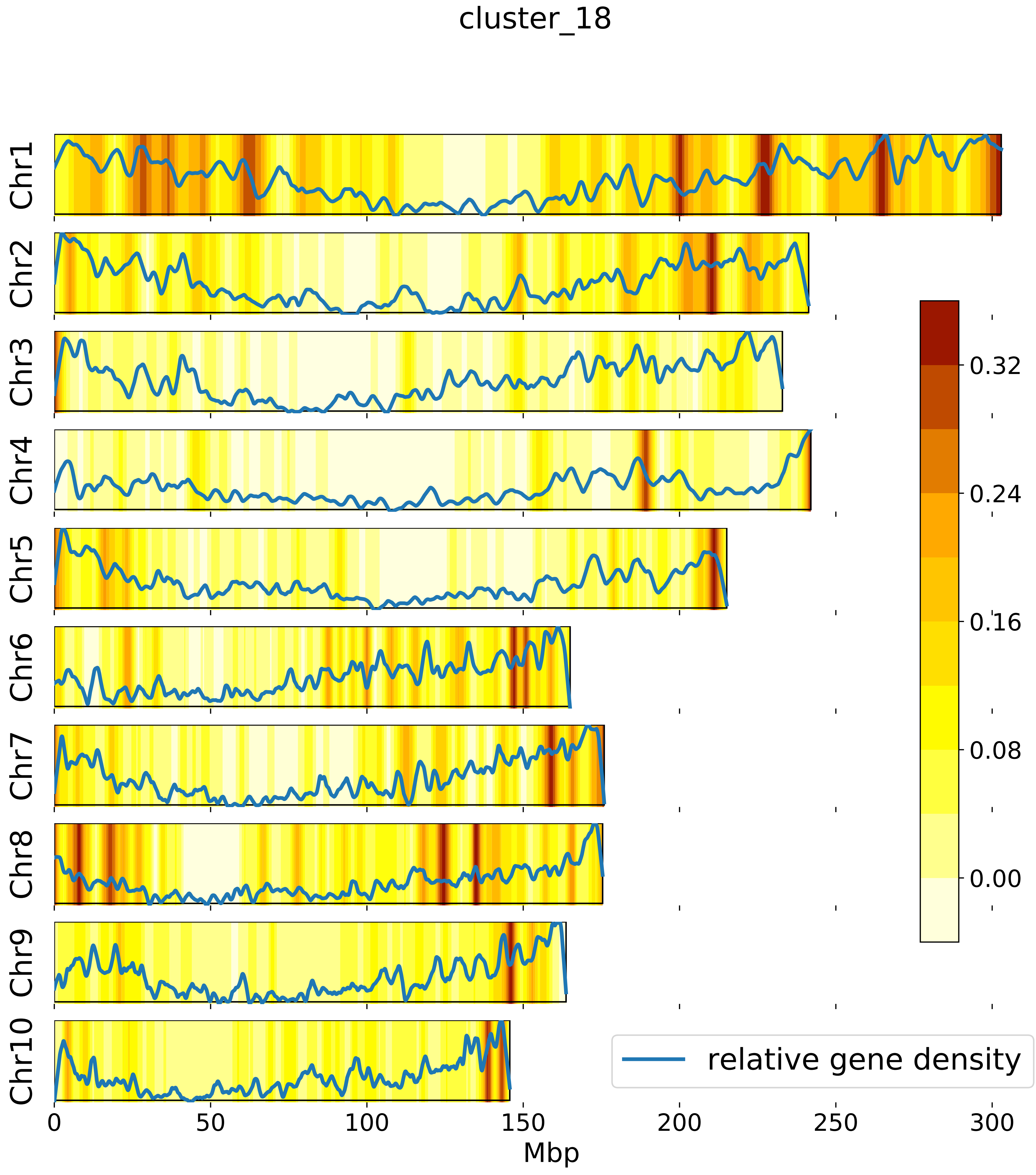
cluster_18
Chr1
Chr2
Chr3
Chr4
Chr5
Chr6
Chr7
Chr8
Chr9
Chr10
0
50
100
150
200
250
300
Mbp
0.32
0.24
0.16
0.08
0.00
relative gene density

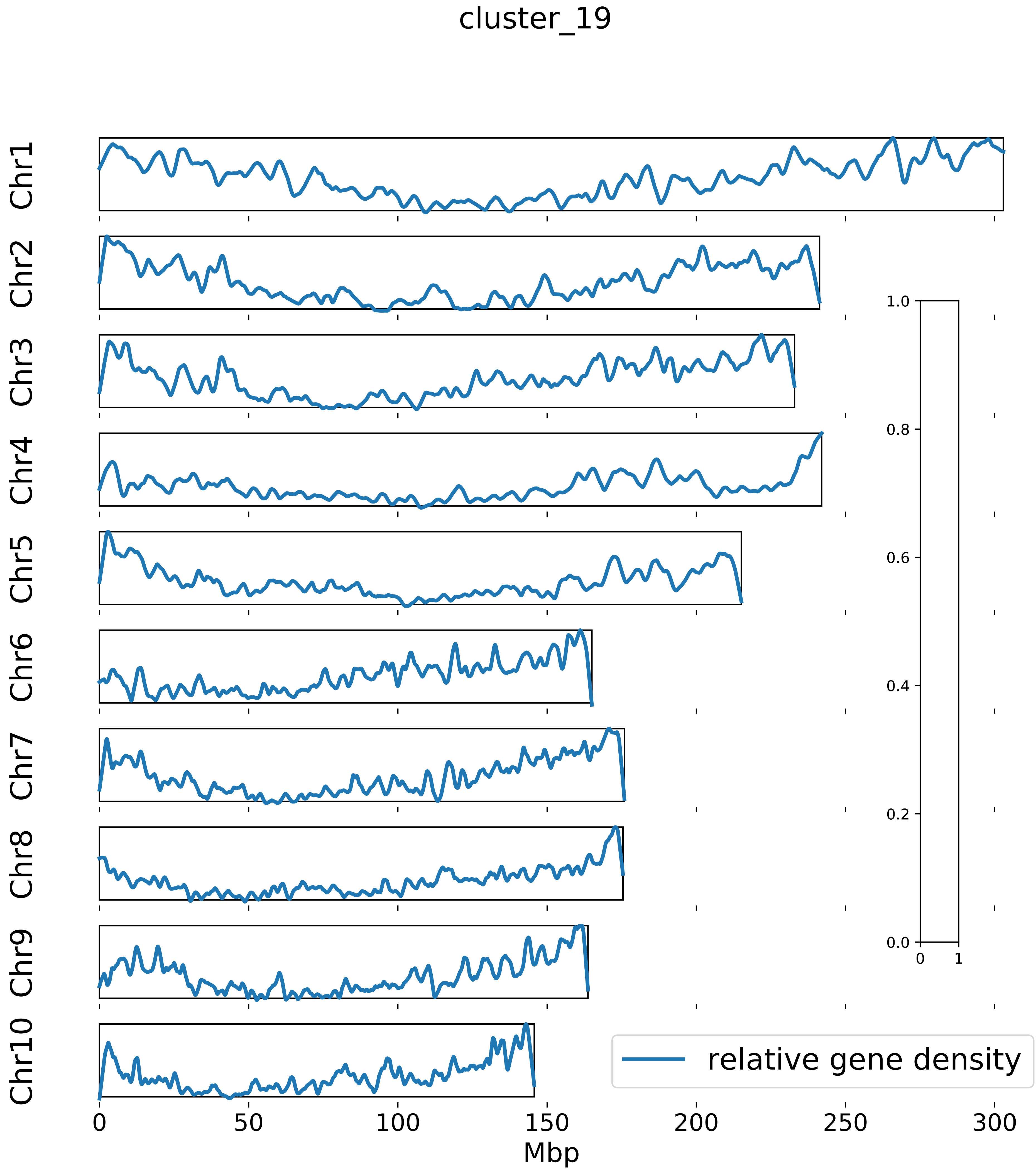
cluster_19
Chr1
Chr2
Chr3
Chr4
Chr5
Chr6
Chr7
Chr8
Chr9
Chr10
1.0
0.8
0.6
0.4
0.2
0.0
0
1
relative gene density
0
50
100
150
200
250
300
Mbp

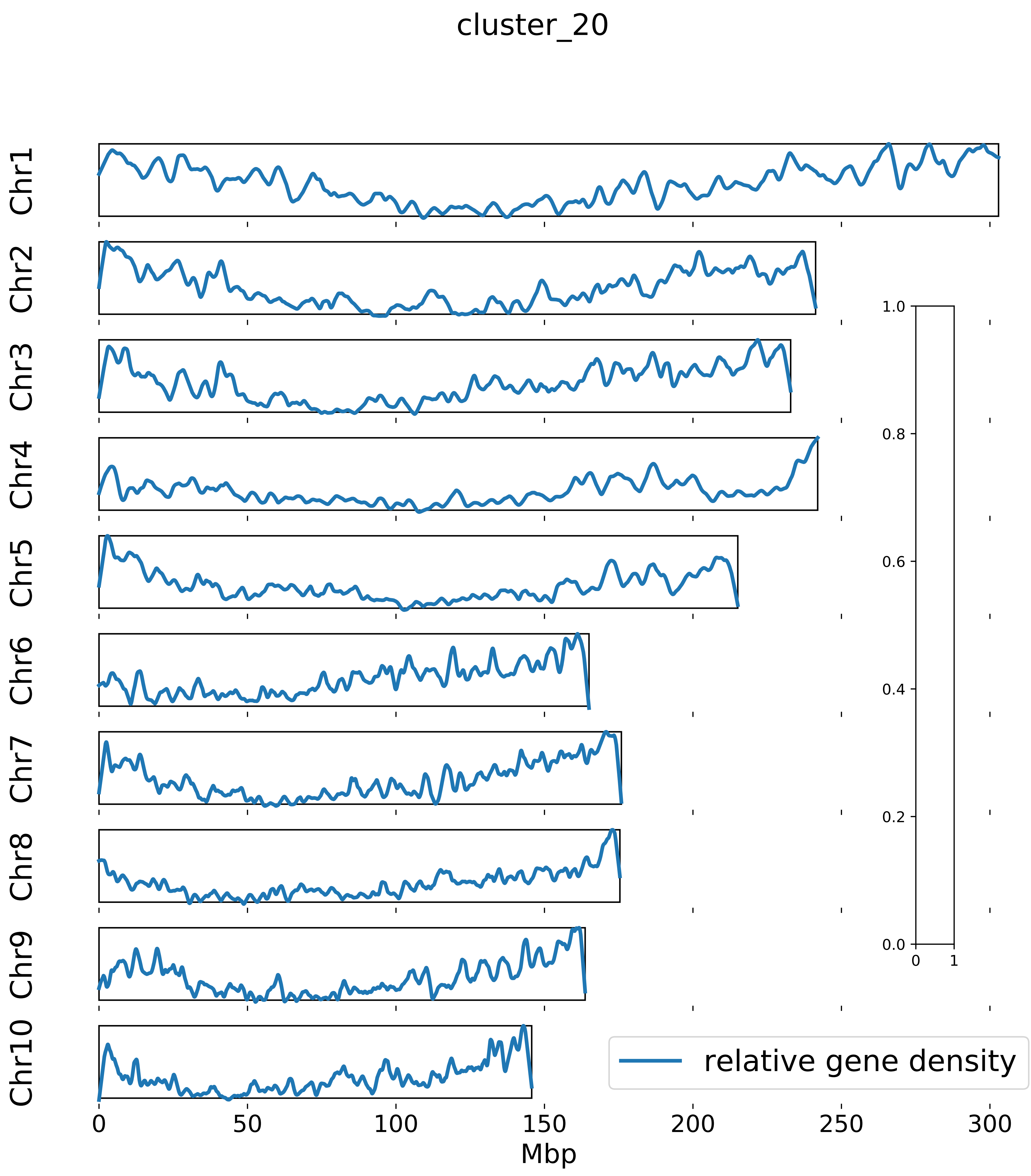
cluster_20
Chr1
Chr2
Chr3
Chr4
Chr5
Chr6
Chr7
Chr8
Chr9
Chr10
0
50
100
150
200
250
300
Mbp
1.0
0.8
0.6
0.4
0.2
0.0
0
1
relative gene density

cluster_21

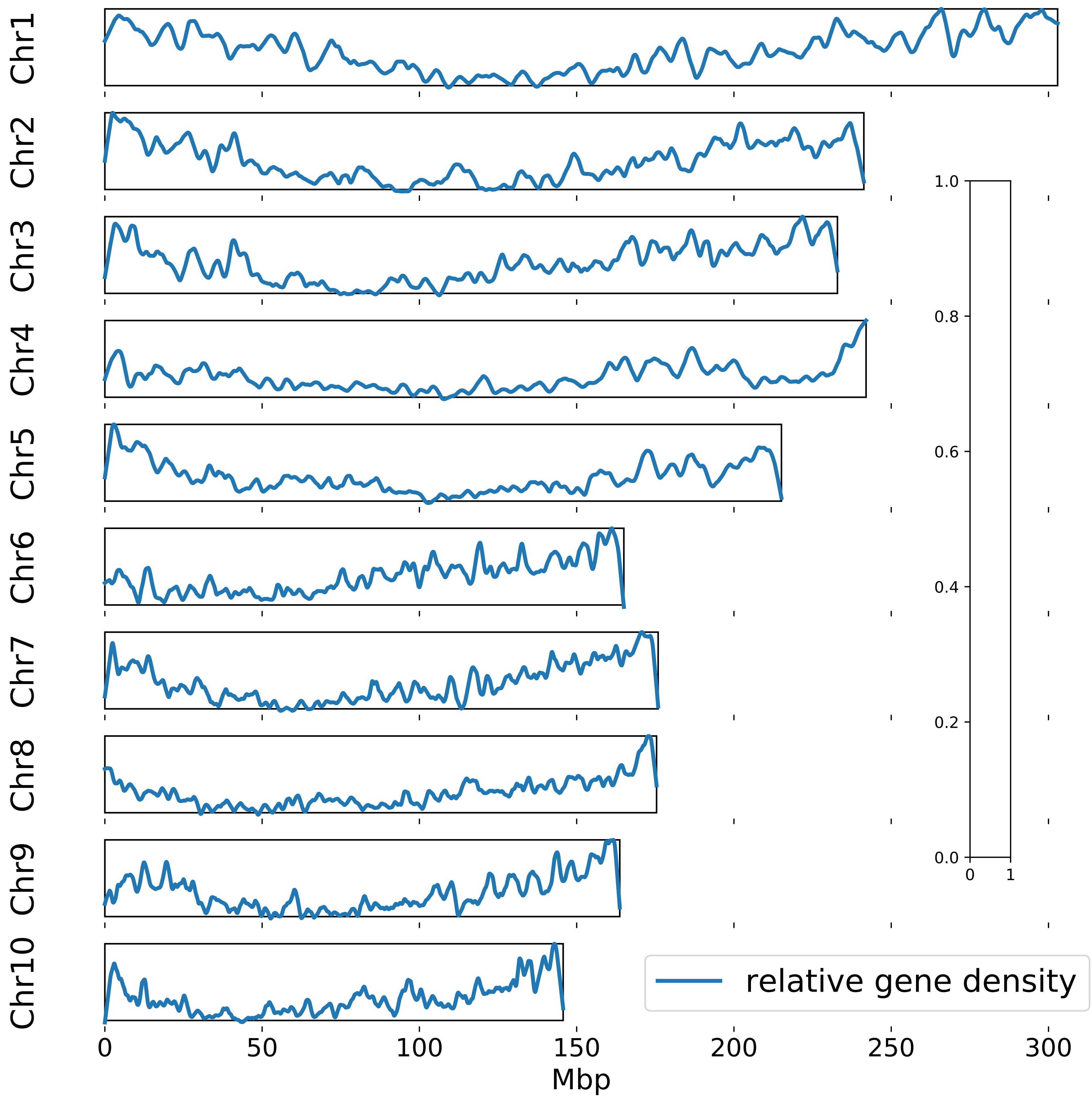
Chr1
Chr2
Chr3
Chr4
Chr5
Chr6
Chr7
Chr8
Chr9
Chr10
0
50
100
150
200
250
300
Mbp
1.0
0.8
0.6
0.4
0.2
0.0
0
1
relative gene density

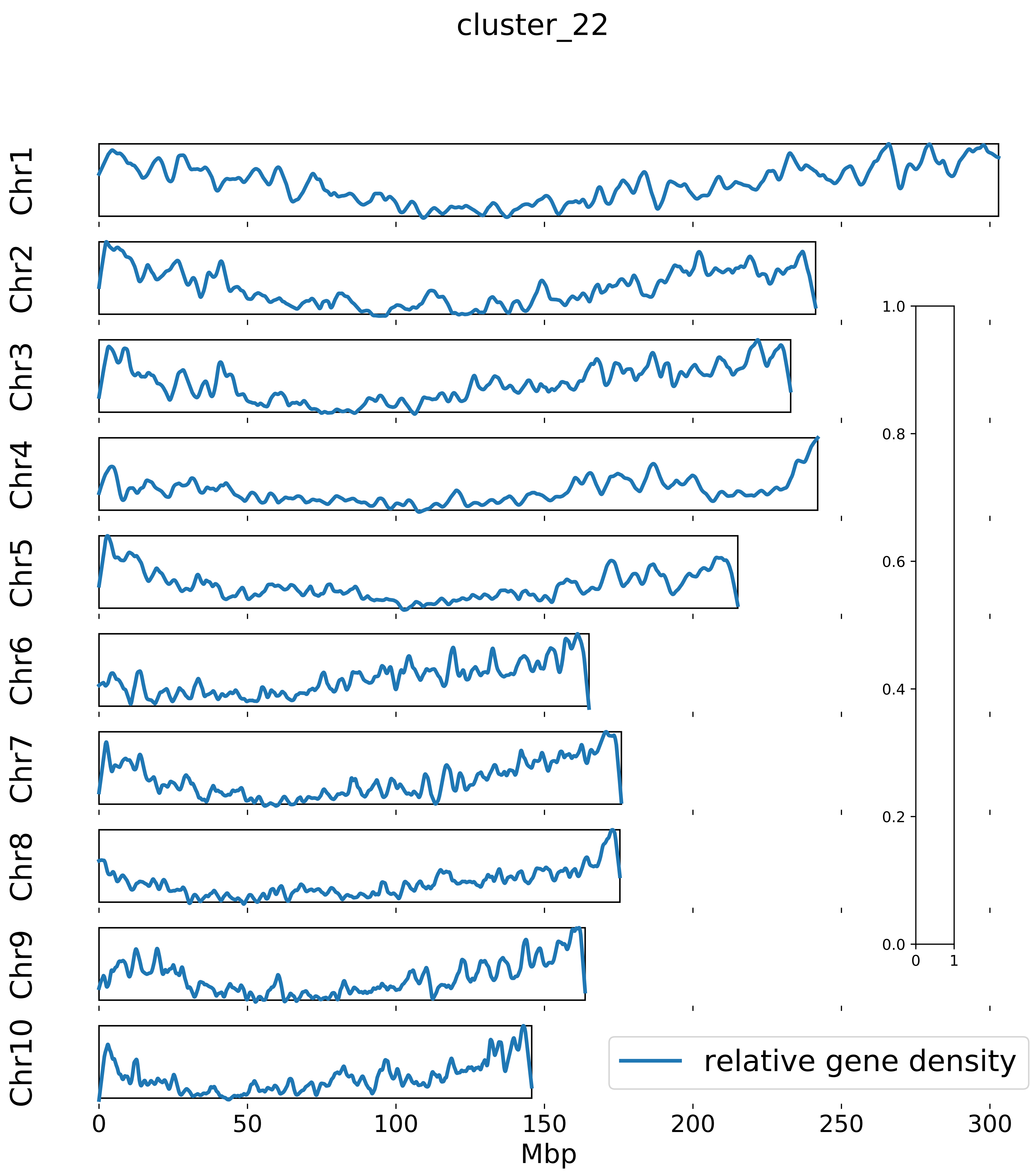

cluster_22
Chr1
Chr2
Chr3
Chr4
Chr5
Chr6
Chr7
Chr8
Chr9
Chr10
0
50
100
150
200
250
300
Mbp
1.0
0.8
0.6
0.4
0.2
0.0
0
1
relative gene density

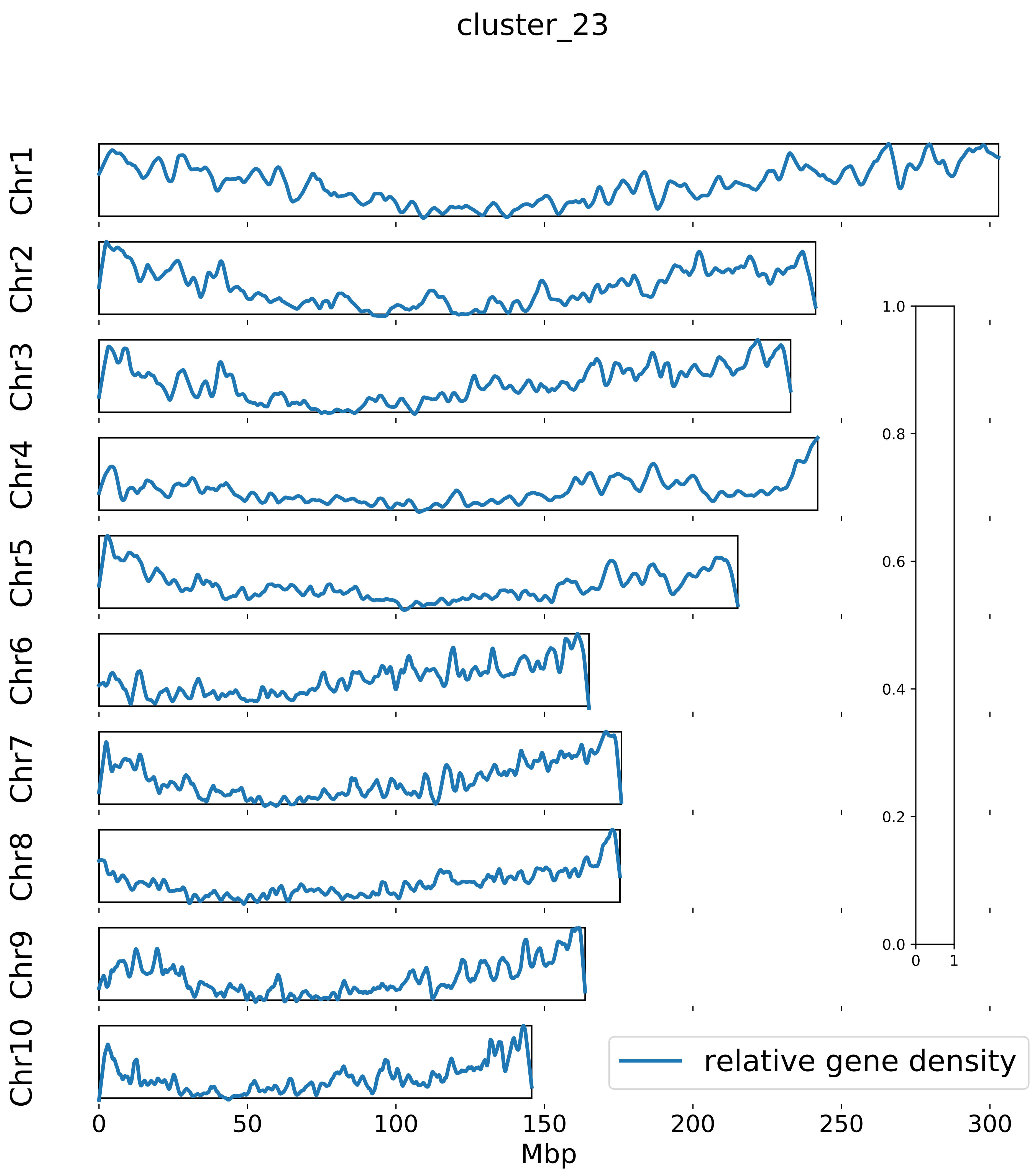
cluster_23
Chr1
Chr2
Chr3
Chr4
Chr5
Chr6
Chr7
Chr8
Chr9
Chr10
0
50
100
150
200
250
300
Mbp
1.0
0.8
0.6
0.4
0.2
0.0
0
1
relative gene density

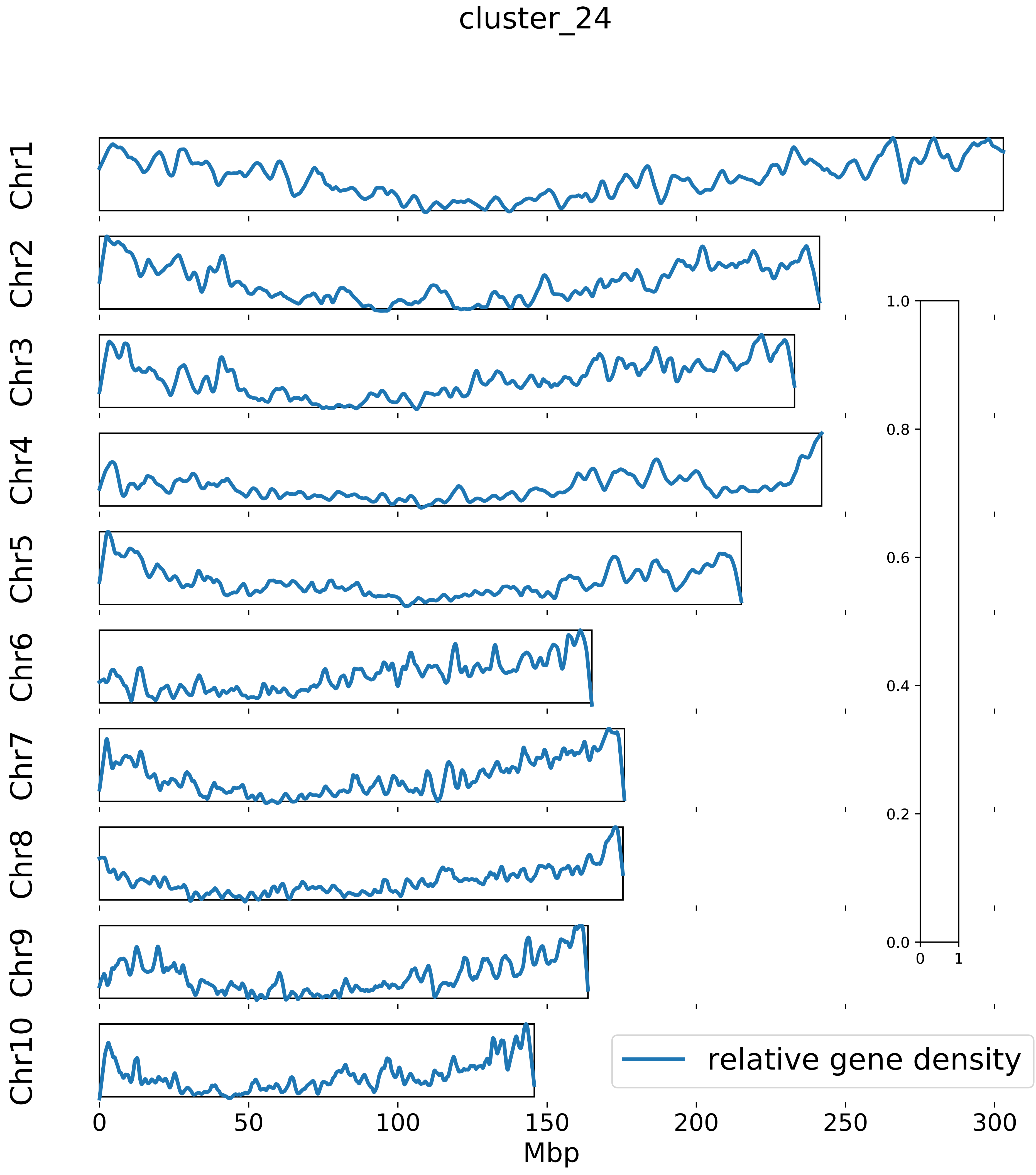

cluster_24
Chr1
Chr2
Chr3
Chr4
Chr5
Chr6
Chr7
Chr8
Chr9
Chr10
0
50
100
150
200
250
300
Mbp
1.0
0.8
0.6
0.4
0.2
0.0
0
1
relative gene density
