# Supplementary material for: Repetitive DNA content in the maize genome is uncoupled from population stratification at SNP loci
Source: BMC Genomics. 2020 Jan 30;21:98. doi: 10.1186/s12864-020-6517-0 (PMC6993463; doi:10.1186/s12864-020-6517-0)
Supplement: Supplementary file 7 — Additional file 7 Genome-wide kmer distribution in mo17. The genome-wide K-mer distribution for each of the 25 largest repeat clusters. A Kernel density estimation is indicated by color, and associated gene density estimates are also given. [file 12864_2020_6517_MOESM7_ESM.pdf]

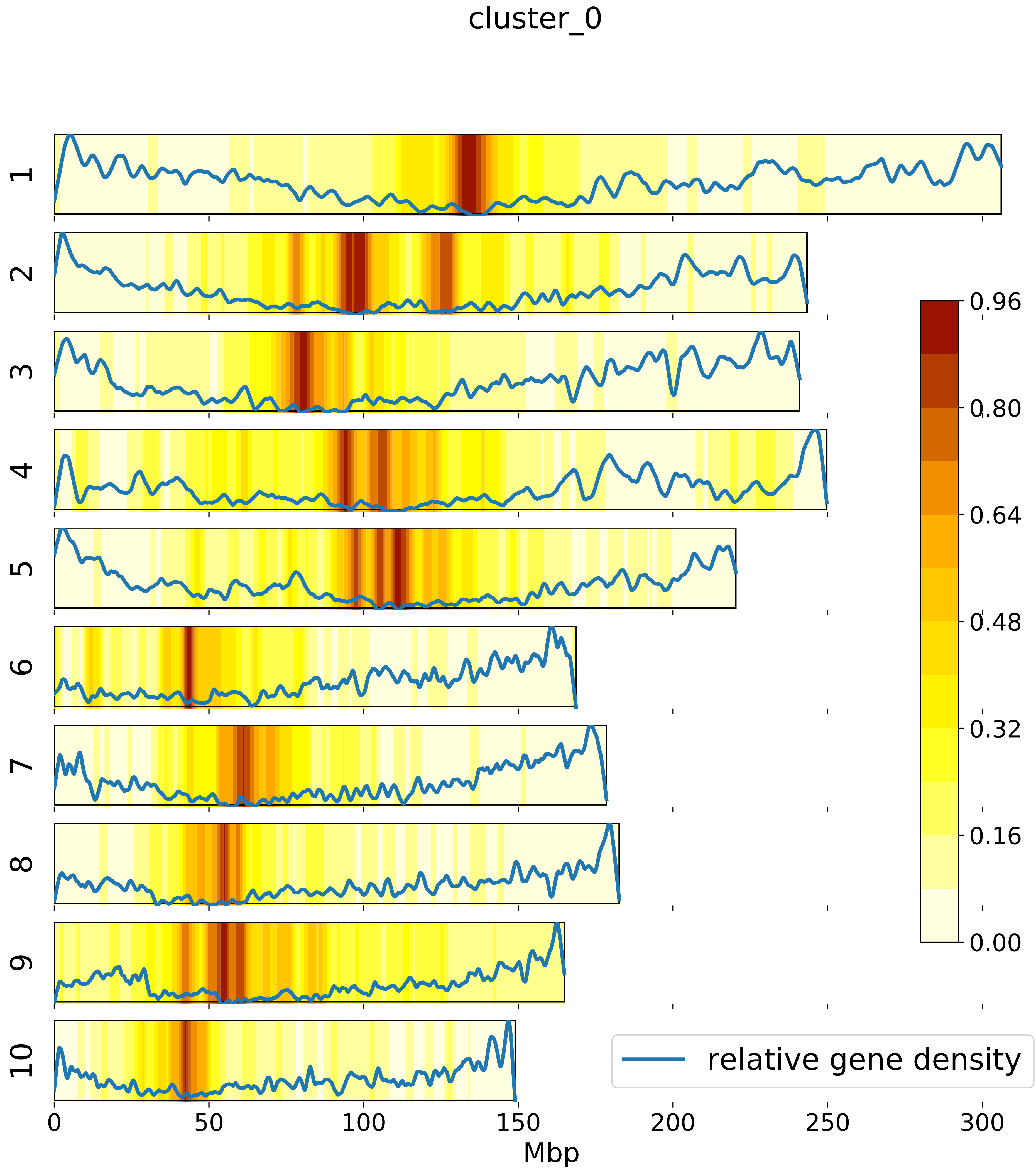

cluster_0
1
2
3
4
5
6
7
8
9
10
0
50
100
150
200
250
300
Mbp
0.96
0.80
0.64
0.48
0.32
0.16
0.00
relative gene density

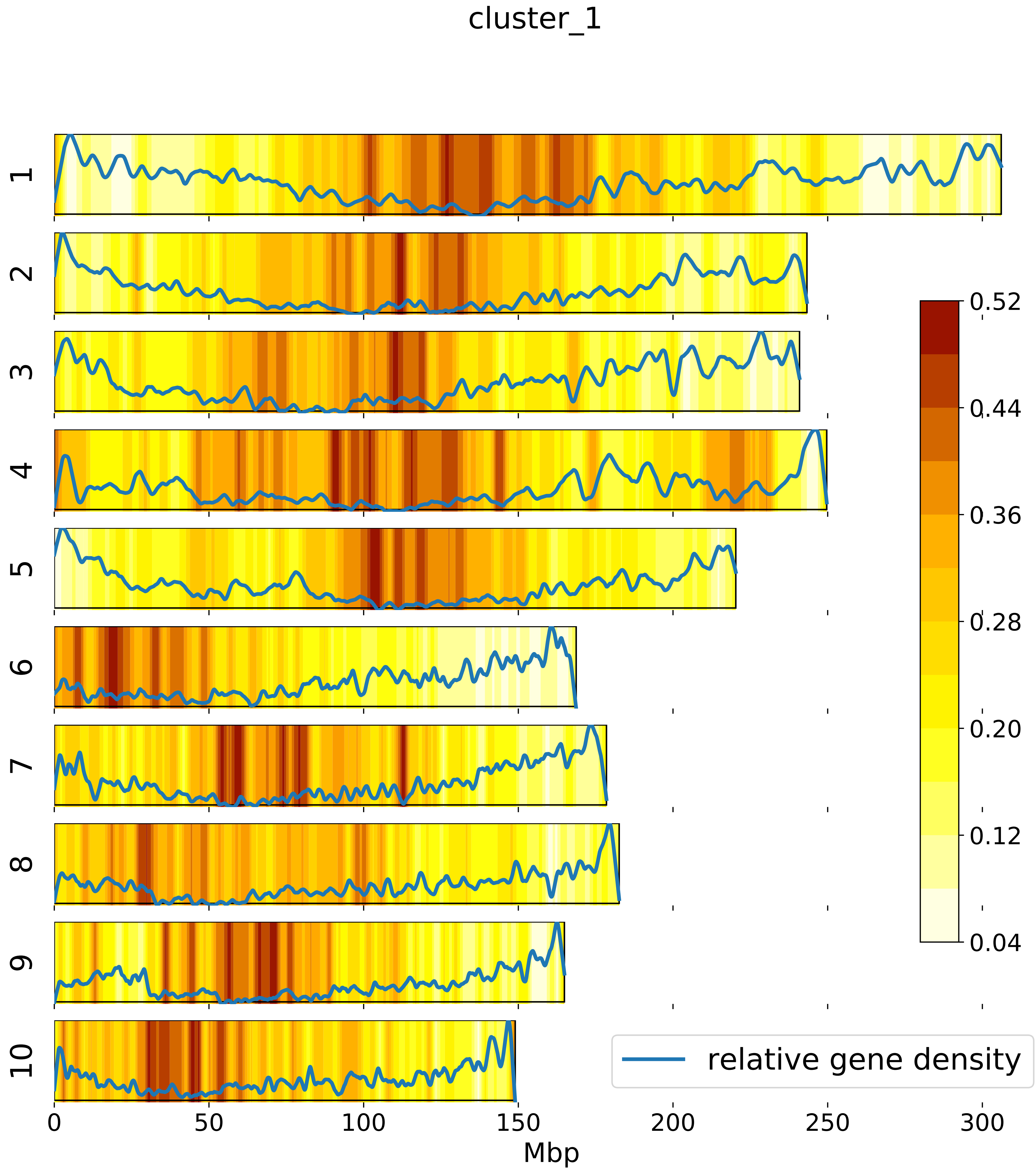

cluster_1
1
2
3
4
5
6
7
8
9
10
0
50
100
150
200
250
300
Mbp
0.52
0.44
0.36
0.28
0.20
0.12
0.04
relative gene density

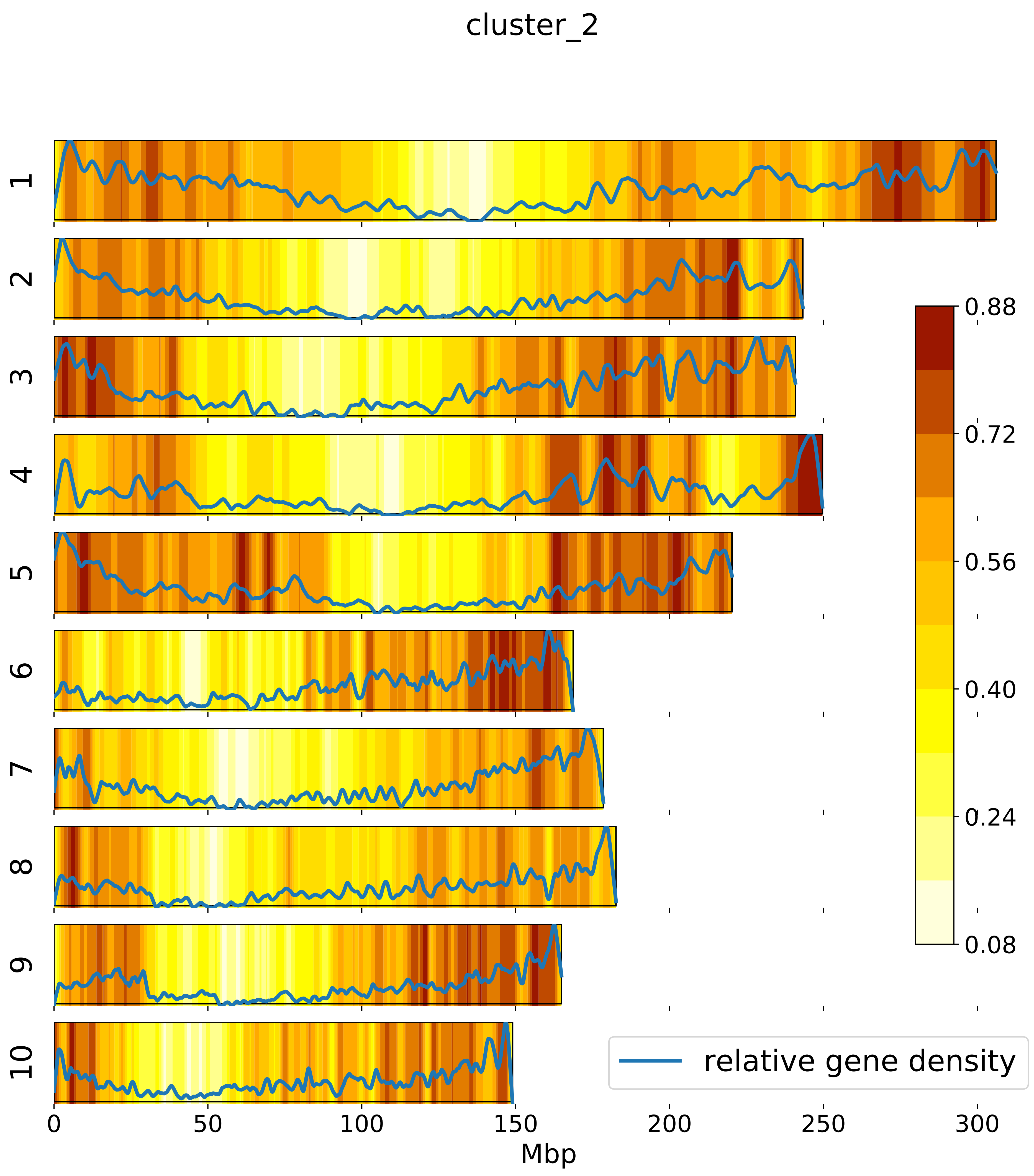

cluster_2
1
2
3
4
5
6
7
8
9
10
0
50
100
150
200
250
300
Mbp
0.88
0.72
0.56
0.40
0.24
0.08
relative gene density

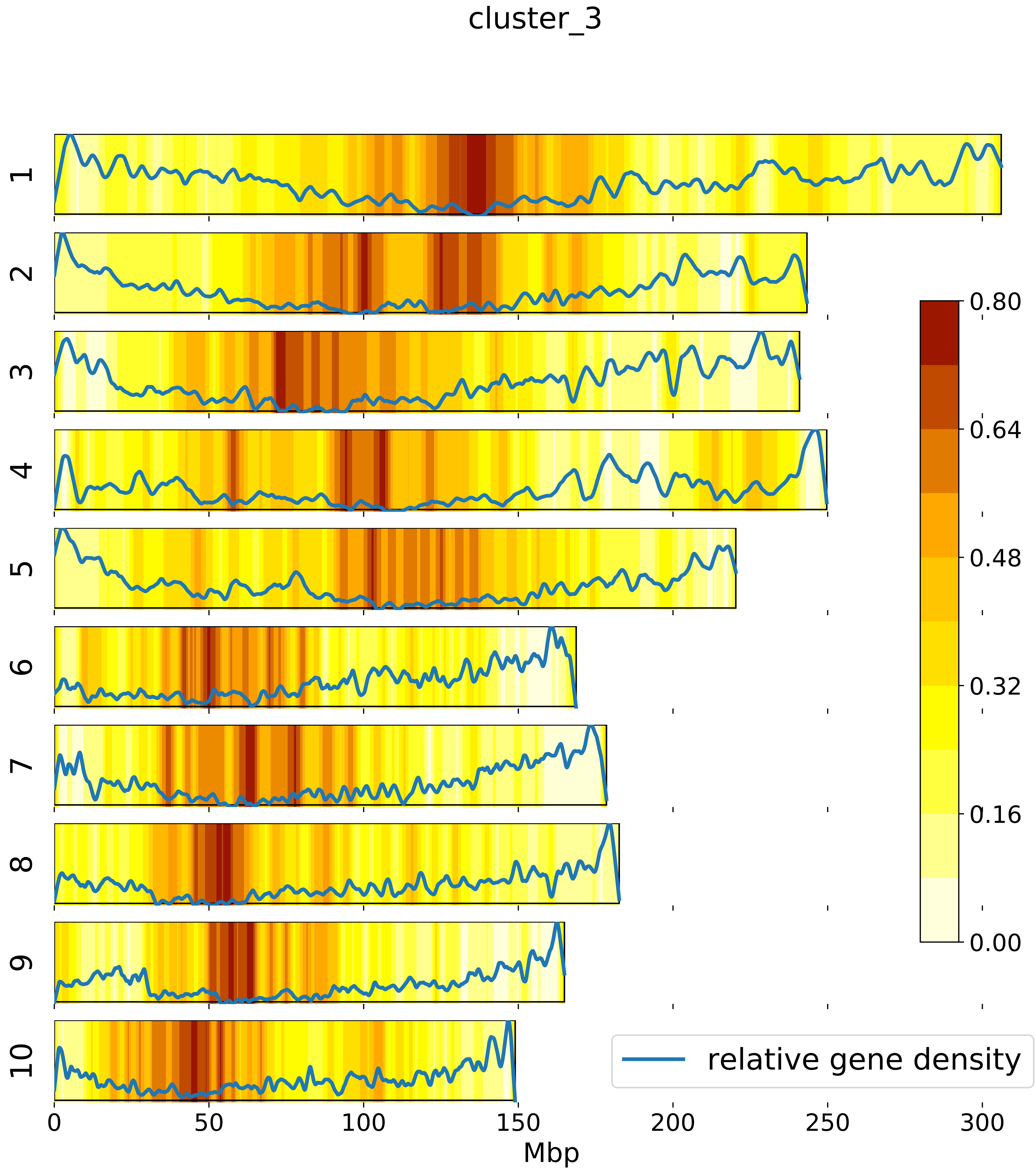
cluster_3
1
2
3
4
5
6
7
8
9
10
0
50
100
150
200
250
300
Mbp
0.80
0.64
0.48
0.32
0.16
0.00
relative gene density

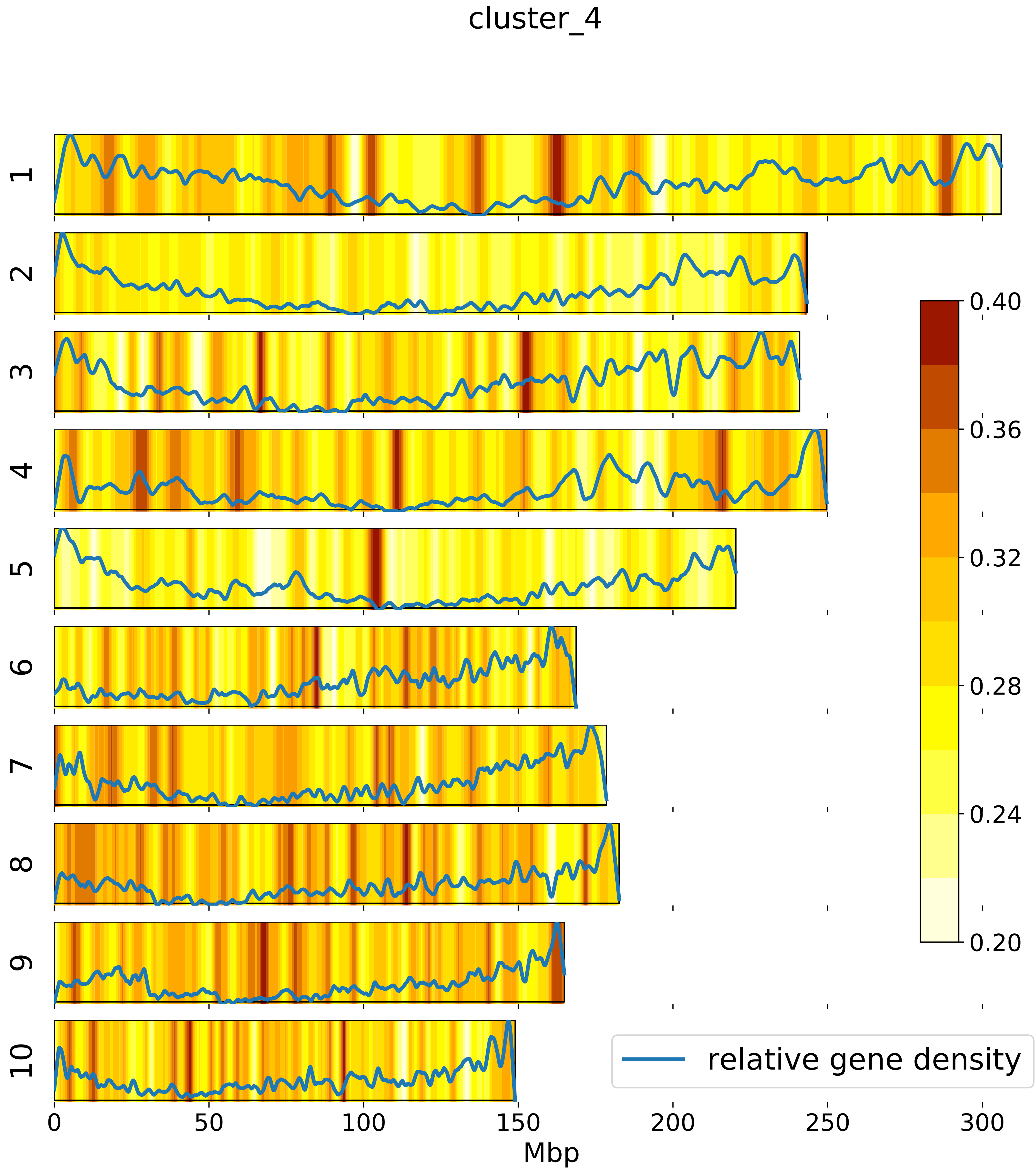

cluster_4
1
2
3
4
5
6
7
8
9
10
0
50
100
150
200
250
300
Mbp
0.40
0.36
0.32
0.28
0.24
0.20
relative gene density

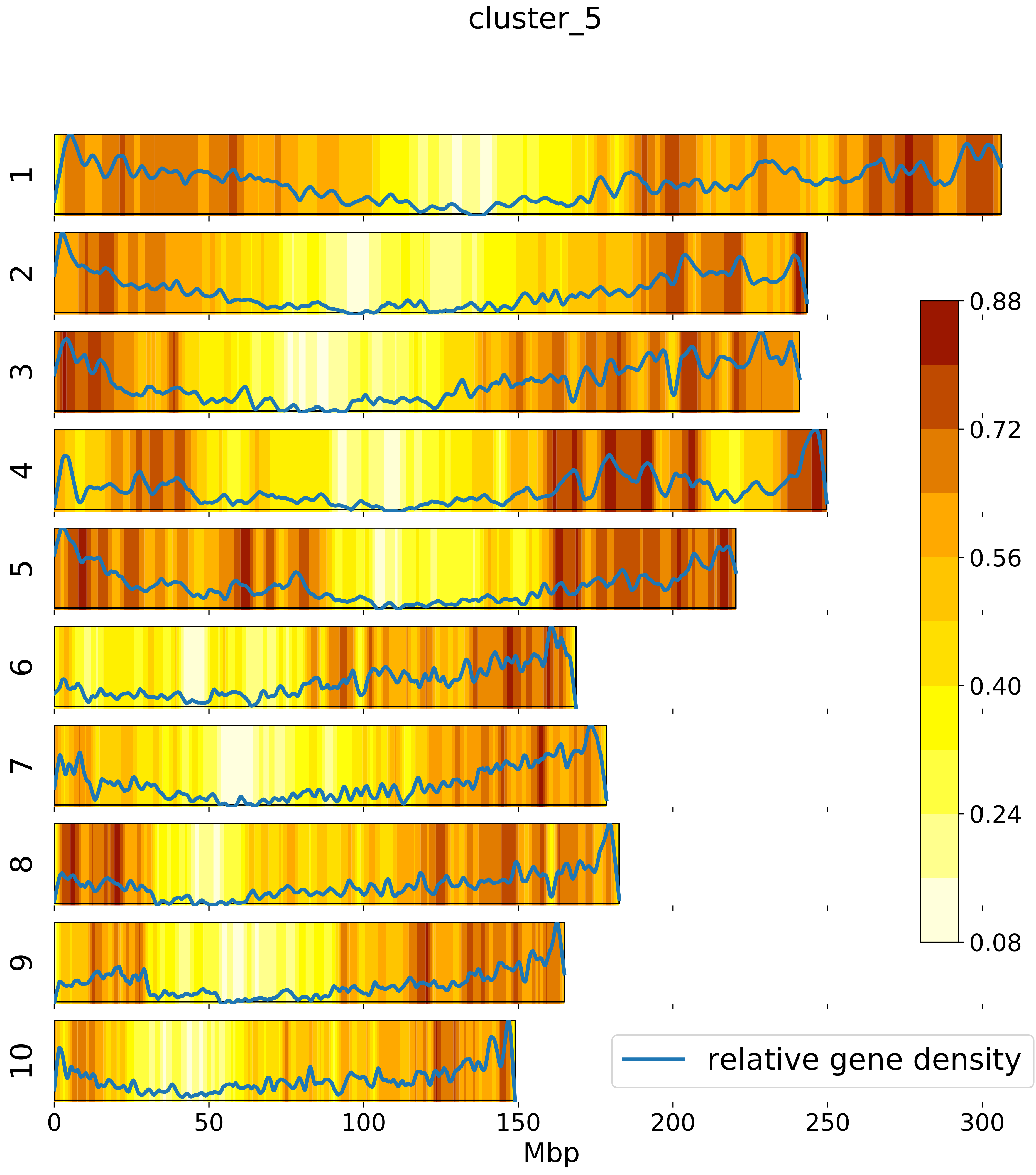

cluster_5
1
2
3
4
5
6
7
8
9
10
0
50
100
150
200
250
300
Mbp
0.88
0.72
0.56
0.40
0.24
0.08
relative gene density

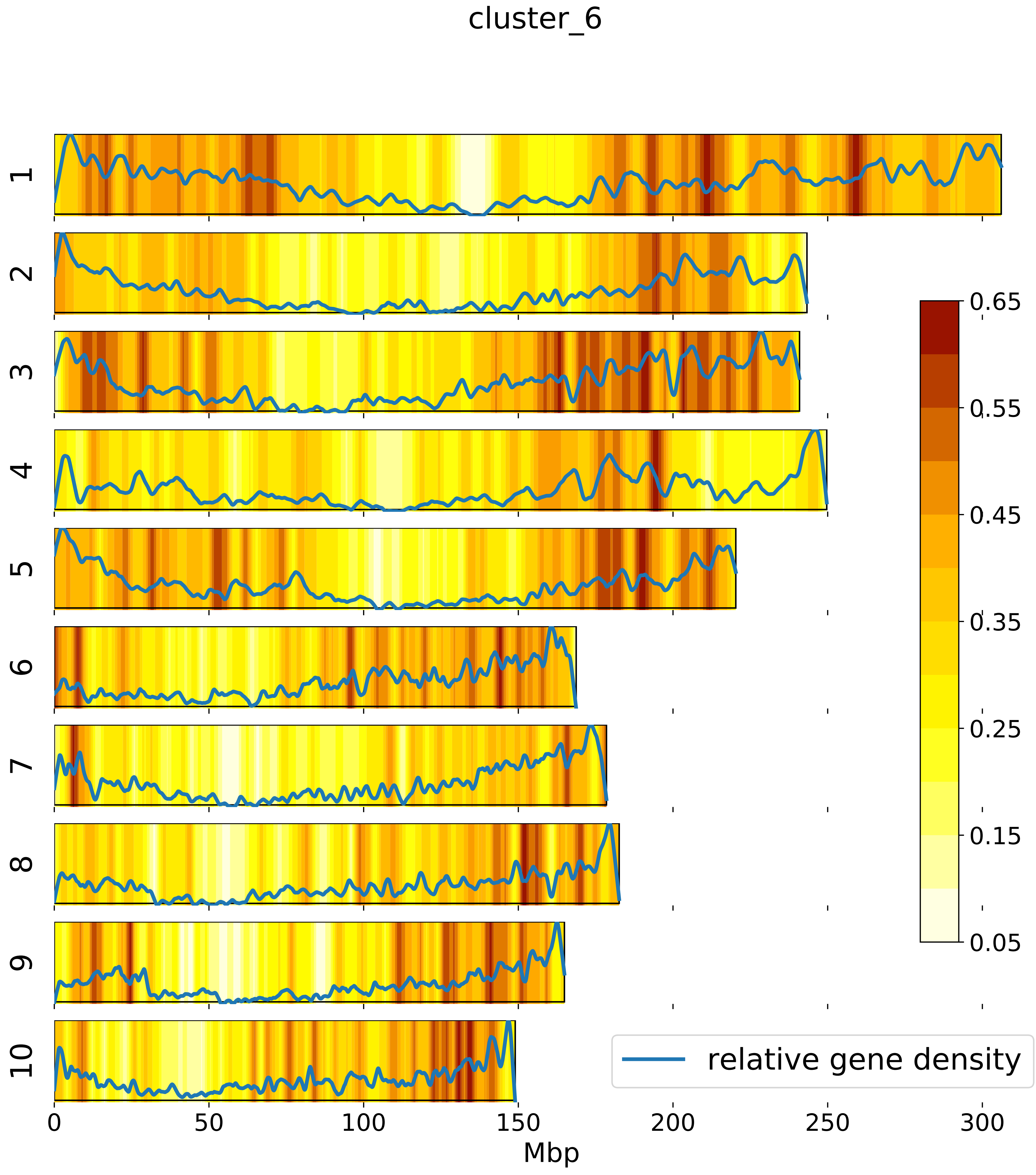
cluster_6
1
2
3
4
5
6
7
8
9
10
0
50
100
150
200
250
300
Mbp
0.65
0.55
0.45
0.35
0.25
0.15
0.05
relative gene density

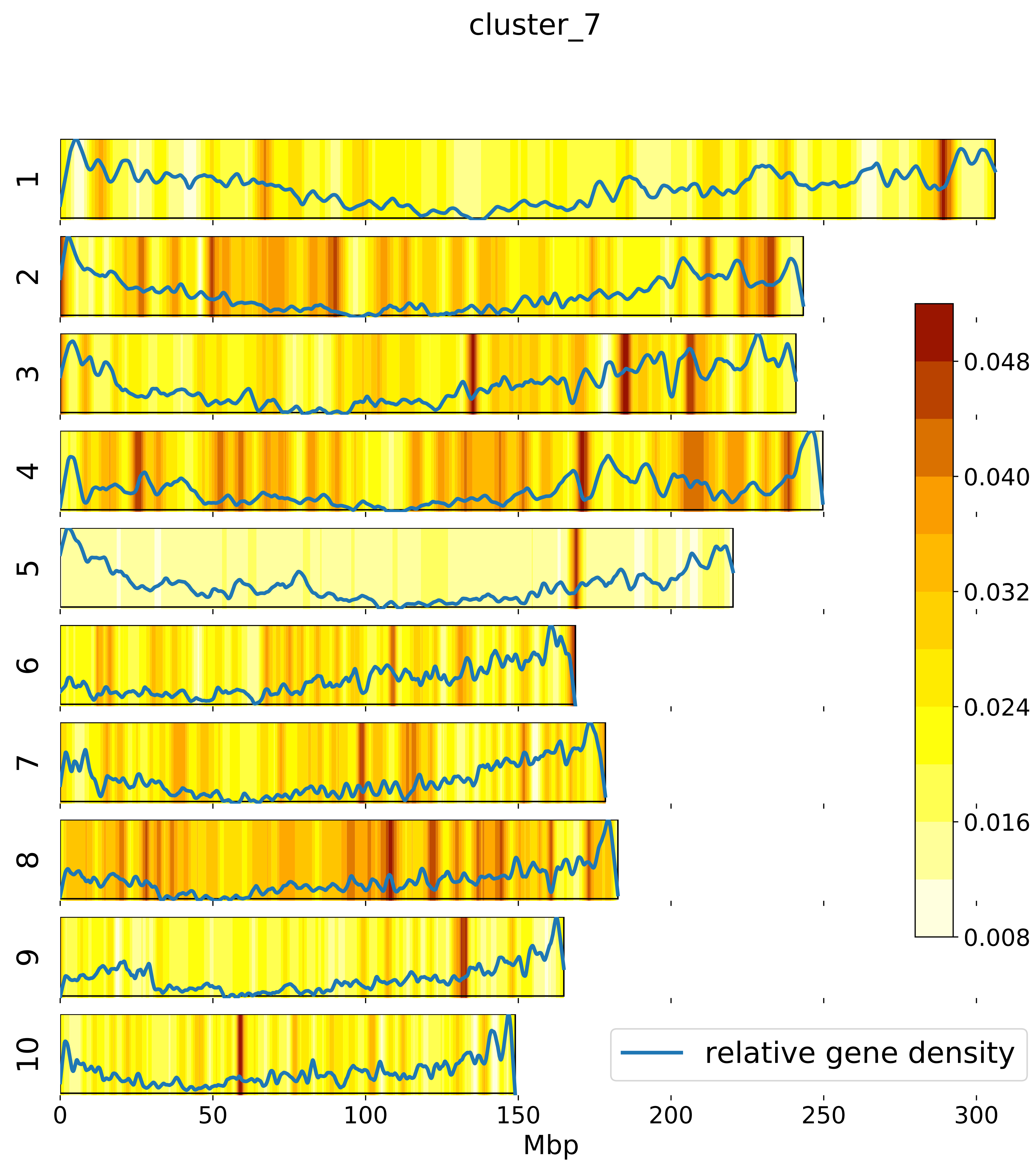
cluster_7
1
2
3
4
5
6
7
8
9
10
0
50
100
150
200
250
300
Mbp
0.048
0.040
0.032
0.024
0.016
0.008
relative gene density

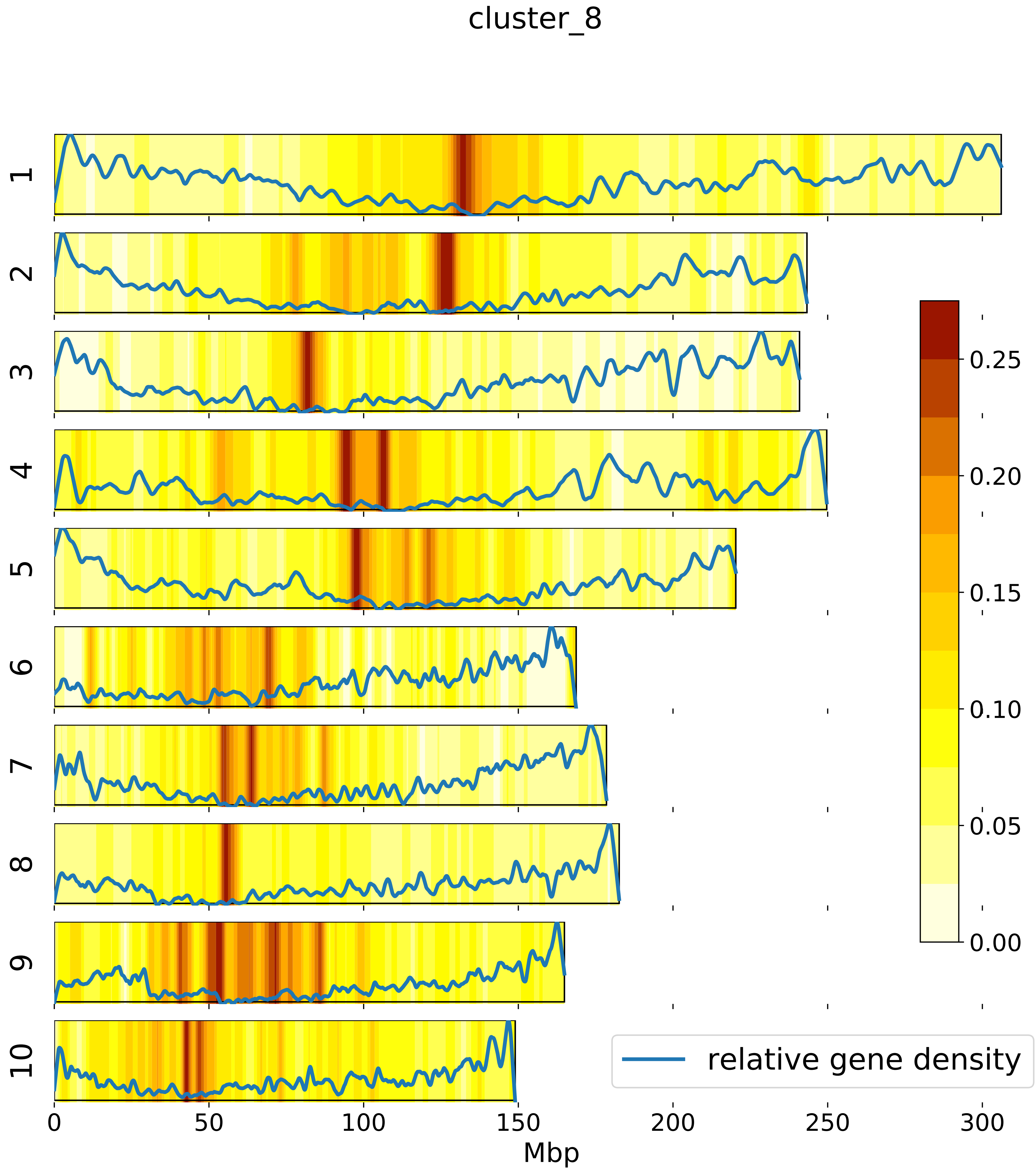

cluster_8
1
2
3
4
5
6
7
8
9
10
0
50
100
150
200
250
300
Mbp
0.00
0.05
0.10
0.15
0.20
0.25
relative gene density

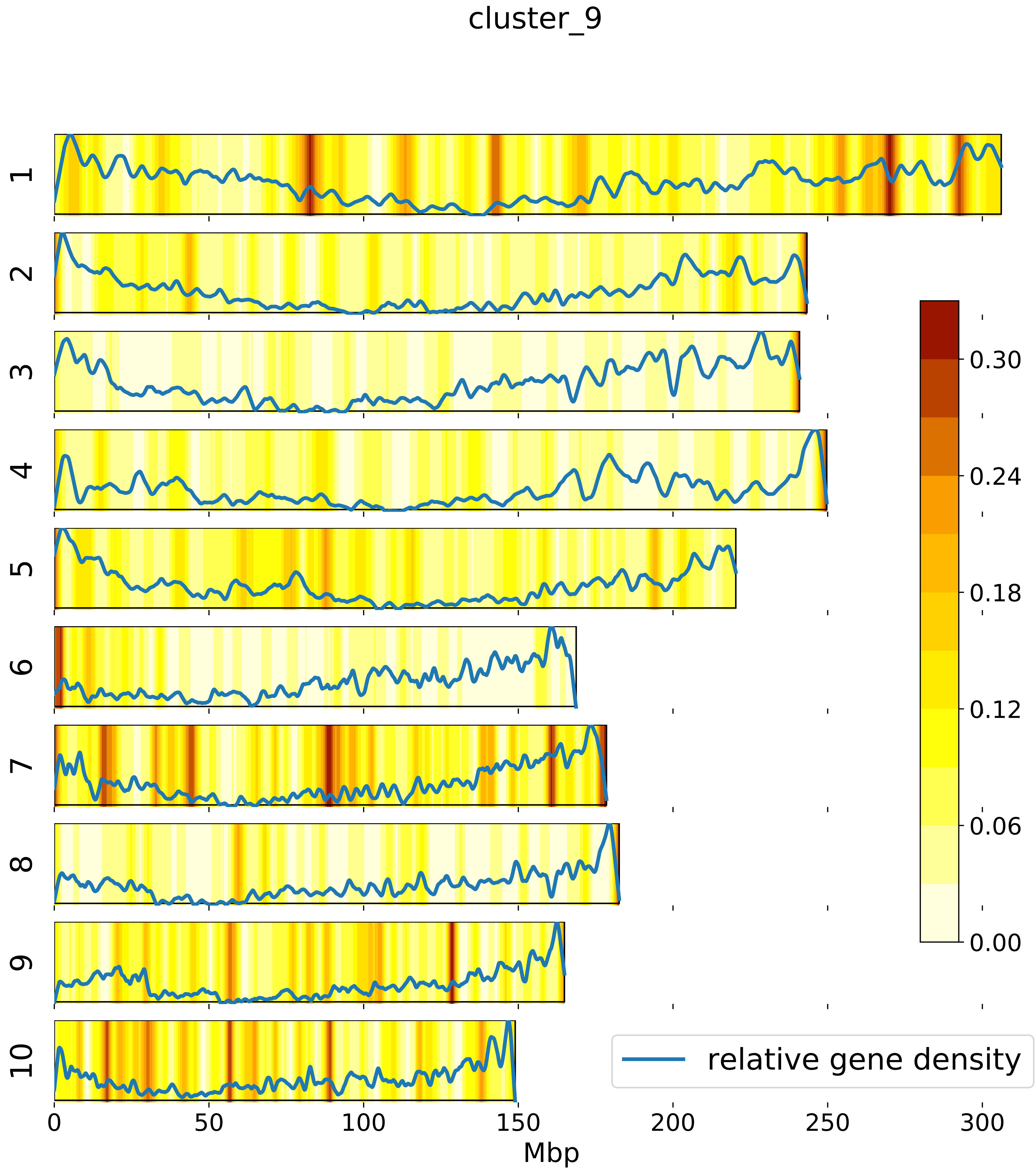

cluster_9
1
2
3
4
5
6
7
8
9
10
0
50
100
150
200
250
300
Mbp
0.30
0.24
0.18
0.12
0.06
0.00
relative gene density

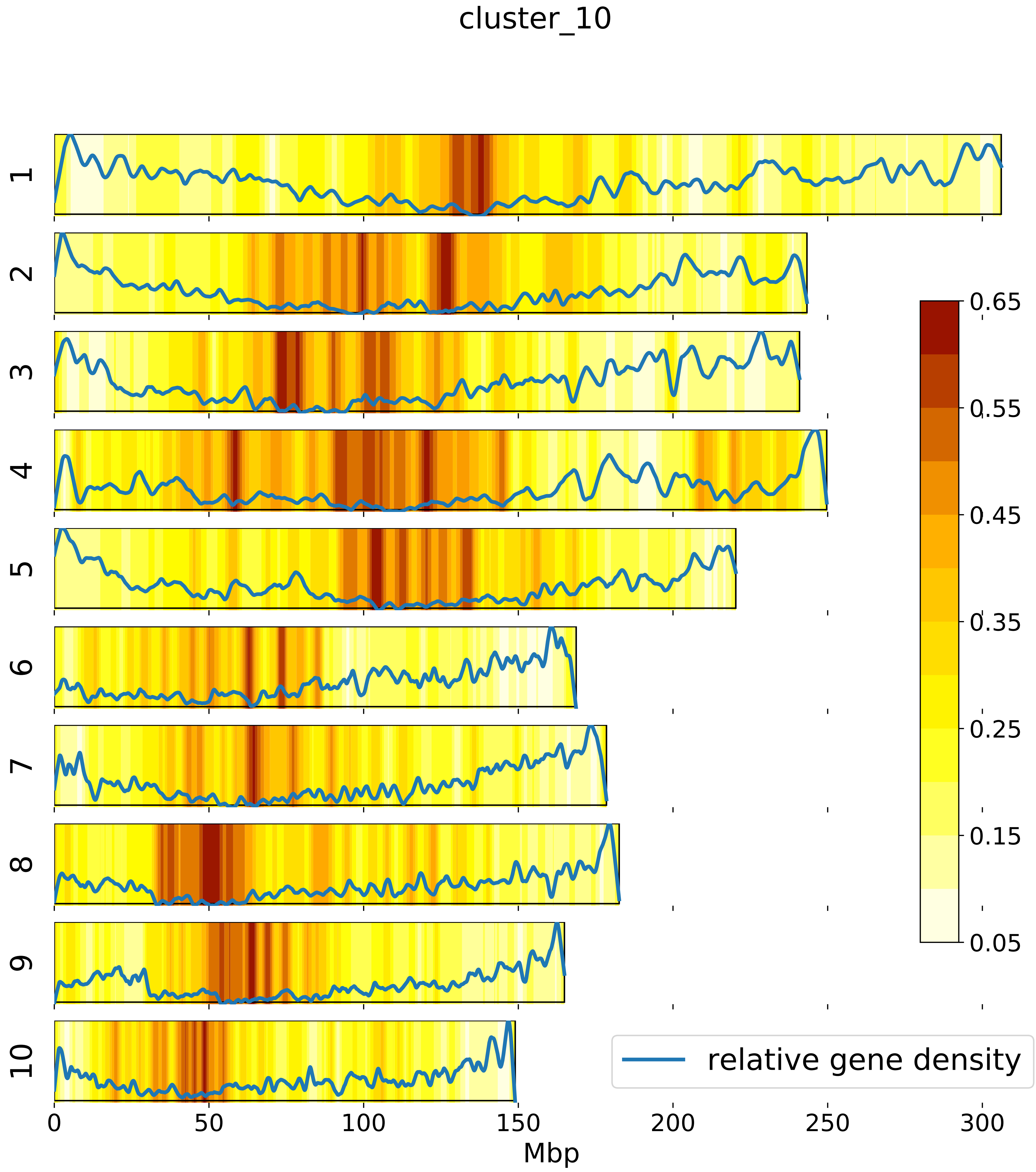
cluster_10
1
2
3
4
5
6
7
8
9
10
0
50
100
150
200
250
300
Mbp
0.65
0.55
0.45
0.35
0.25
0.15
0.05
relative gene density

cluster_11

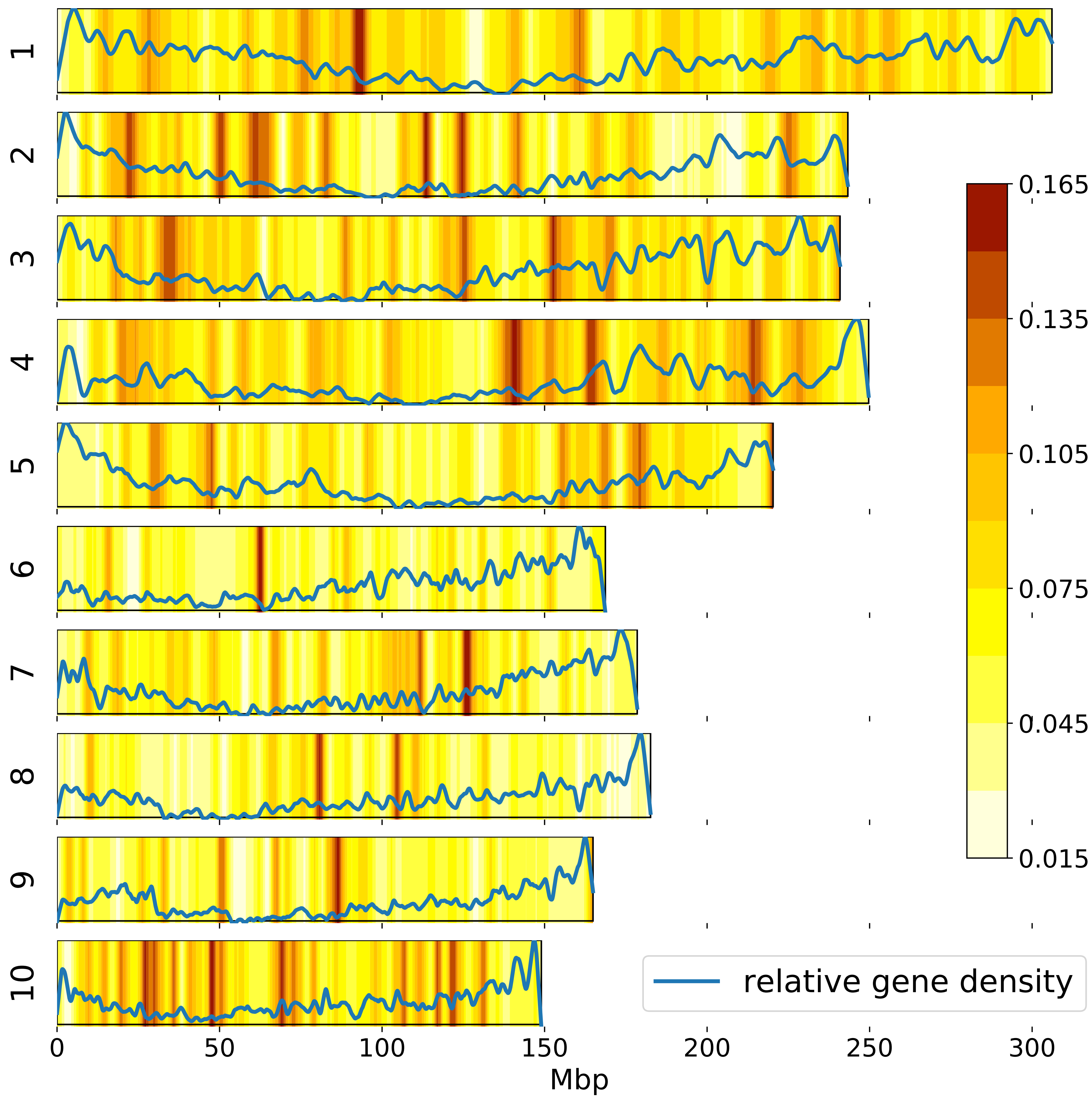

1
2
3
4
5
6
7
8
9
10
0
50
100
150
200
250
300
Mbp
0.165
0.135
0.105
0.075
0.045
0.015
relative gene density

cluster_12

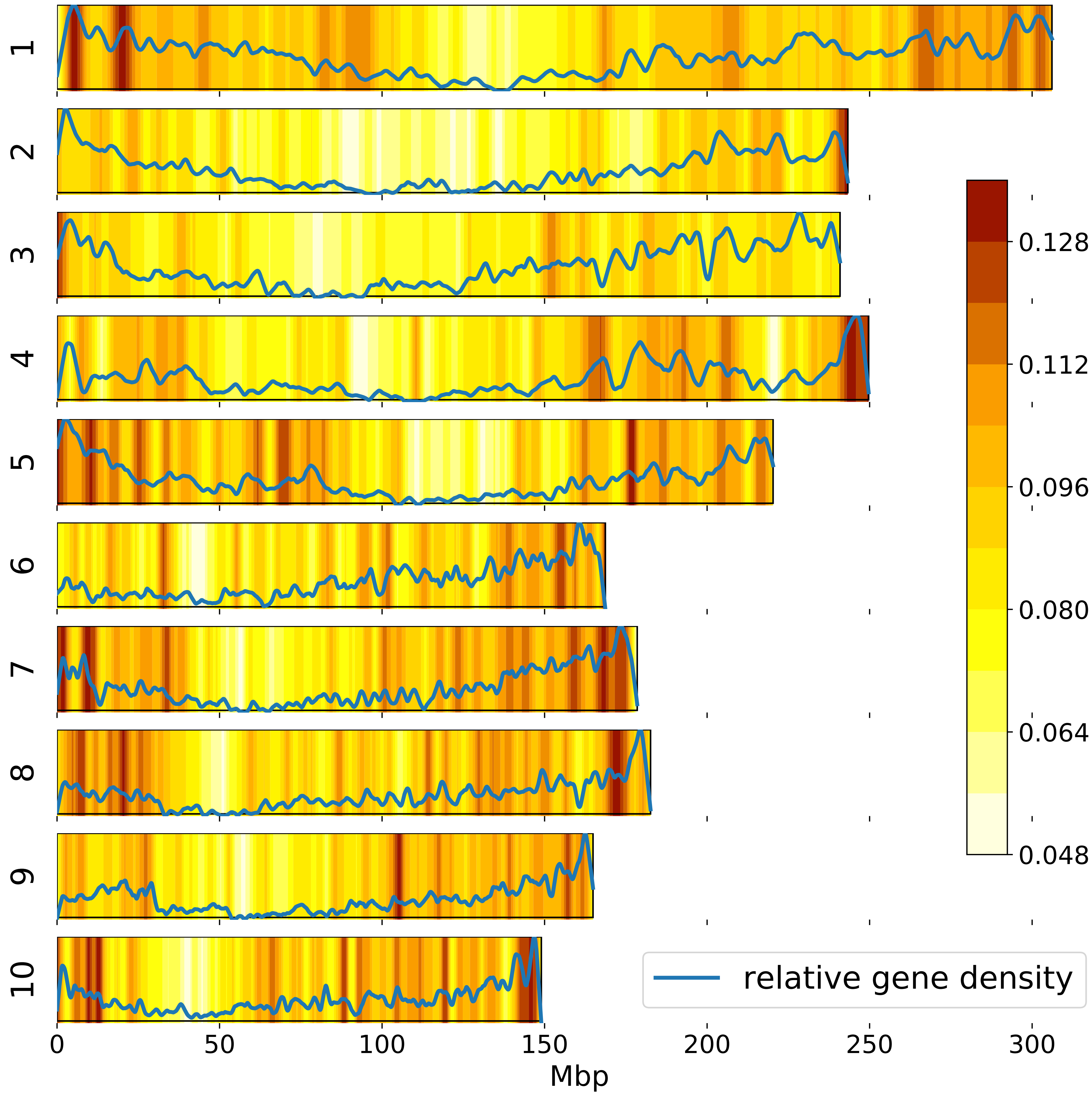

1
2
3
4
5
6
7
8
9
10
0.128
0.112
0.096
0.080
0.064
0.048
relative gene density
0
50
100
150
200
250
300
Mbp

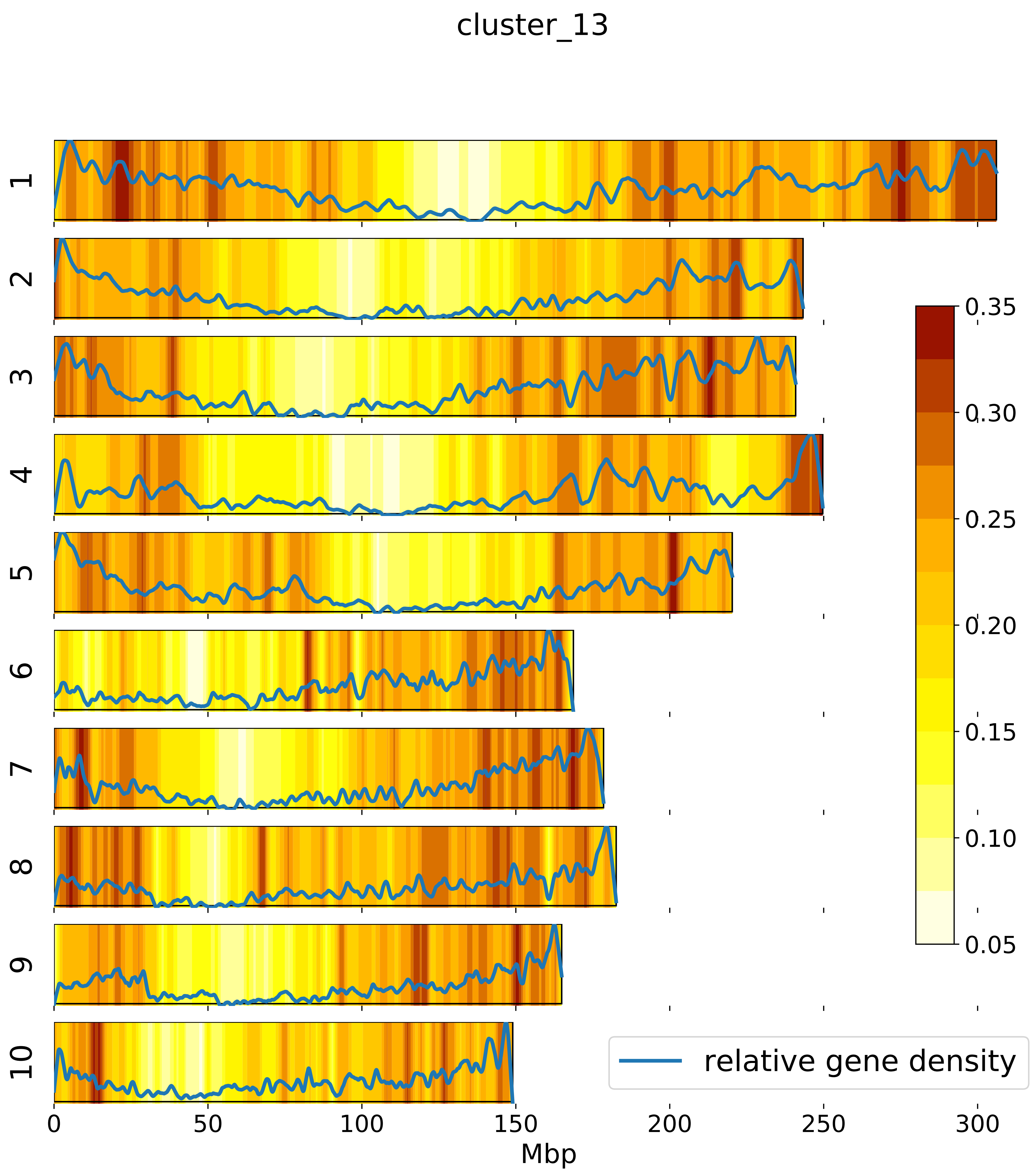

cluster_13
1
2
3
4
5
6
7
8
9
10
0
50
100
150
200
250
300
Mbp
0.35
0.30
0.25
0.20
0.15
0.10
0.05
relative gene density

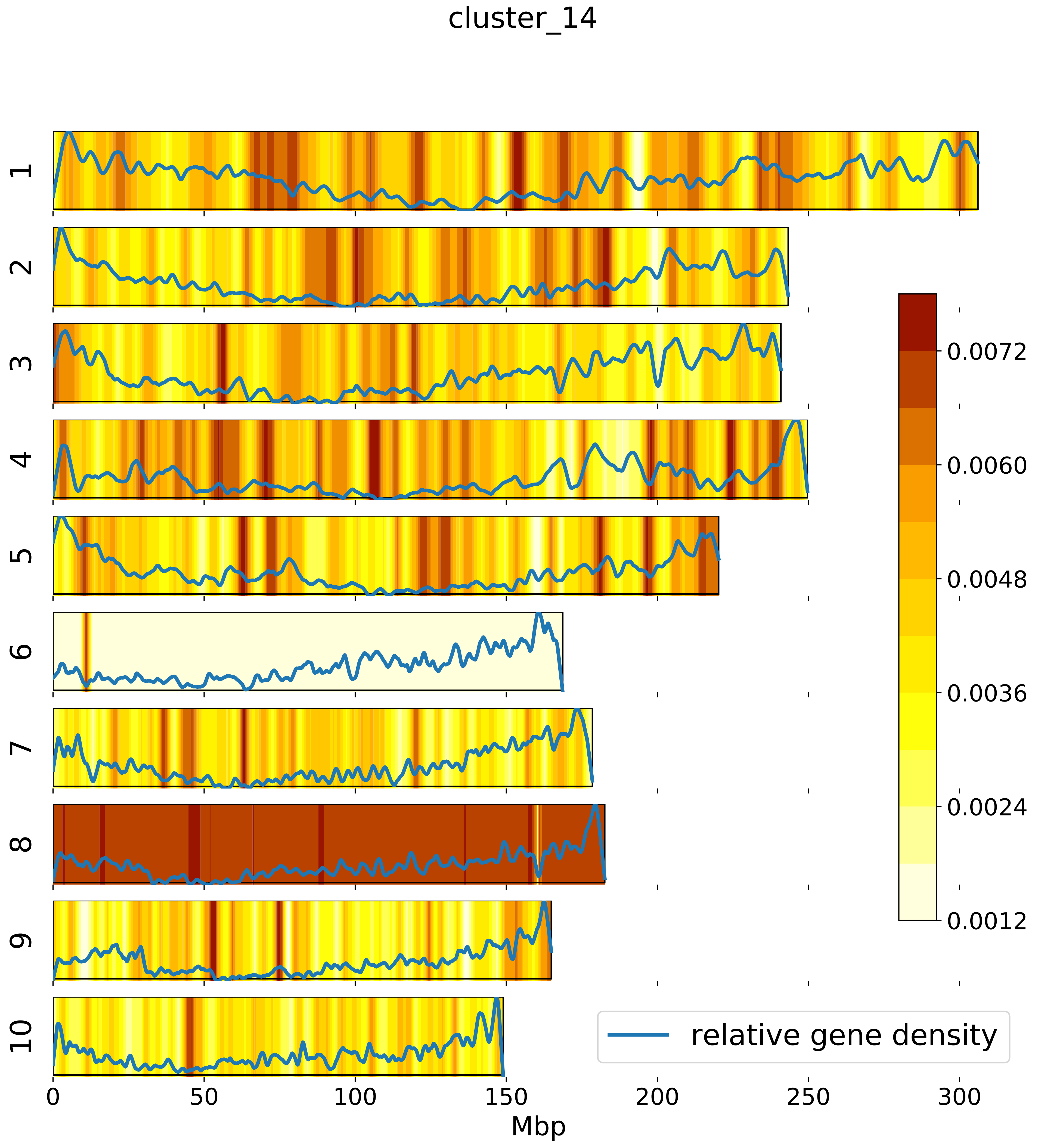

cluster_14
1
2
3
4
5
6
7
8
9
10
0
50
100
150
200
250
300
Mbp
0.0072
0.0060
0.0048
0.0036
0.0024
0.0012
relative gene density

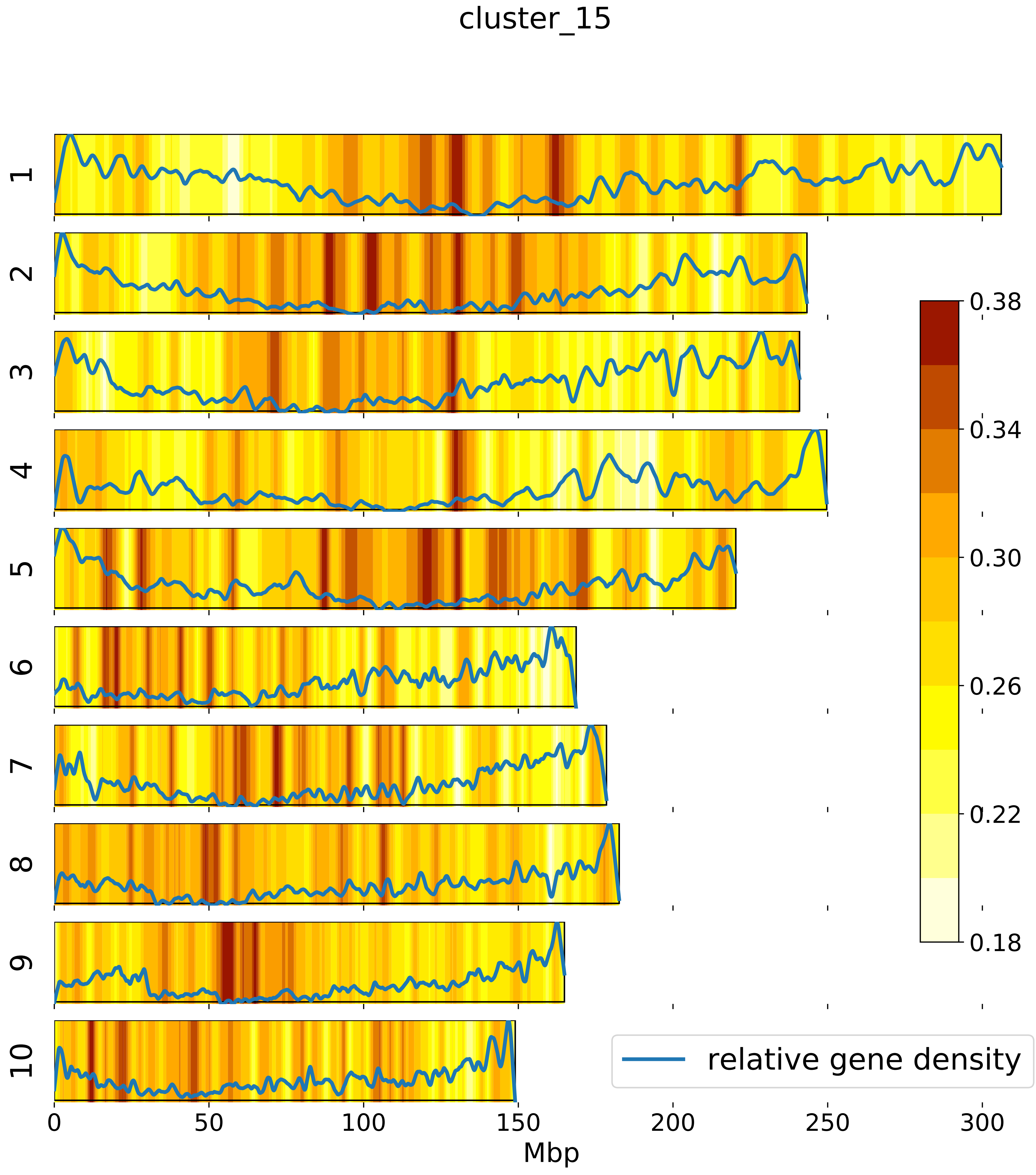
cluster_15
1
2
3
4
5
6
7
8
9
10
0
50
100
150
200
250
300
Mbp
0.38
0.34
0.30
0.26
0.22
0.18
relative gene density

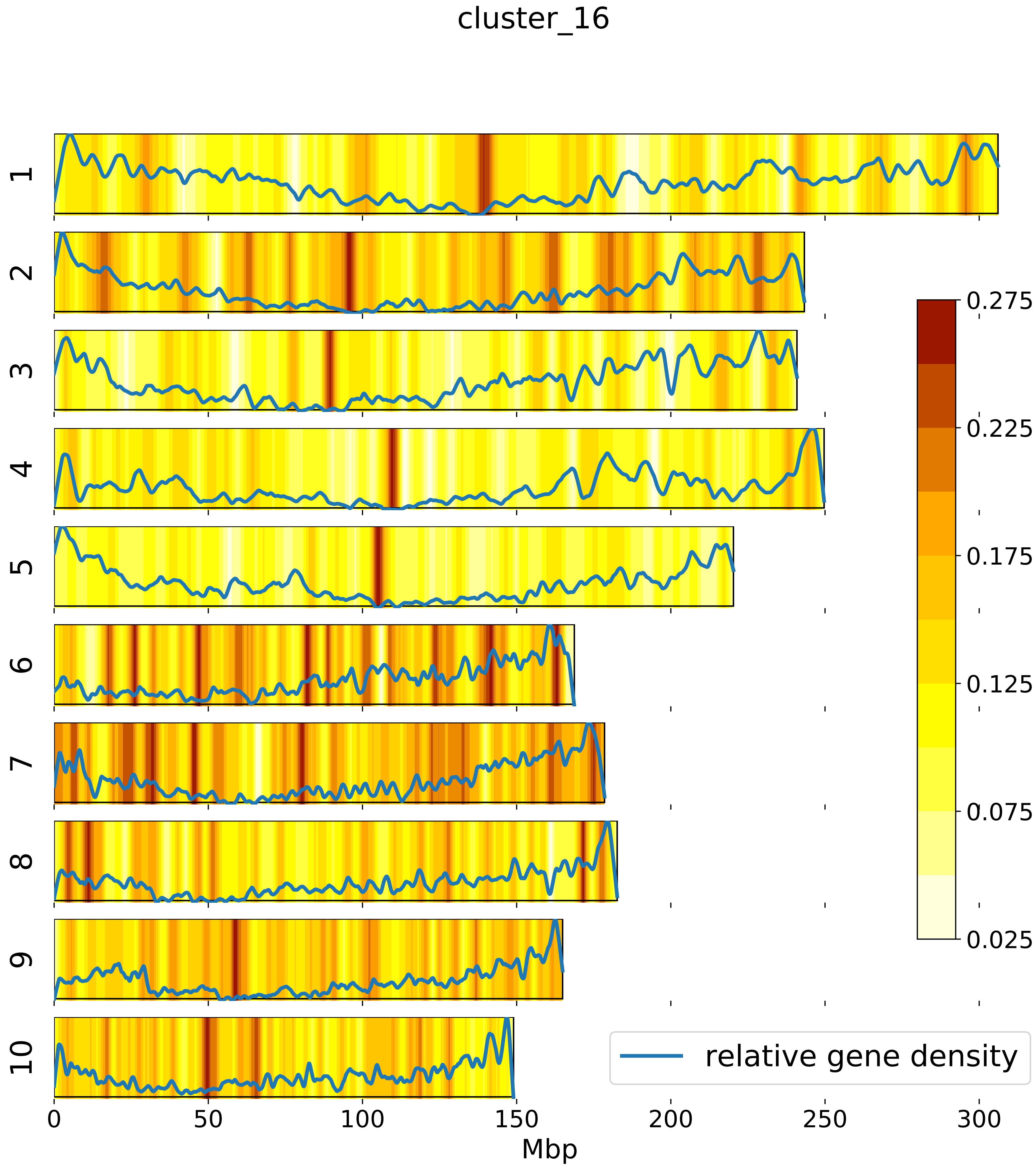

cluster_16
1
2
3
4
5
6
7
8
9
10
0
50
100
150
200
250
300
Mbp
0.275
0.225
0.175
0.125
0.075
0.025
relative gene density

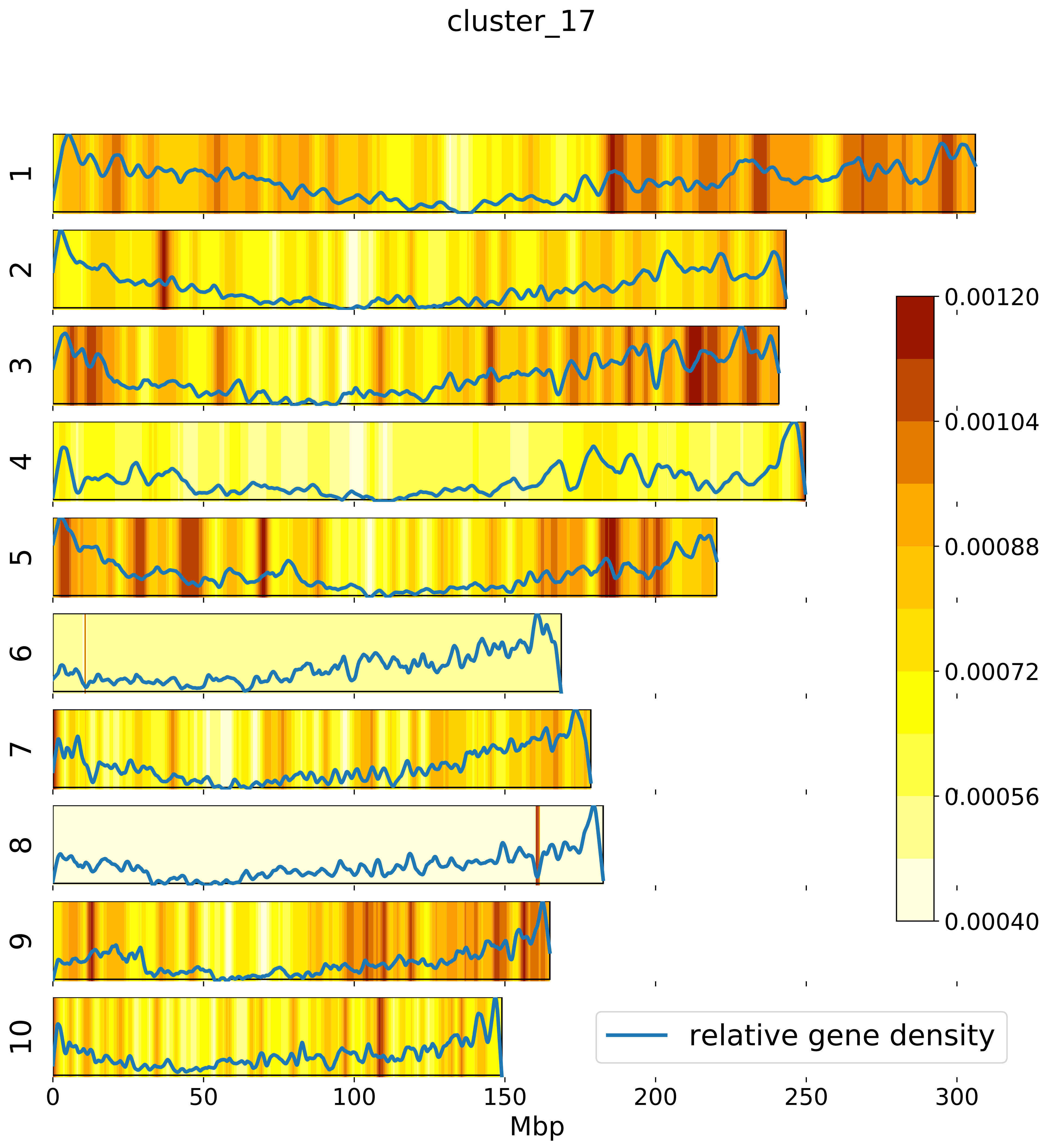
cluster_17
1
2
3
4
5
6
7
8
9
10
0
50
100
150
200
250
300
Mbp
0.00120
0.00104
0.00088
0.00072
0.00056
0.00040
relative gene density

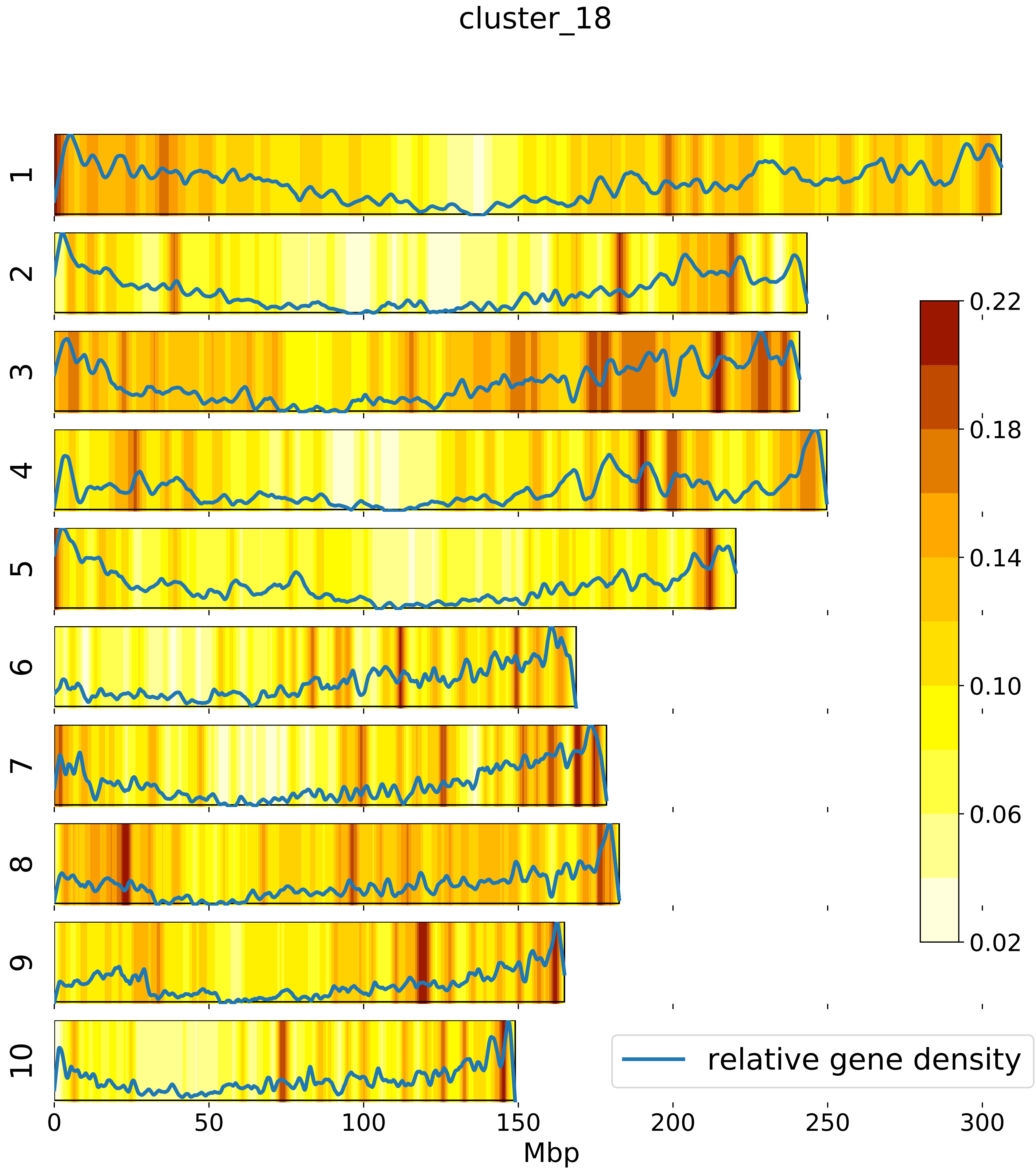
cluster_18
1
2
3
4
5
6
7
8
9
10
0
50
100
150
200
250
300
Mbp
0.22
0.18
0.14
0.10
0.06
0.02
relative gene density

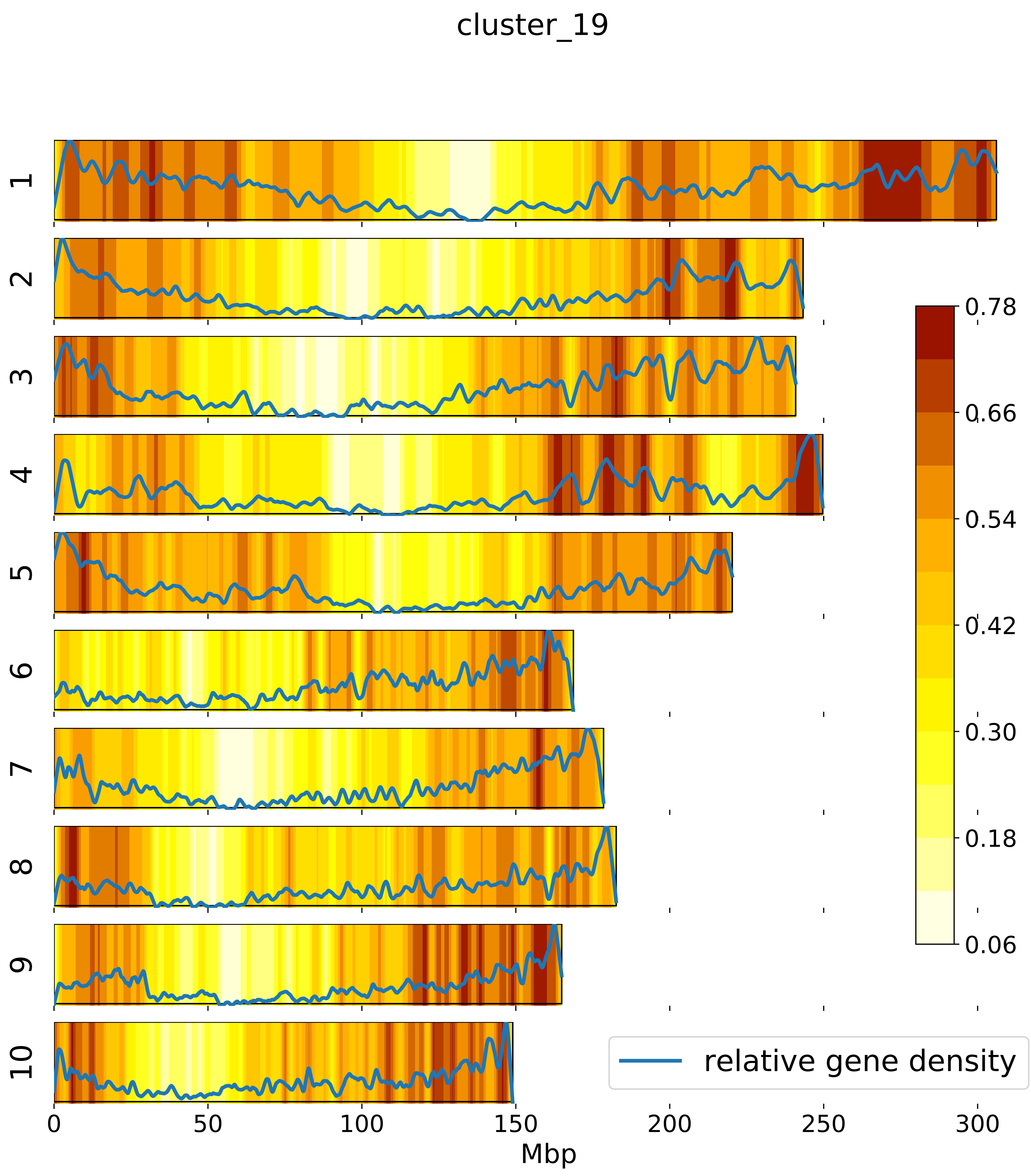
cluster_19
1
2
3
4
5
6
7
8
9
10
0
50
100
150
200
250
300
Mbp
0.78
0.66
0.54
0.42
0.30
0.18
0.06
relative gene density

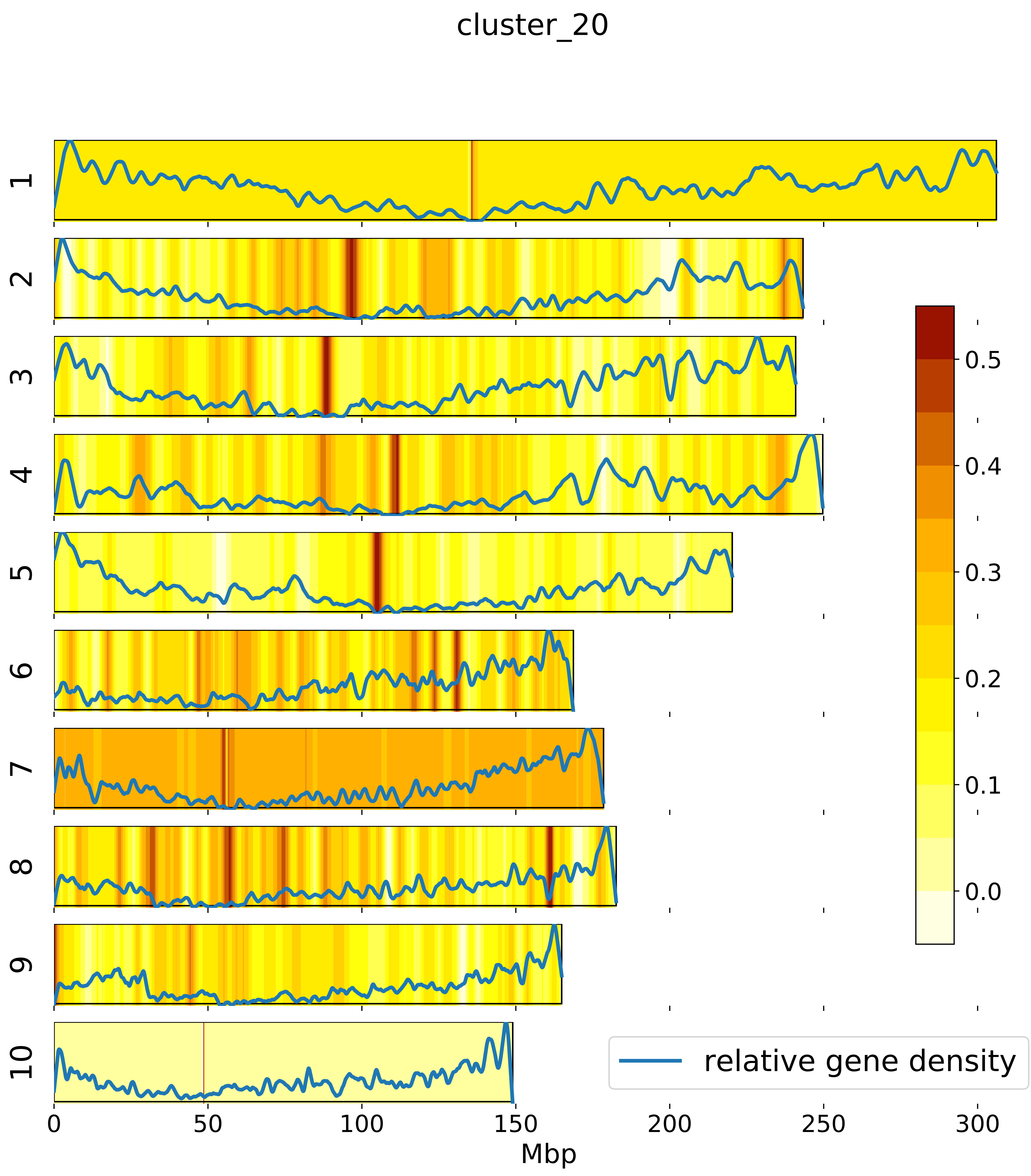
cluster_20
1
2
3
4
5
6
7
8
9
10
0
50
100
150
200
250
300
Mbp
0.0
0.1
0.2
0.3
0.4
0.5
relative gene density

cluster_21

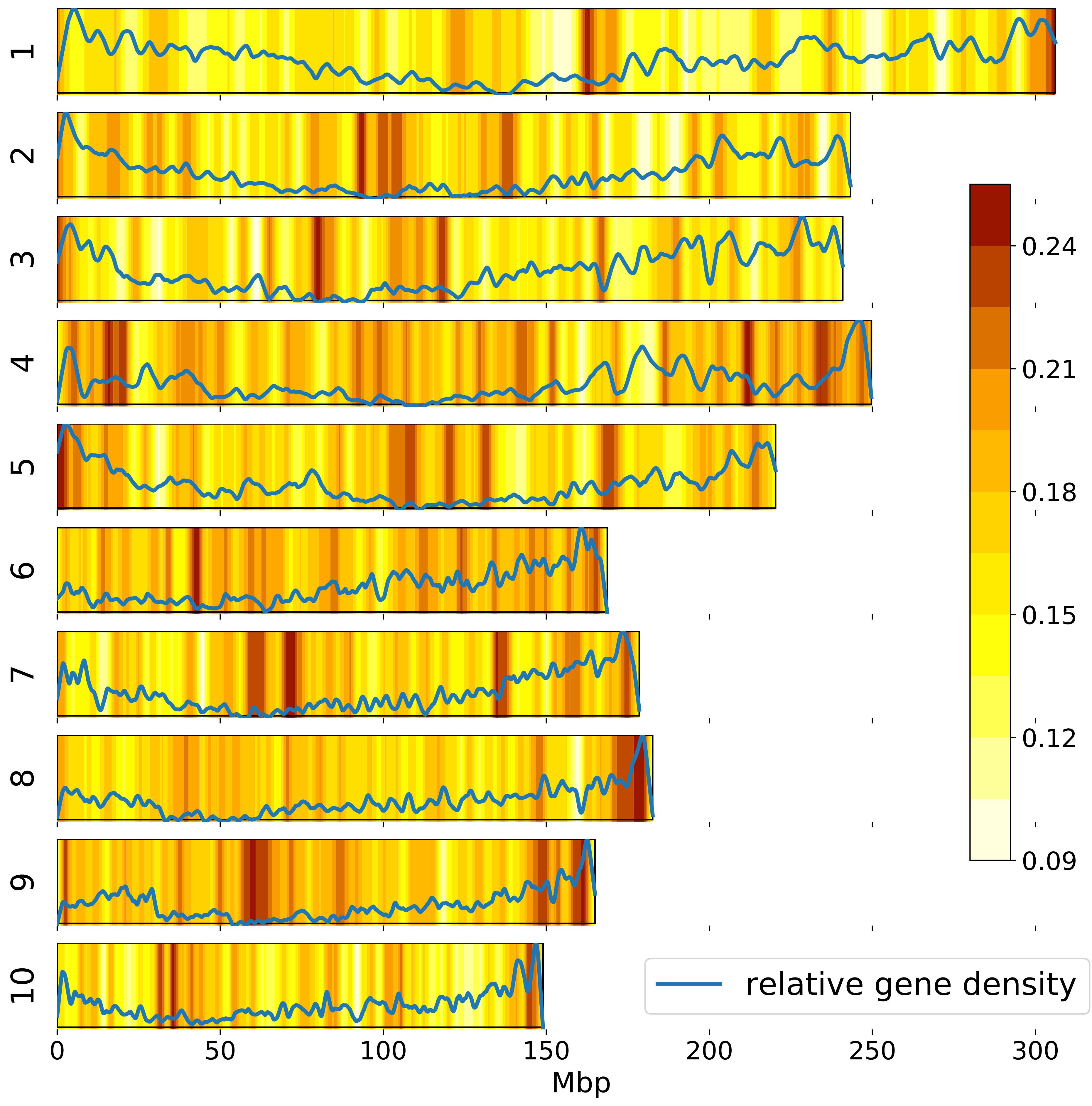

1
2
3
4
5
6
7
8
9
10
0
50
100
150
200
250
300
Mbp
0.24
0.21
0.18
0.15
0.12
0.09
relative gene density

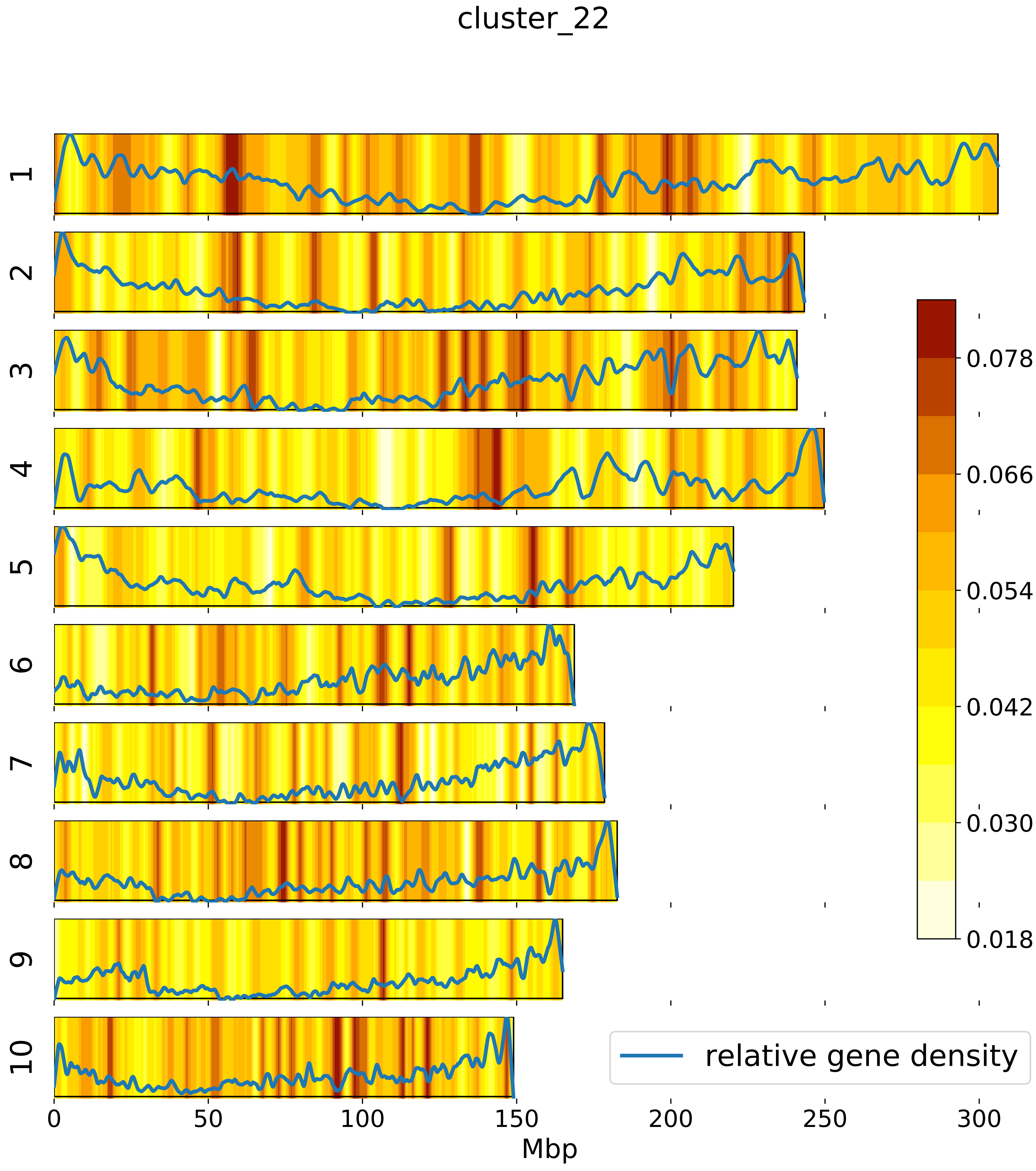
cluster_22
1
2
3
4
5
6
7
8
9
10
0
50
100
150
200
250
300
Mbp
0.078
0.066
0.054
0.042
0.030
0.018
relative gene density

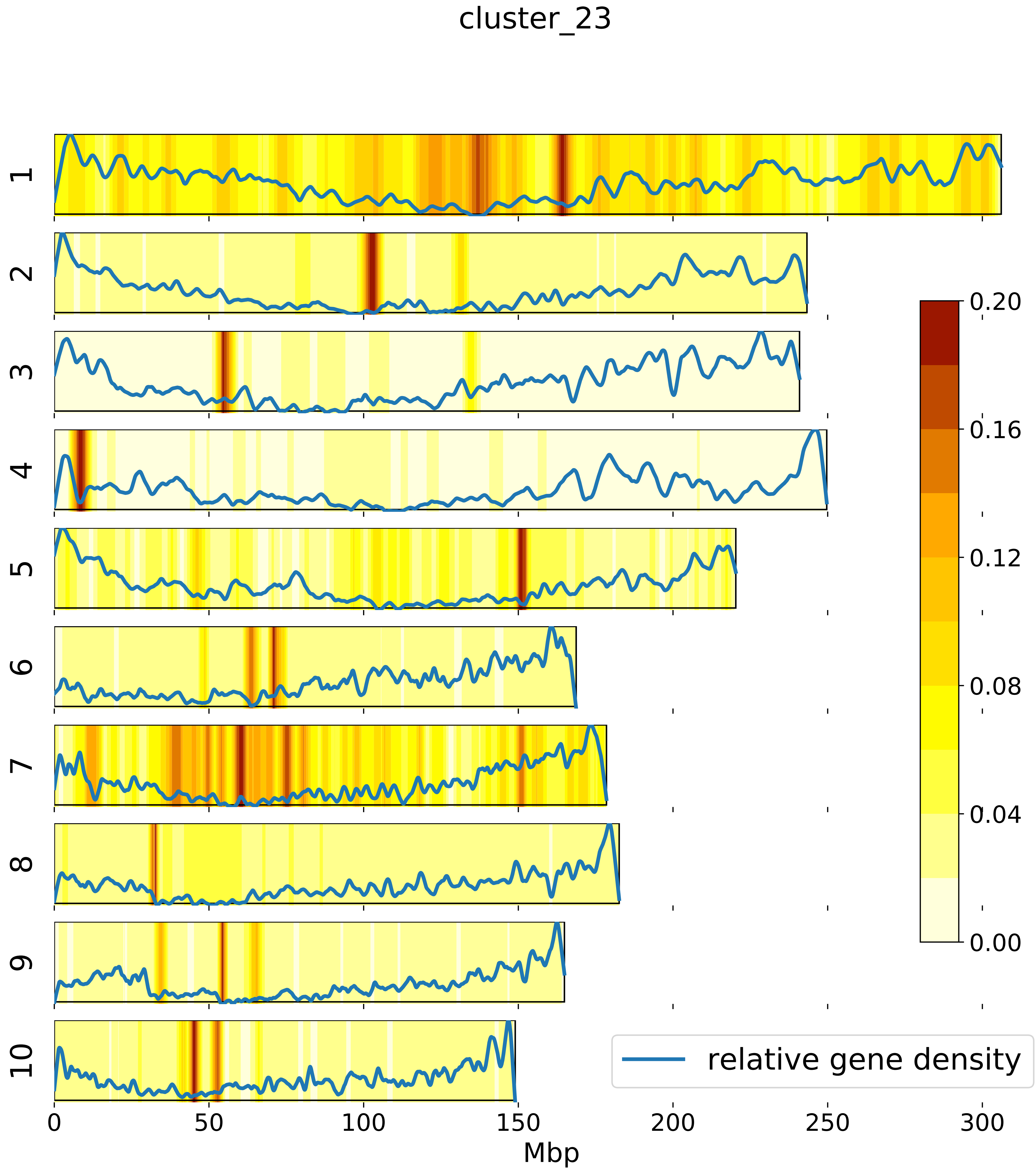
cluster_23
1
2
3
4
5
6
7
8
9
10
0.20
0.16
0.12
0.08
0.04
0.00
relative gene density
0
50
100
150
200
250
300
Mbp

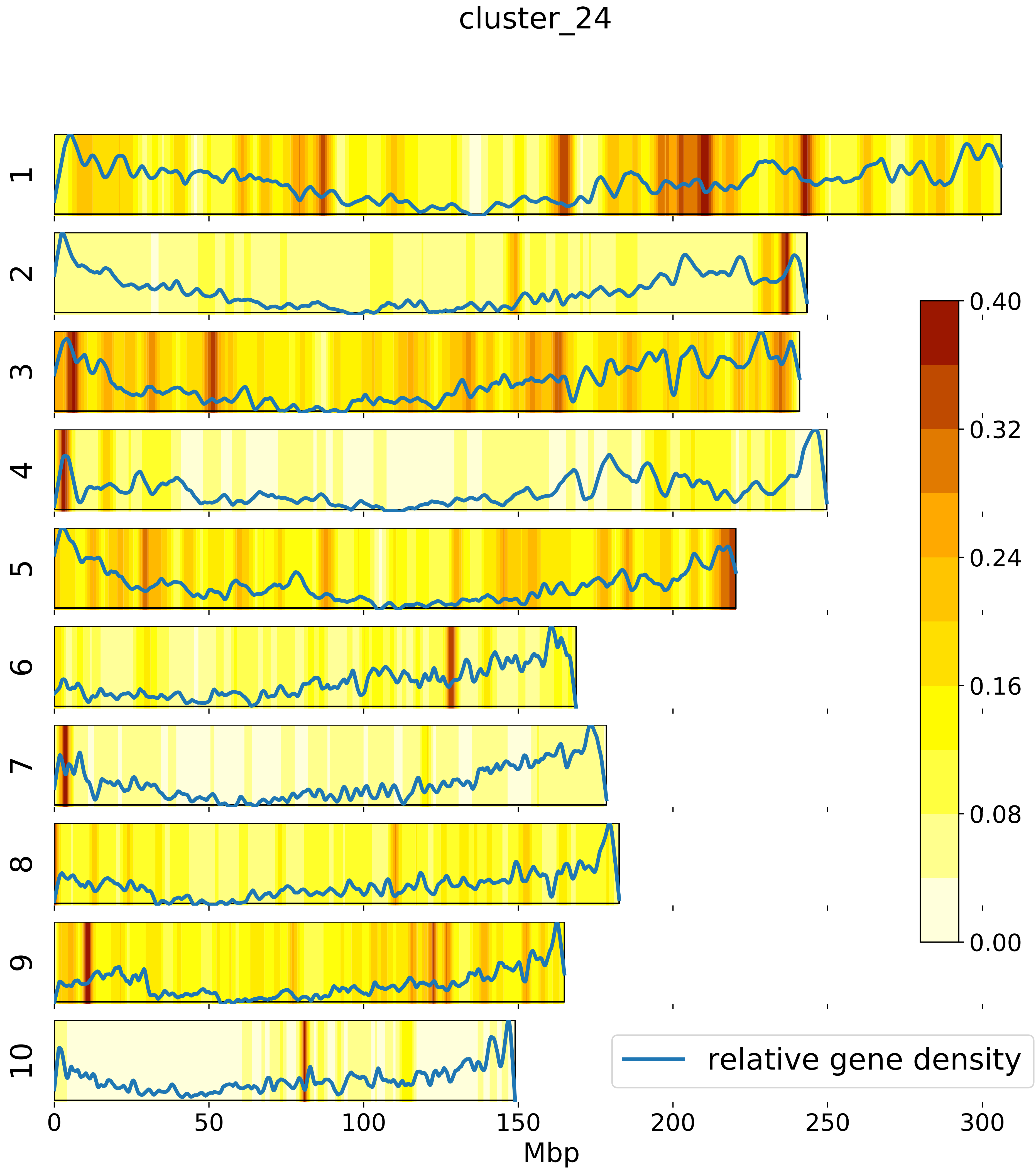

cluster_24
1
2
3
4
5
6
7
8
9
10
0
50
100
150
200
250
300
Mbp
0.40
0.32
0.24
0.16
0.08
0.00
relative gene density
