# Supplementary material for: Repetitive DNA content in the maize genome is uncoupled from population stratification at SNP loci
Source: BMC Genomics. 2020 Jan 30;21:98. doi: 10.1186/s12864-020-6517-0 (PMC6993463; doi:10.1186/s12864-020-6517-0)

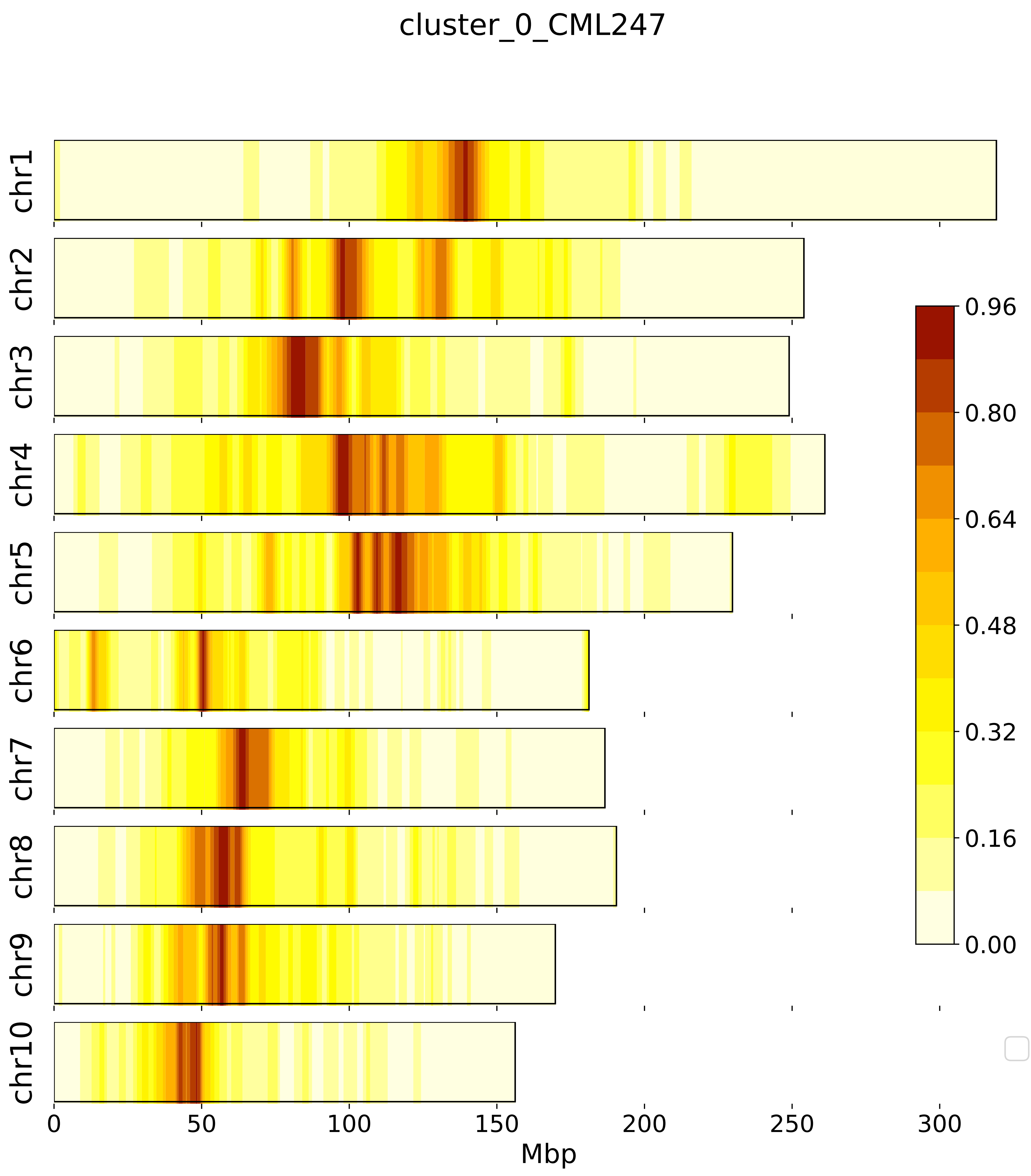

cluster_0_CML247
chr1
chr2
chr3
chr4
chr5
chr6
chr7
chr8
chr9
chr10
0
50
100
150
200
250
300
Mbp
0.96
0.80
0.64
0.48
0.32
0.16
0.00

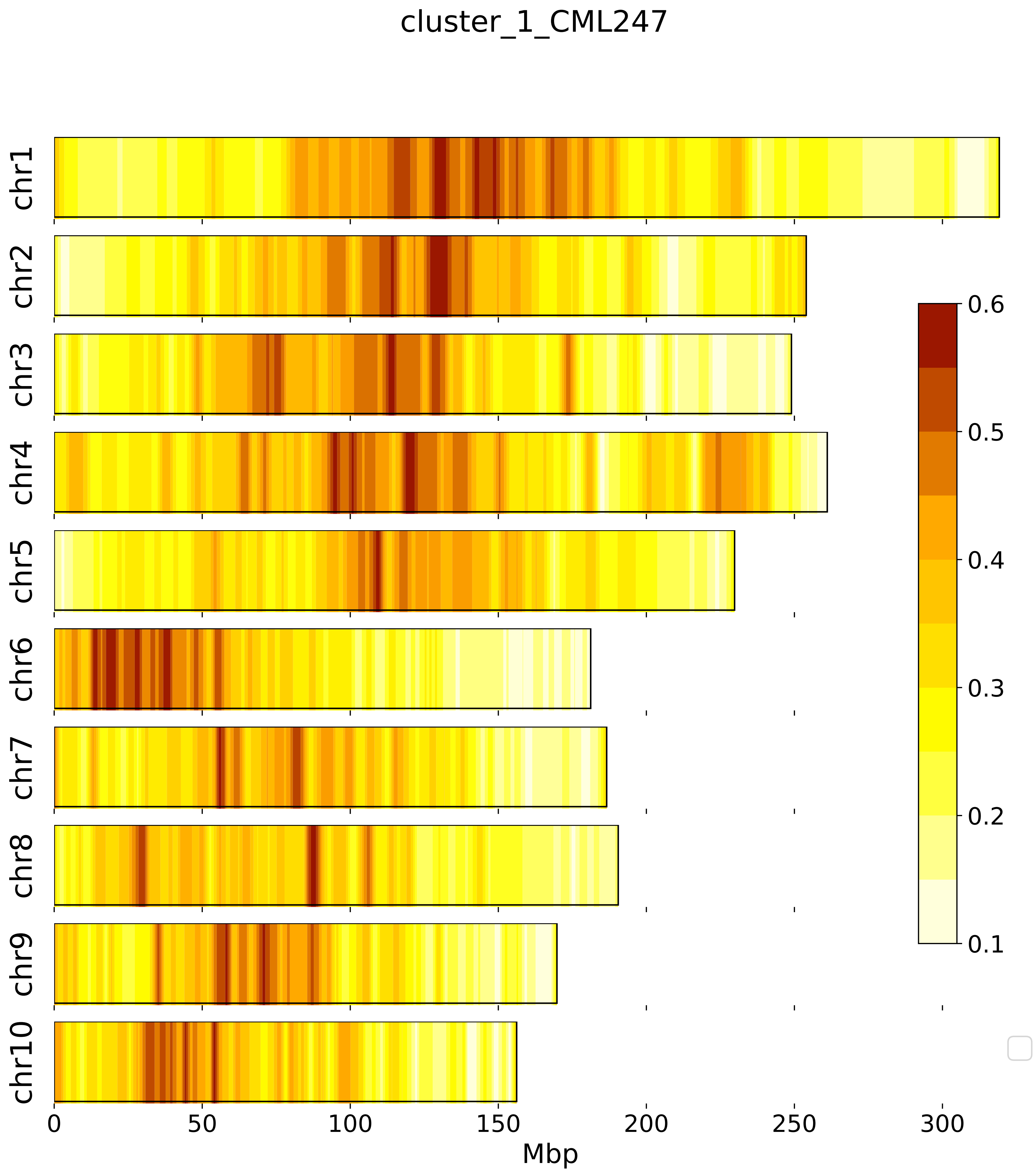

cluster_1_CML247
chr1
chr2
chr3
chr4
chr5
chr6
chr7
chr8
chr9
chr10
0
50
100
150
200
250
300
Mbp
0.6
0.5
0.4
0.3
0.2
0.1

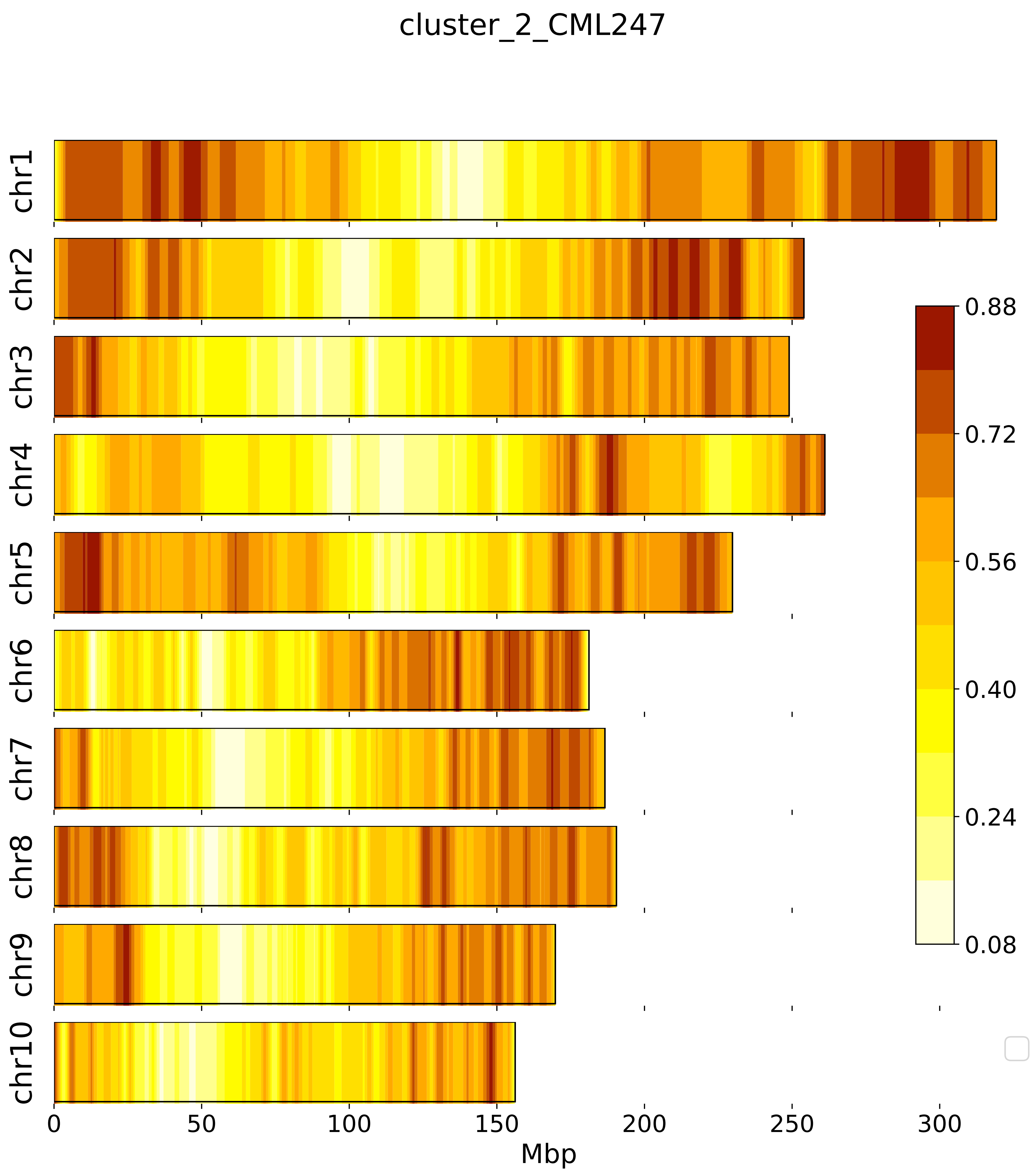

cluster_2_CML247
chr1
chr2
chr3
chr4
chr5
chr6
chr7
chr8
chr9
chr10
0
50
100
150
200
250
300
Mbp
0.88
0.72
0.56
0.40
0.24
0.08

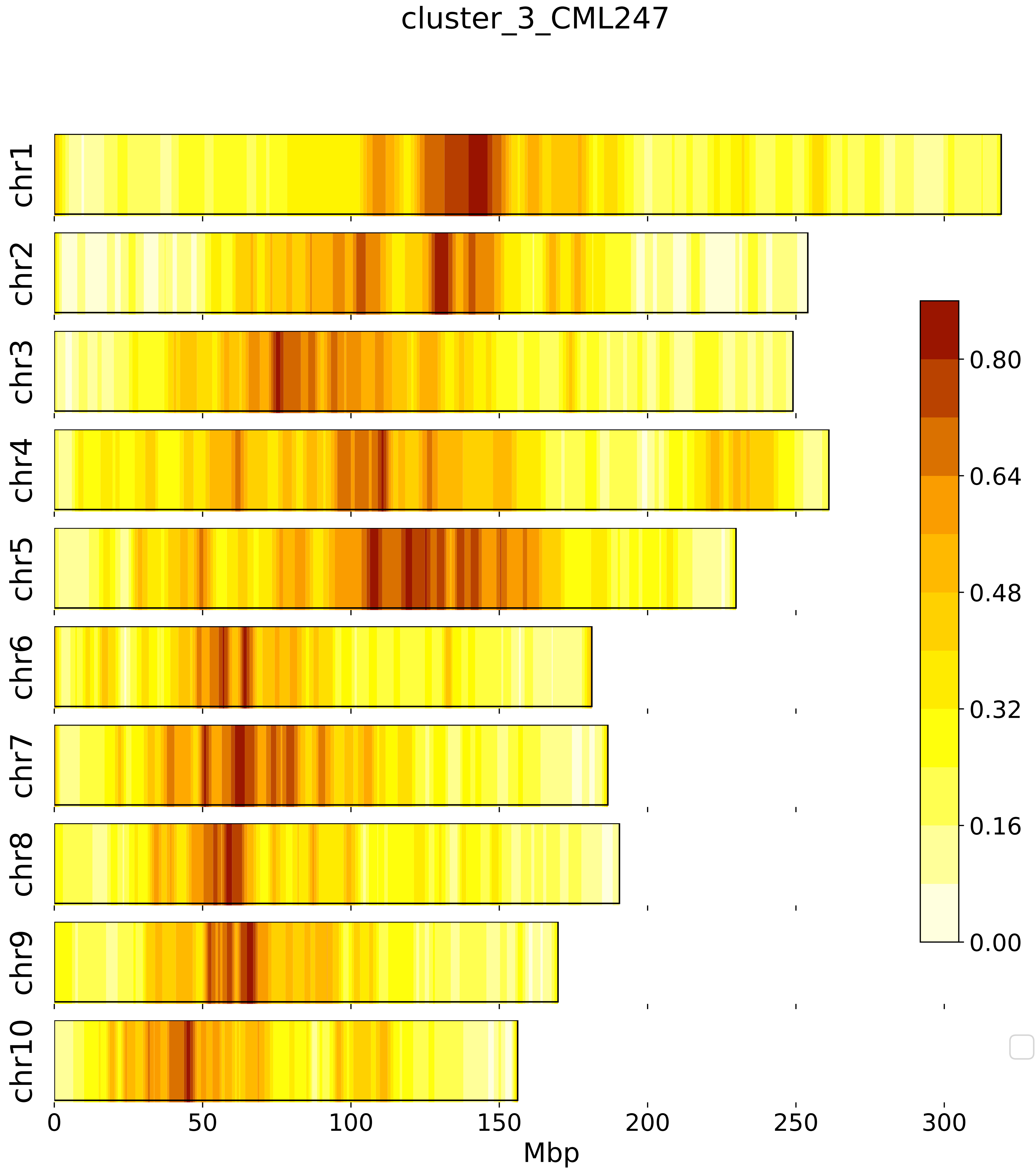
cluster_3_CML247
chr1
chr2
chr3
chr4
chr5
chr6
chr7
chr8
chr9
chr10
0
50
100
150
200
250
300
Mbp
0.00
0.16
0.32
0.48
0.64
0.80

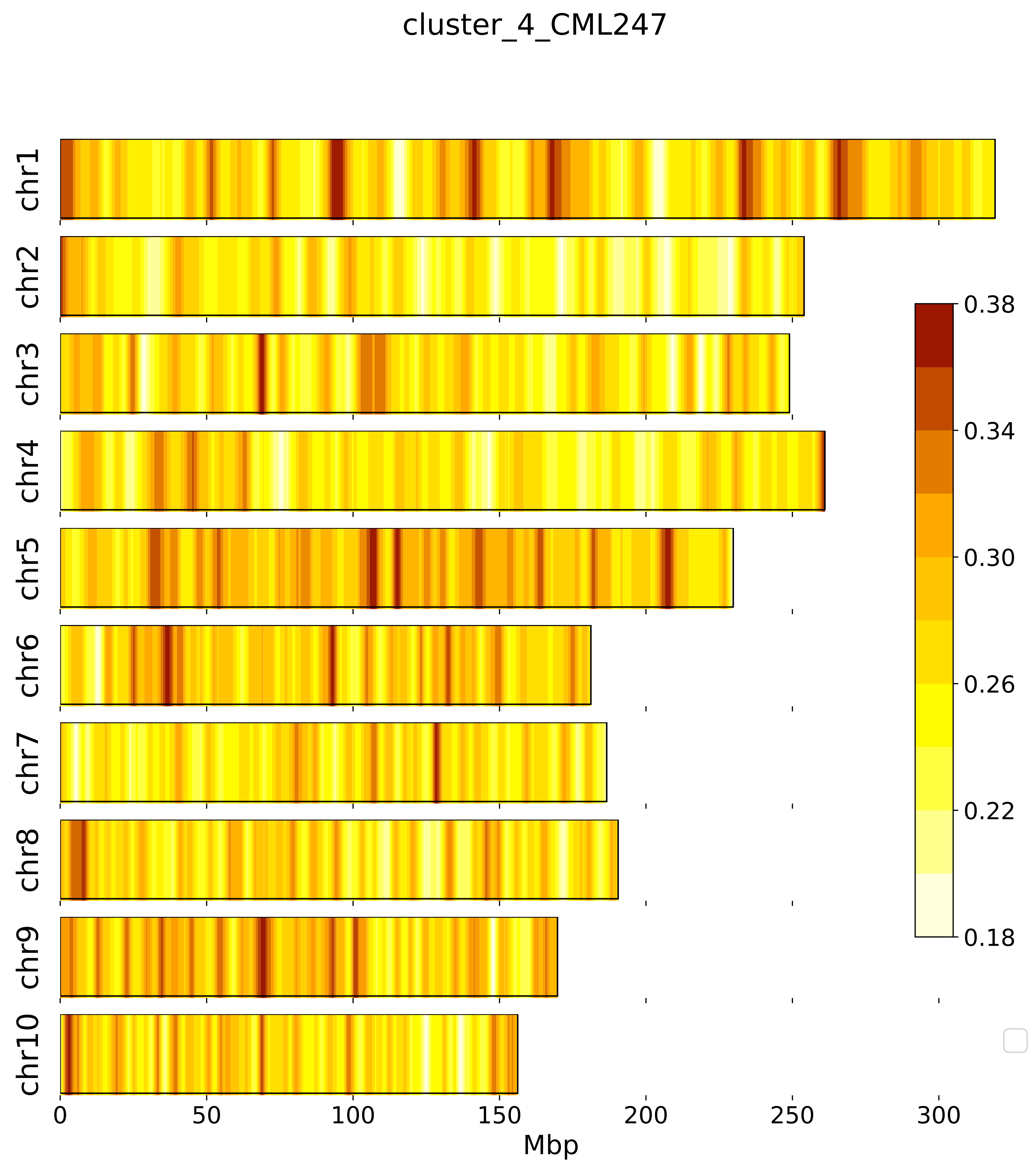

cluster_4_CML247
chr1
chr2
chr3
chr4
chr5
chr6
chr7
chr8
chr9
chr10
0
50
100
150
200
250
300
Mbp
0.38
0.34
0.30
0.26
0.22
0.18

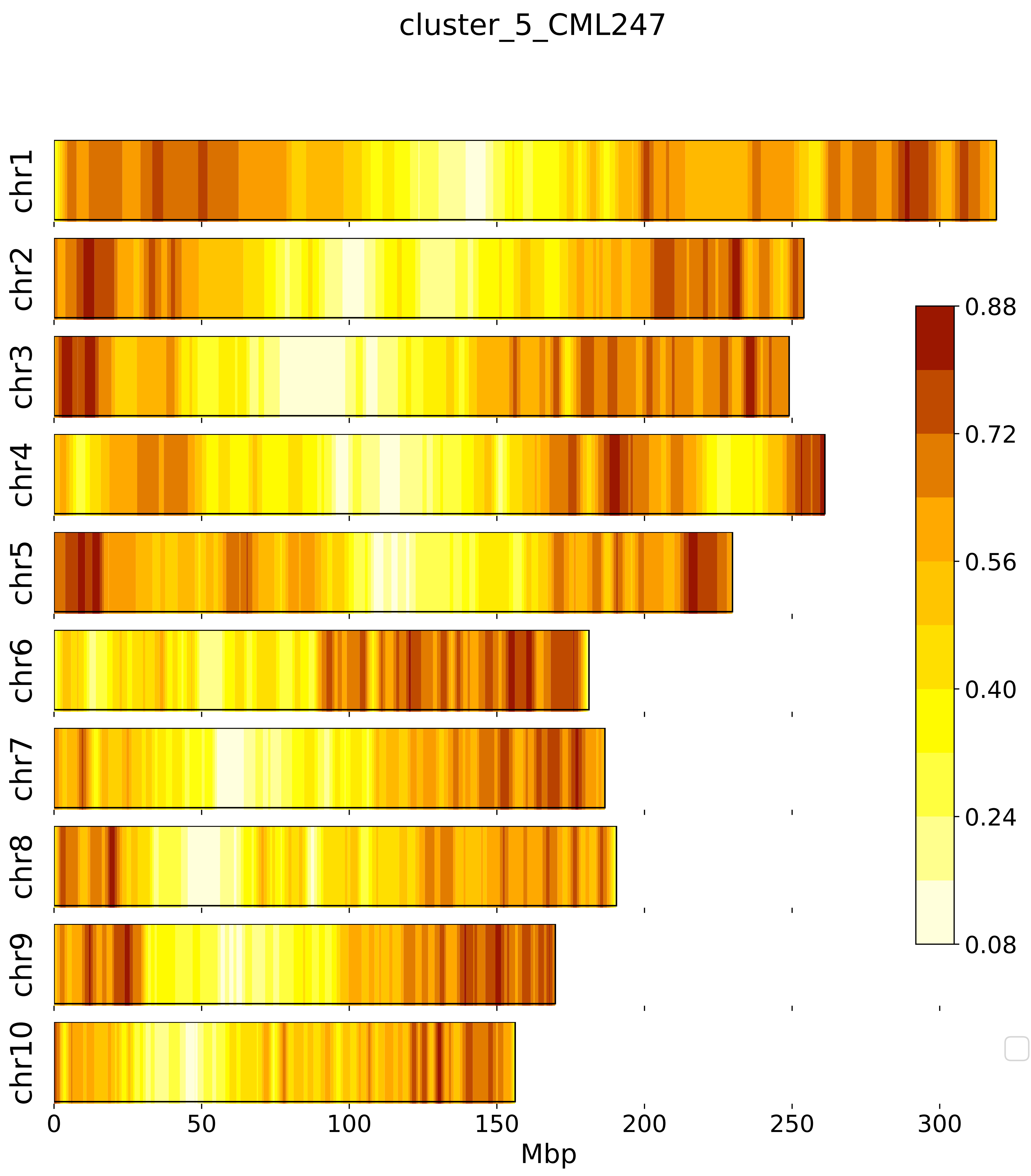

cluster_5_CML247
chr1
chr2
chr3
chr4
chr5
chr6
chr7
chr8
chr9
chr10
0
50
100
150
200
250
300
Mbp
0.88
0.72
0.56
0.40
0.24
0.08

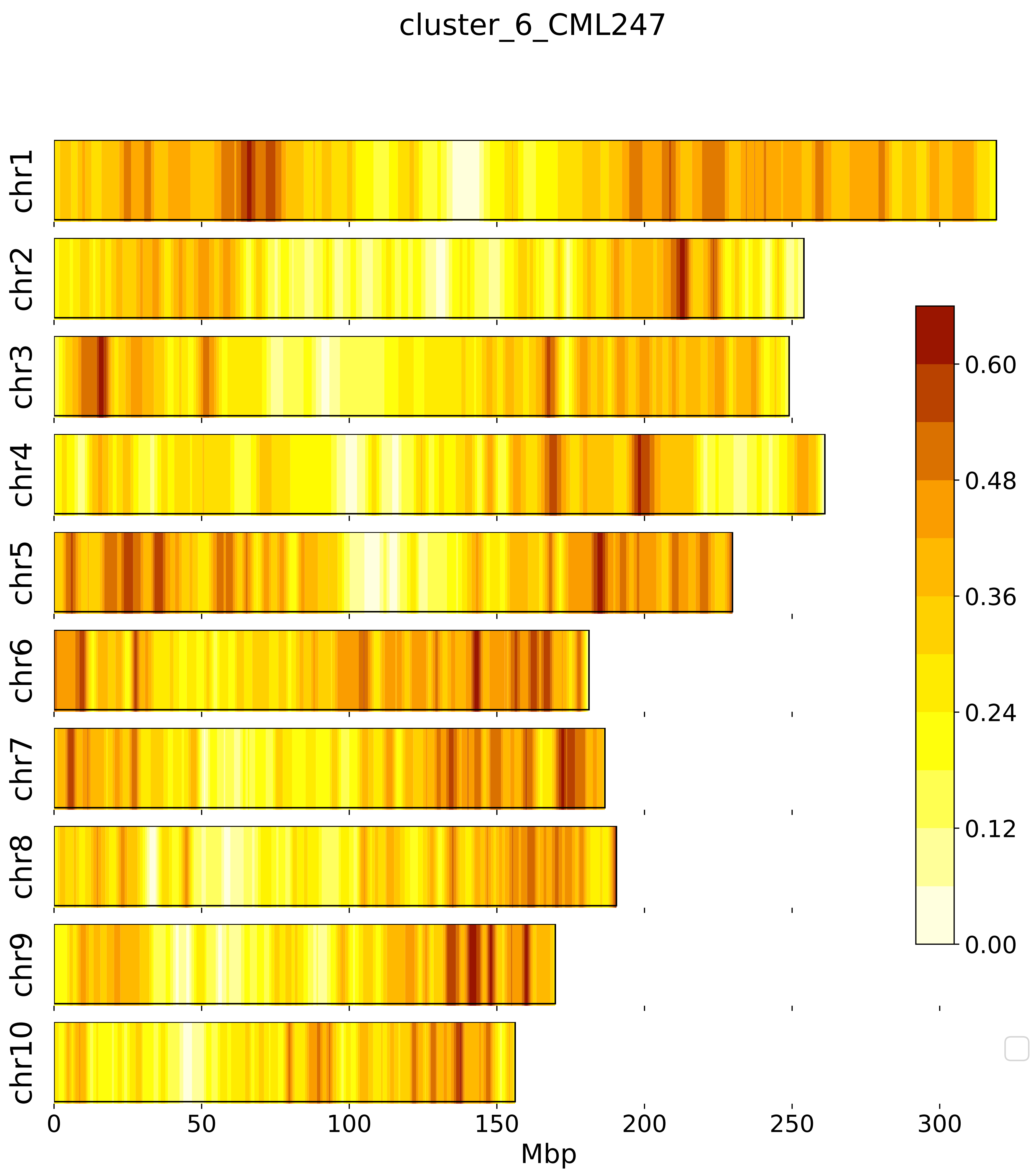

cluster_6_CML247
chr1
chr2
chr3
chr4
chr5
chr6
chr7
chr8
chr9
chr10
0
50
100
150
200
250
300
Mbp
0.00
0.12
0.24
0.36
0.48
0.60

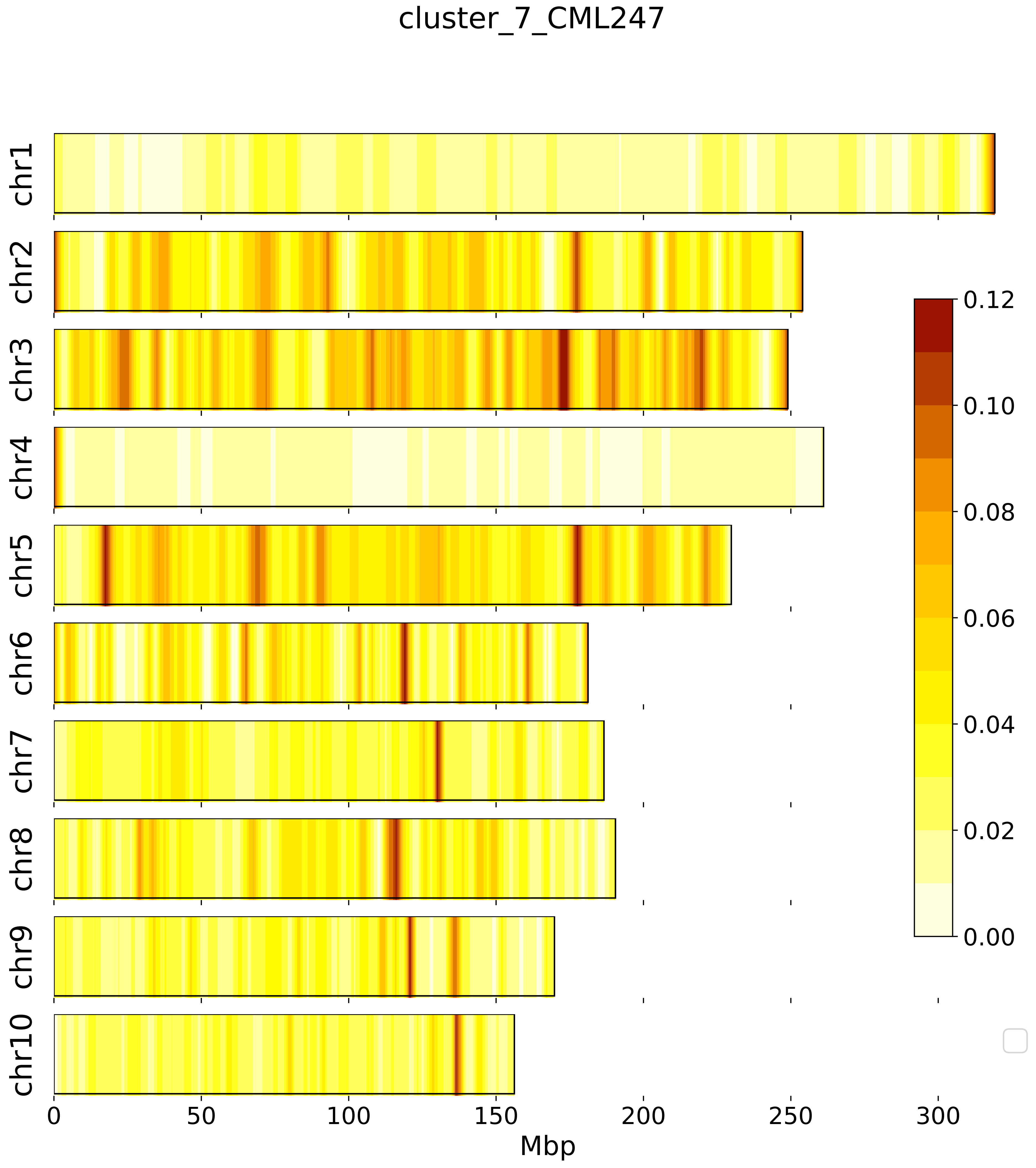
cluster_7_CML247
chr1
chr2
chr3
chr4
chr5
chr6
chr7
chr8
chr9
chr10
0
50
100
150
200
250
300
Mbp
0.12
0.10
0.08
0.06
0.04
0.02
0.00

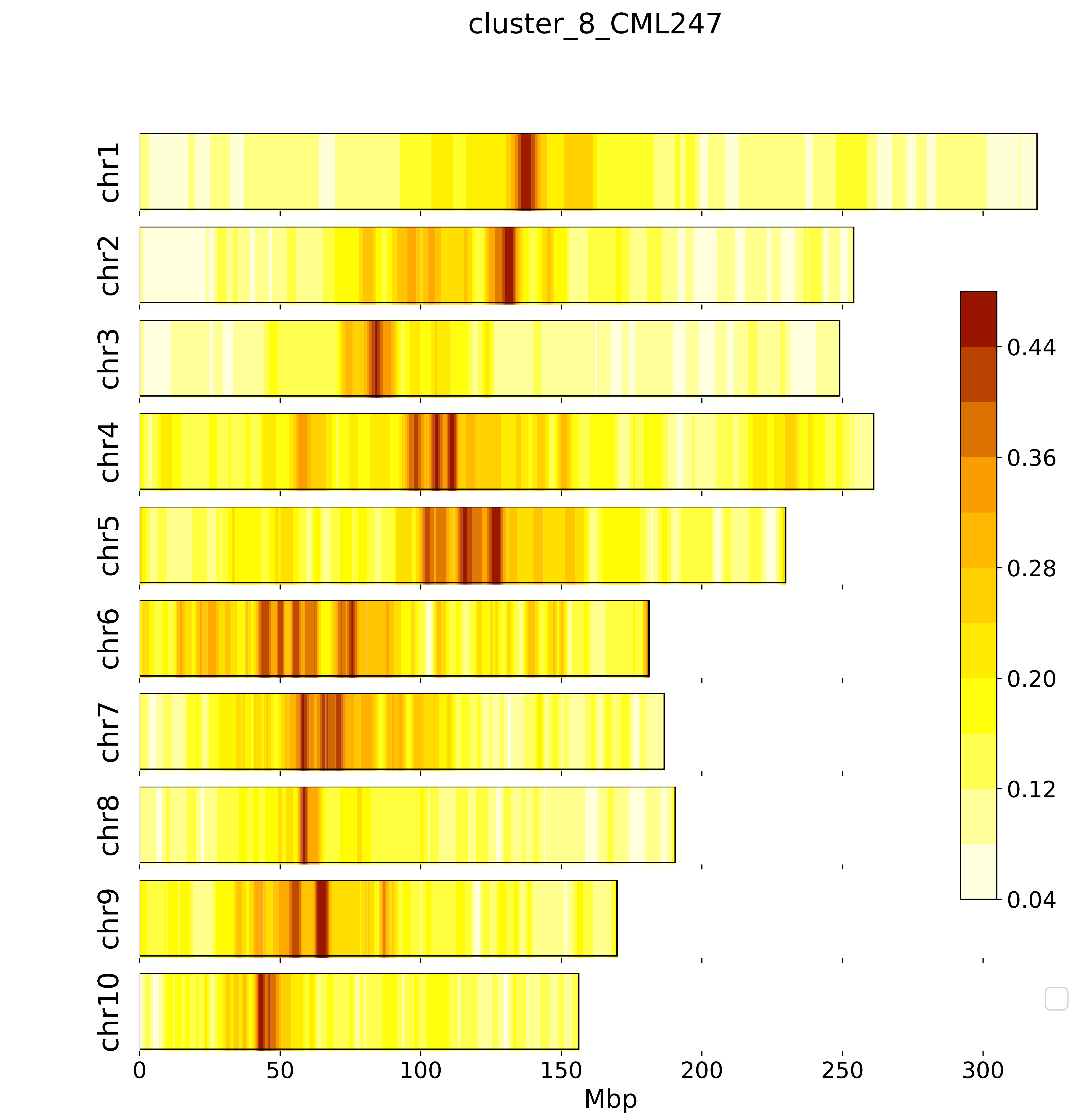
cluster_8_CML247
chr1
chr2
chr3
chr4
chr5
chr6
chr7
chr8
chr9
chr10
0
50
100
150
200
250
300
Mbp
0.44
0.36
0.28
0.20
0.12
0.04

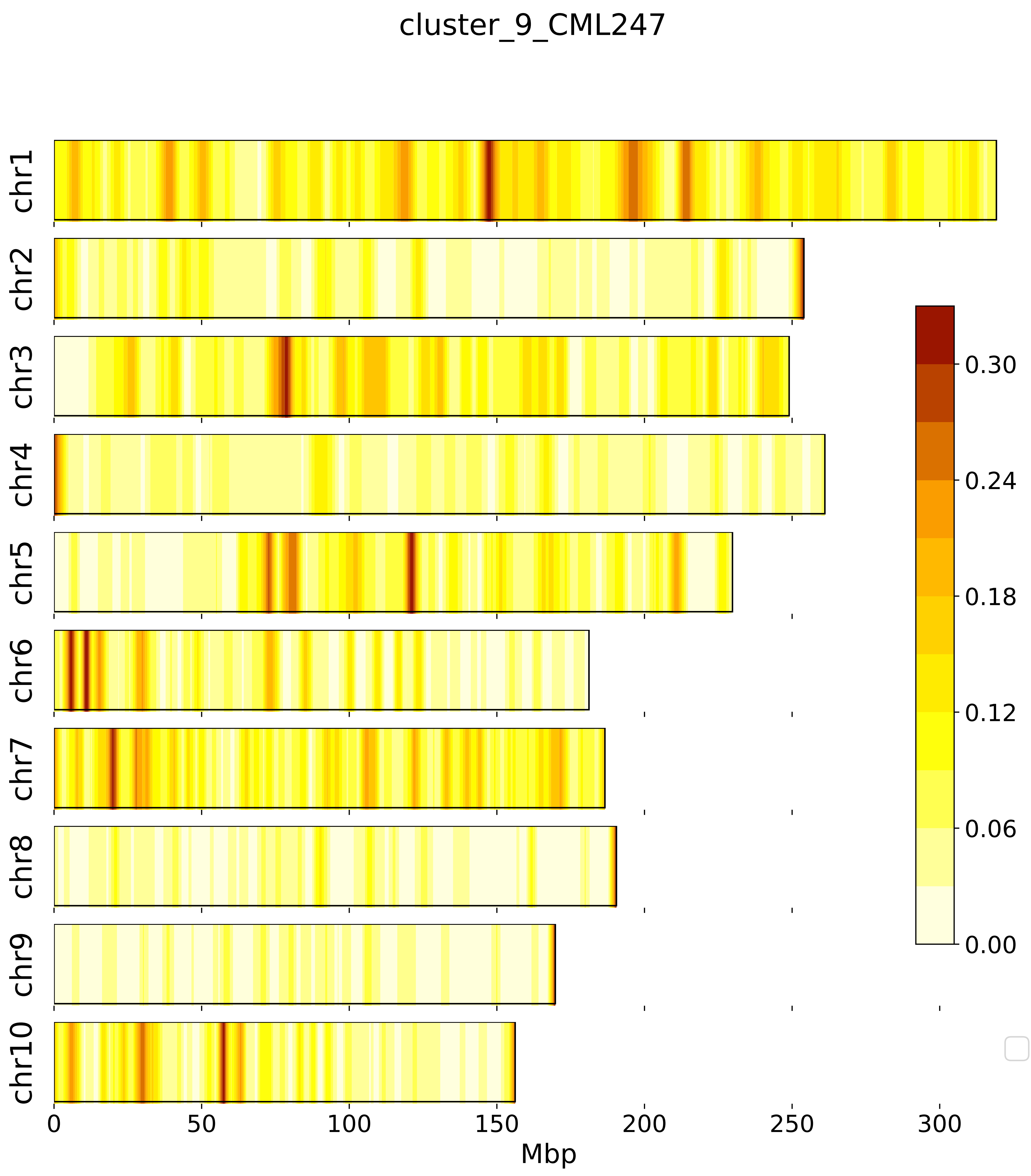

cluster_9_CML247
chr1
chr2
chr3
chr4
chr5
chr6
chr7
chr8
chr9
chr10
0
50
100
150
200
250
300
Mbp
0.30
0.24
0.18
0.12
0.06
0.00

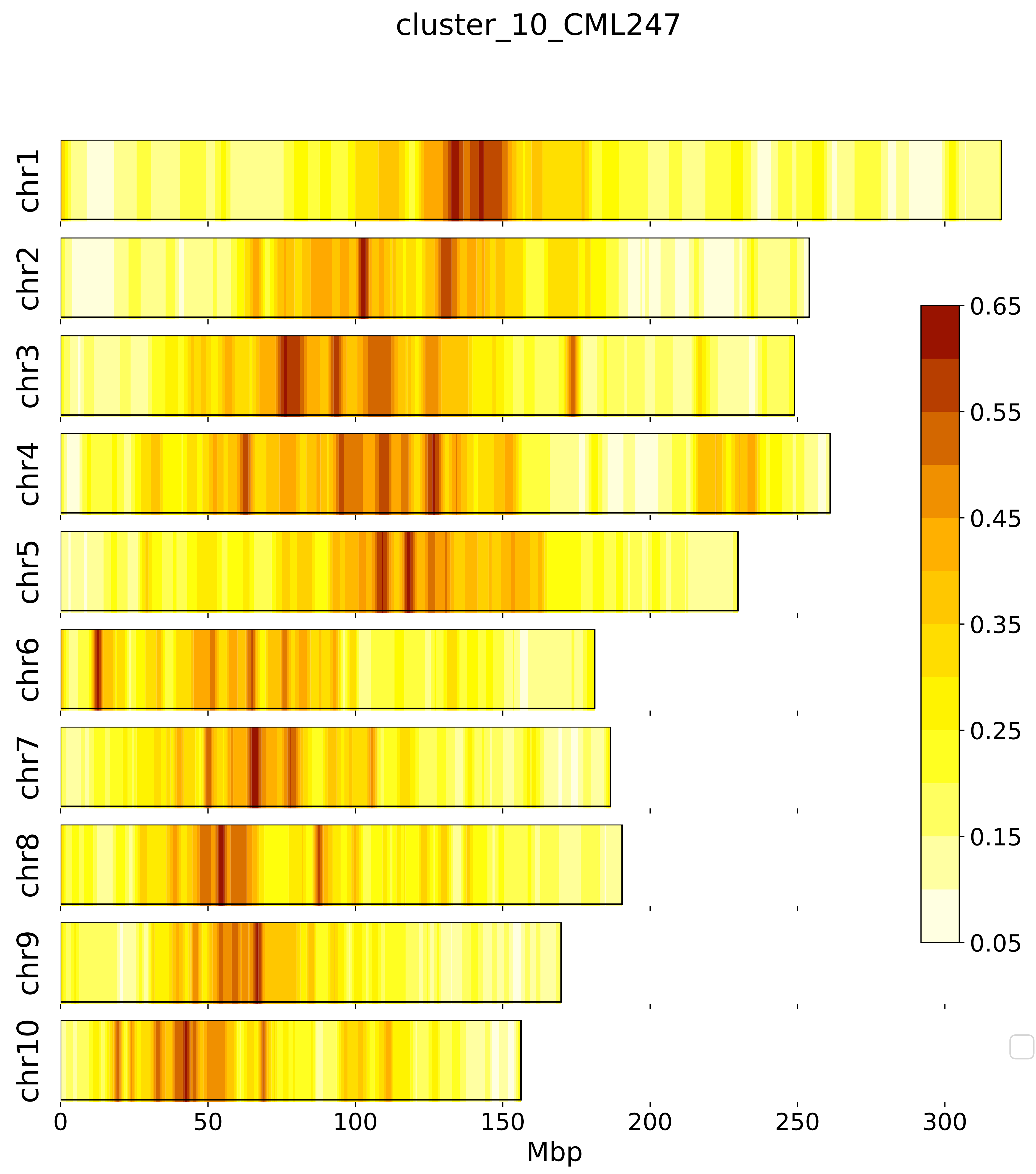

cluster_10_CML247
chr1
chr2
chr3
chr4
chr5
chr6
chr7
chr8
chr9
chr10
0
50
100
150
200
250
300
Mbp
0.65
0.55
0.45
0.35
0.25
0.15
0.05

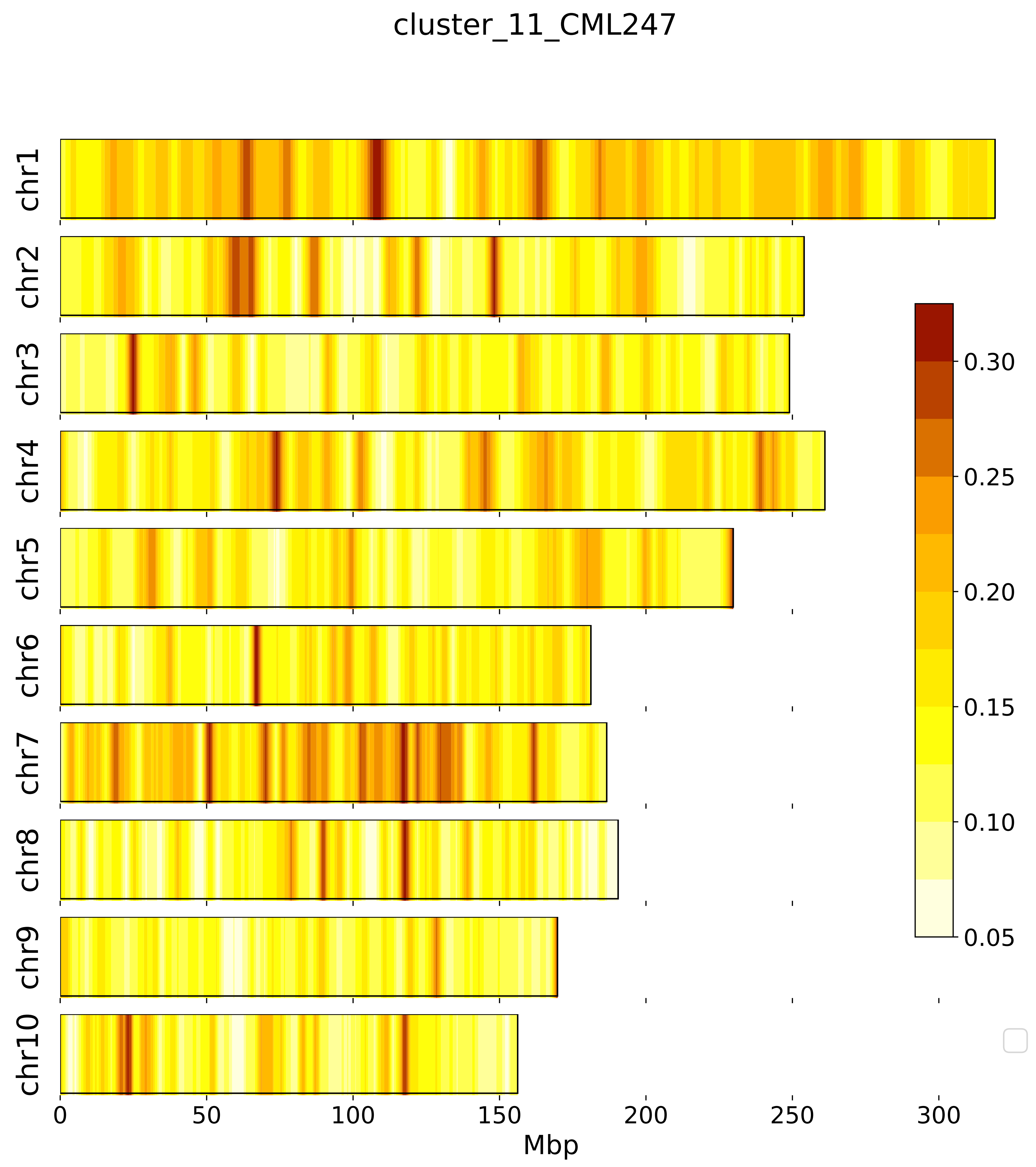
cluster_11_CML247
chr1
chr2
chr3
chr4
chr5
chr6
chr7
chr8
chr9
chr10
0
50
100
150
200
250
300
Mbp
0.30
0.25
0.20
0.15
0.10
0.05

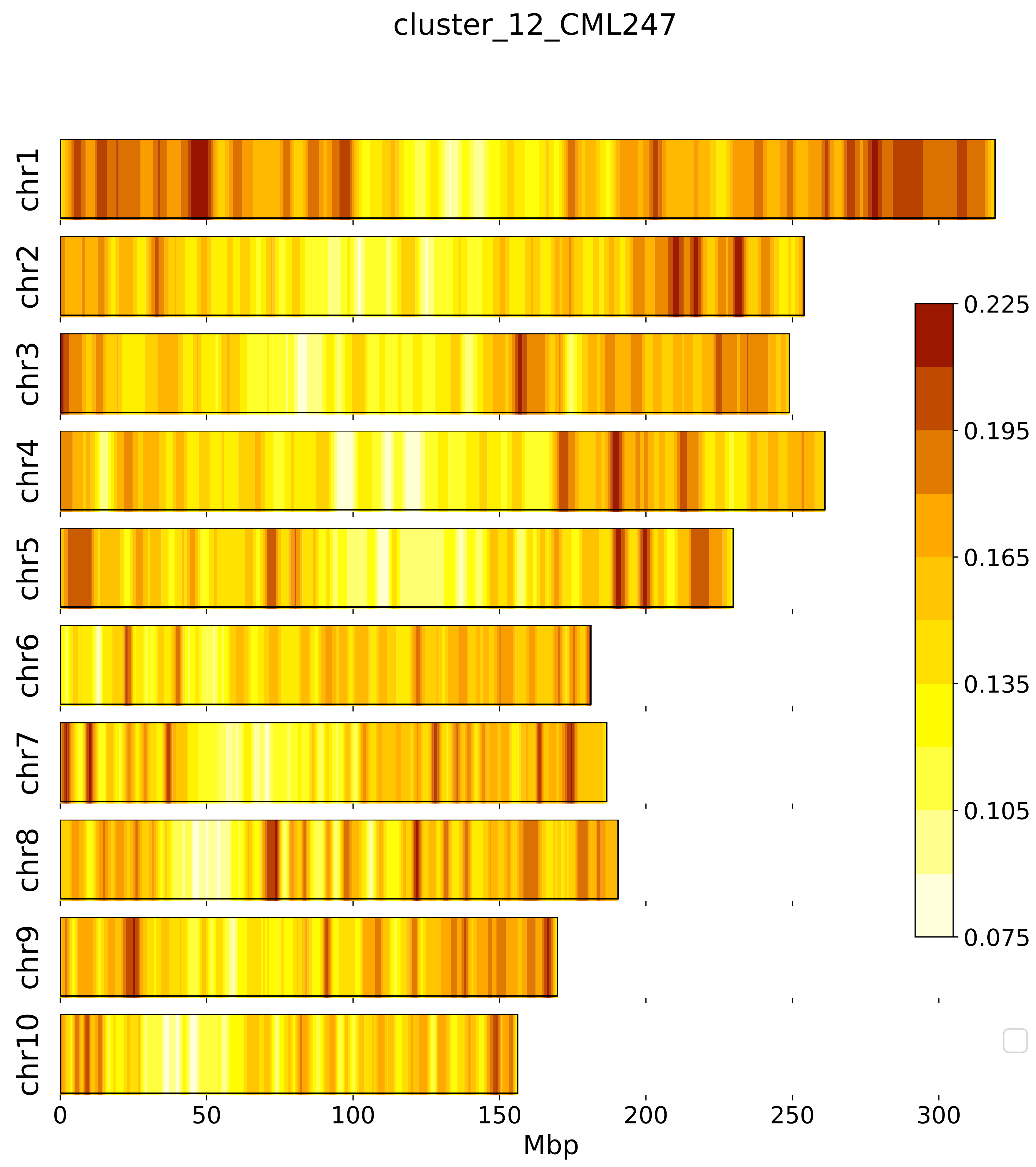

cluster_12_CML247
chr1
chr2
chr3
chr4
chr5
chr6
chr7
chr8
chr9
chr10
0
50
100
150
200
250
300
Mbp
0.225
0.195
0.165
0.135
0.105
0.075

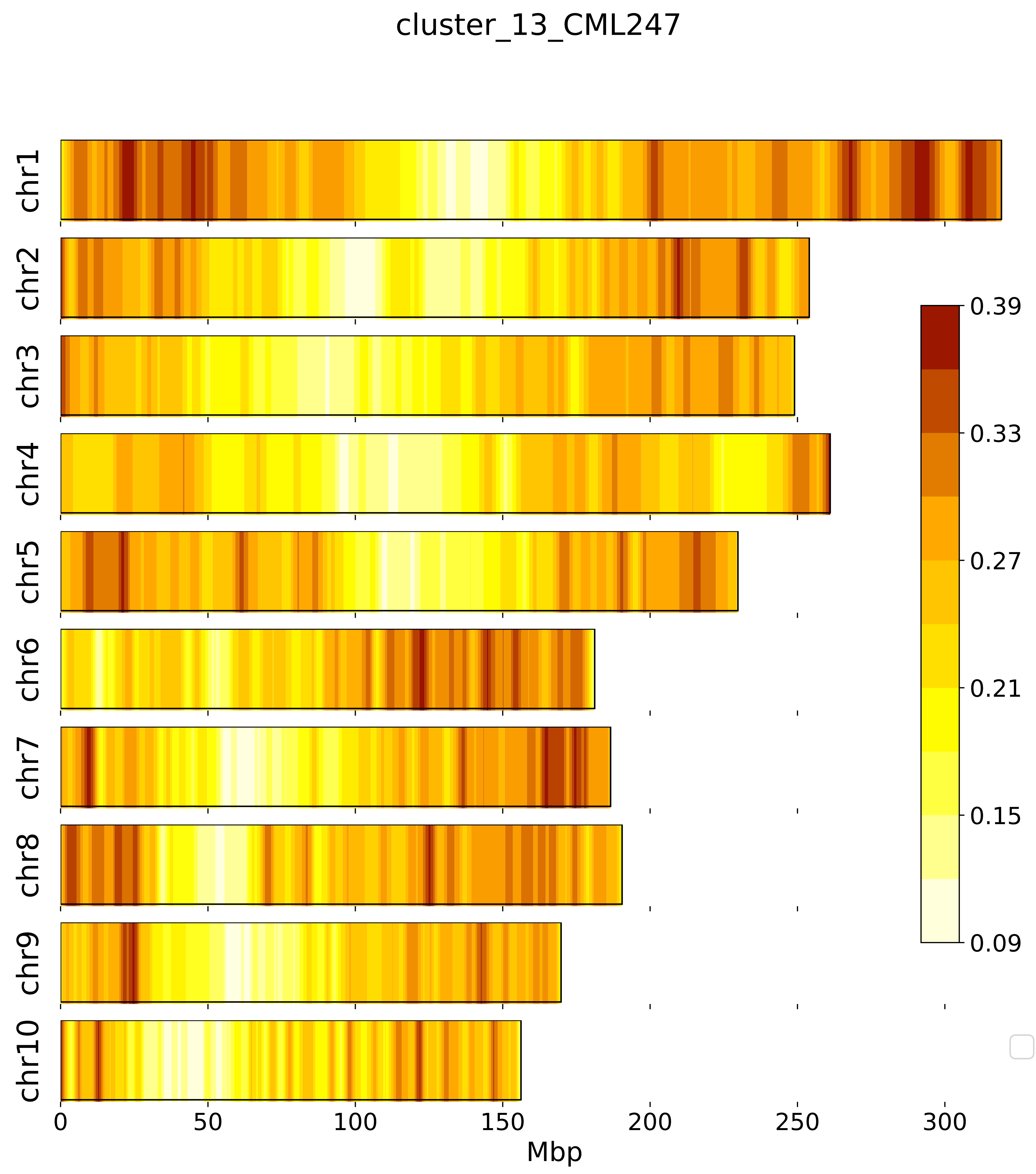
cluster_13_CML247
chr1
chr2
chr3
chr4
chr5
chr6
chr7
chr8
chr9
chr10
0
50
100
150
200
250
300
Mbp
0.39
0.33
0.27
0.21
0.15
0.09

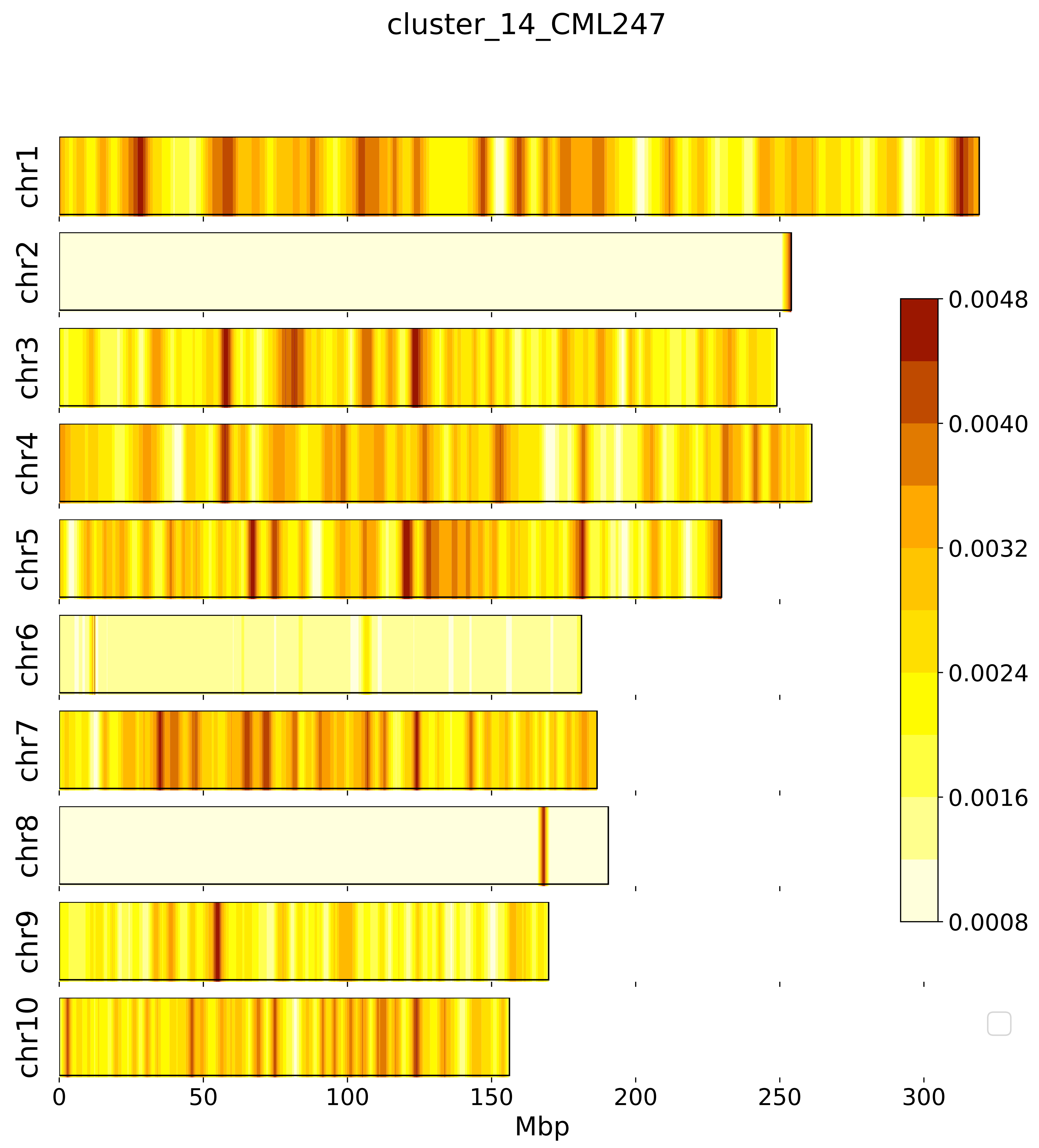

cluster_14_CML247
chr1
chr2
chr3
chr4
chr5
chr6
chr7
chr8
chr9
chr10
0
50
100
150
200
250
300
Mbp
0.0048
0.0040
0.0032
0.0024
0.0016
0.0008

cluster_15_CML247

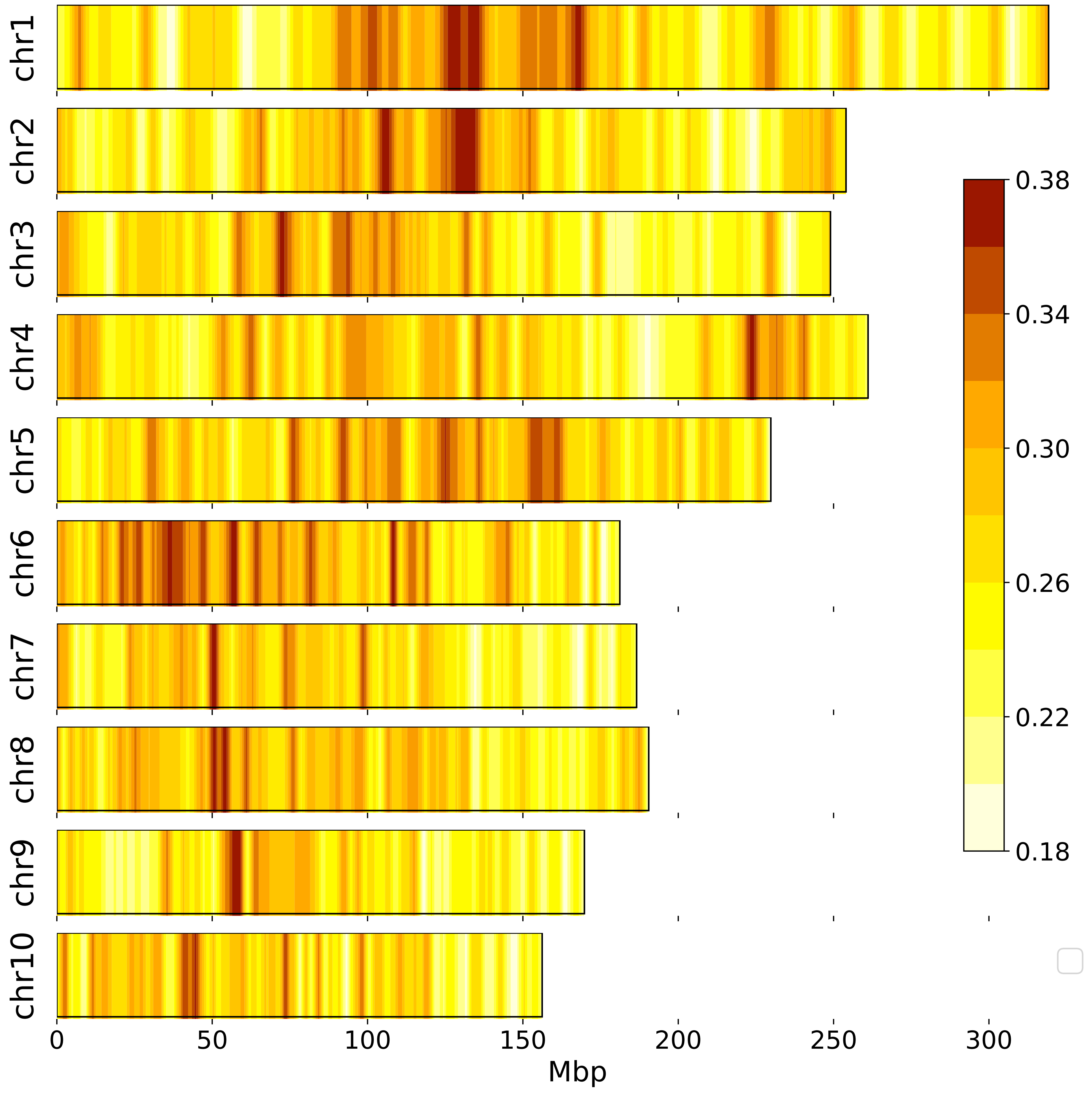

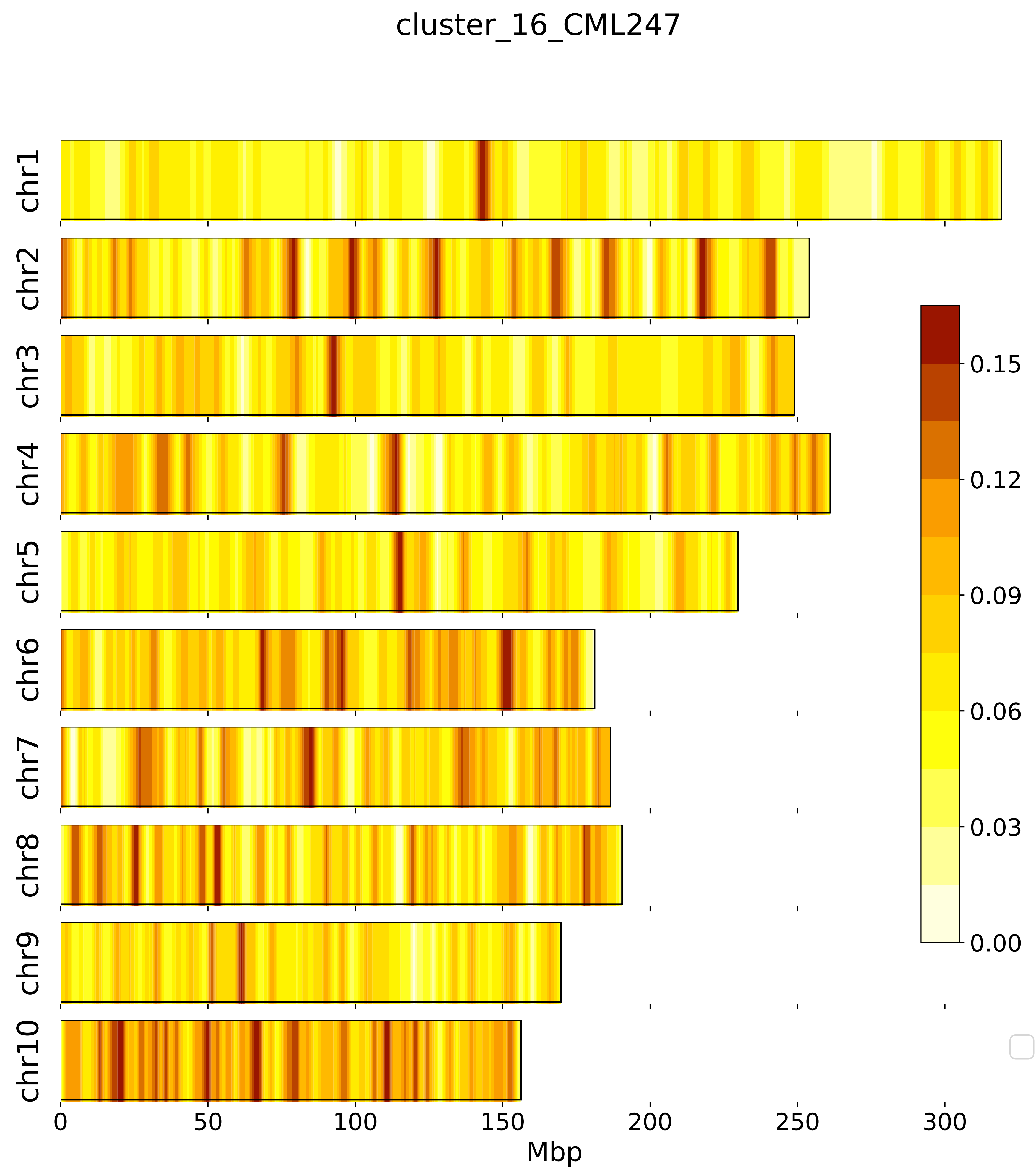

cluster_16_CML247
chr1
chr2
chr3
chr4
chr5
chr6
chr7
chr8
chr9
chr10
0
50
100
150
200
250
300
Mbp
0.15
0.12
0.09
0.06
0.03
0.00

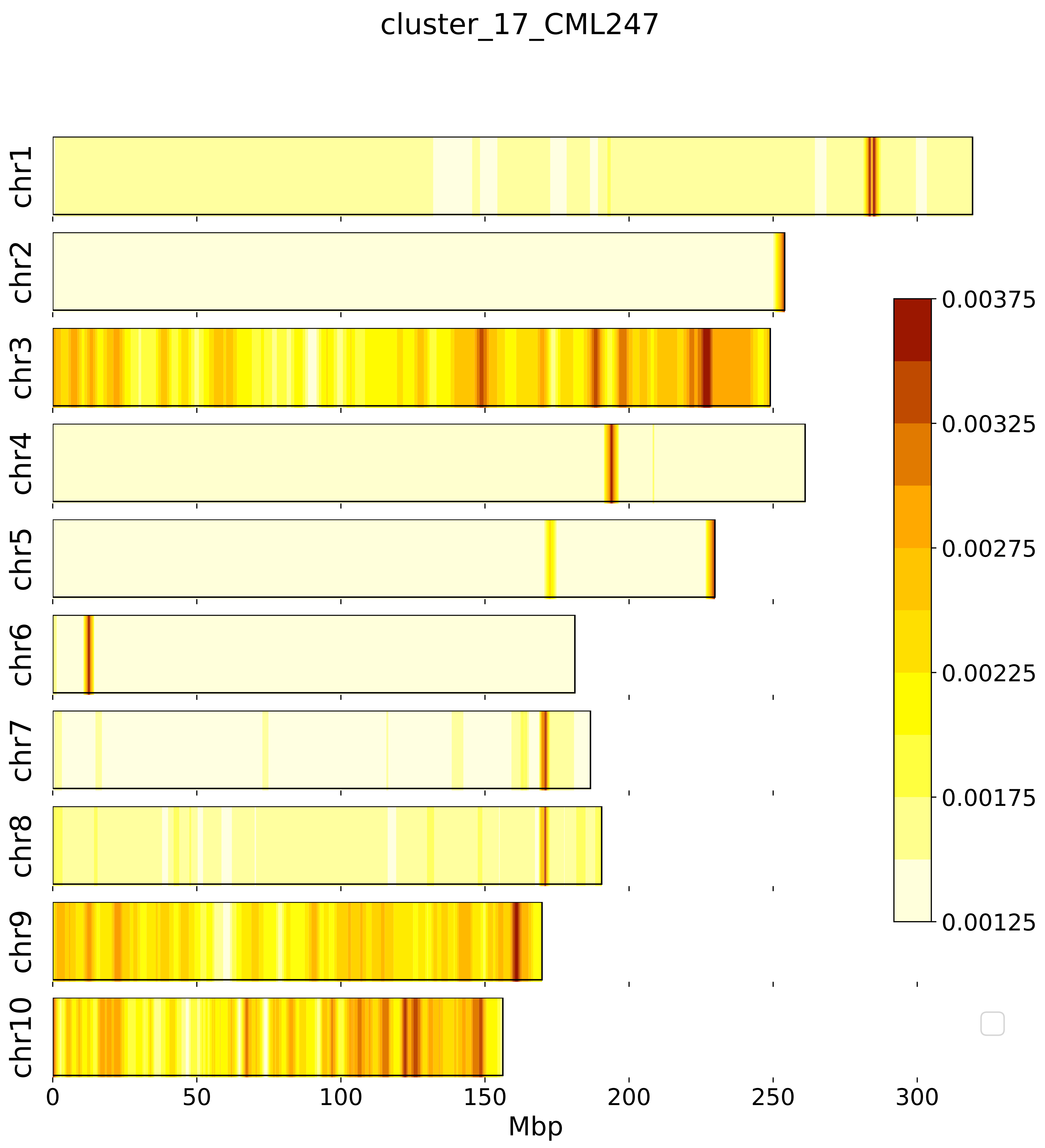
cluster_17_CML247
chr1
chr2
chr3
chr4
chr5
chr6
chr7
chr8
chr9
chr10
0
50
100
150
200
250
300
Mbp
0.00375
0.00325
0.00275
0.00225
0.00175
0.00125

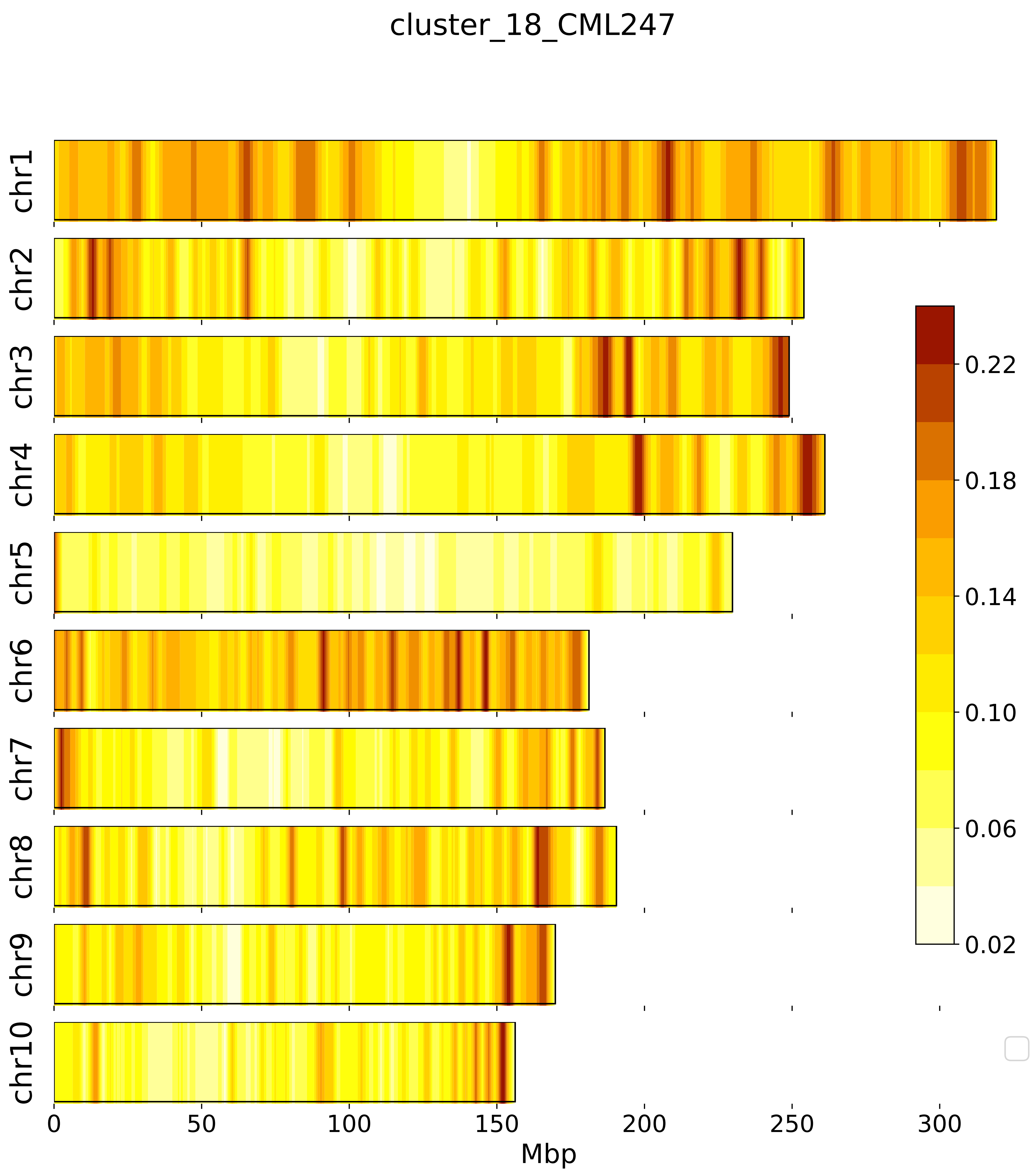

cluster_18_CML247
chr1
chr2
chr3
chr4
chr5
chr6
chr7
chr8
chr9
chr10
0
50
100
150
200
250
300
Mbp
0.22
0.18
0.14
0.10
0.06
0.02

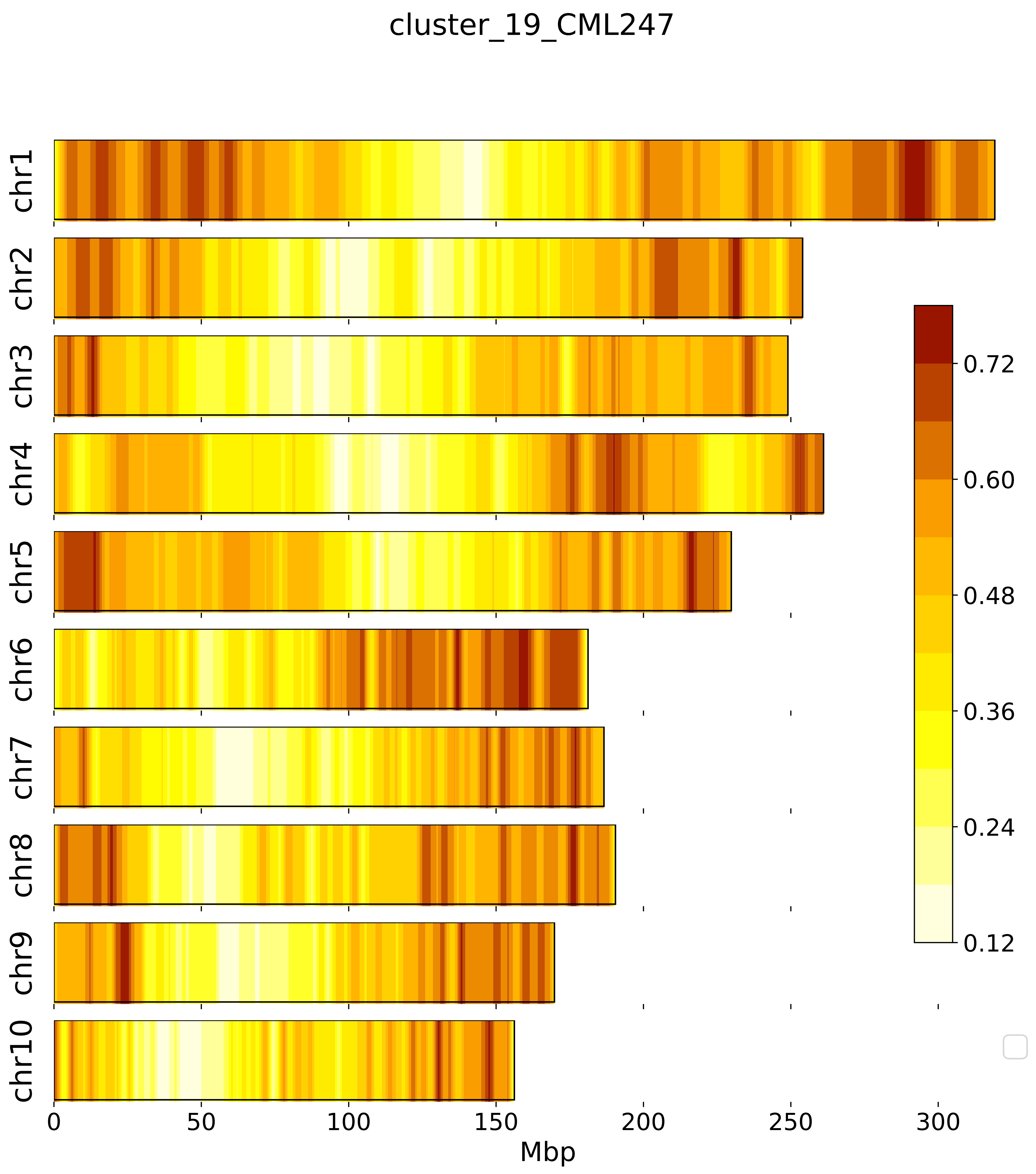

cluster_19_CML247
chr1
chr2
chr3
chr4
chr5
chr6
chr7
chr8
chr9
chr10
0
50
100
150
200
250
300
Mbp
0.72
0.60
0.48
0.36
0.24
0.12

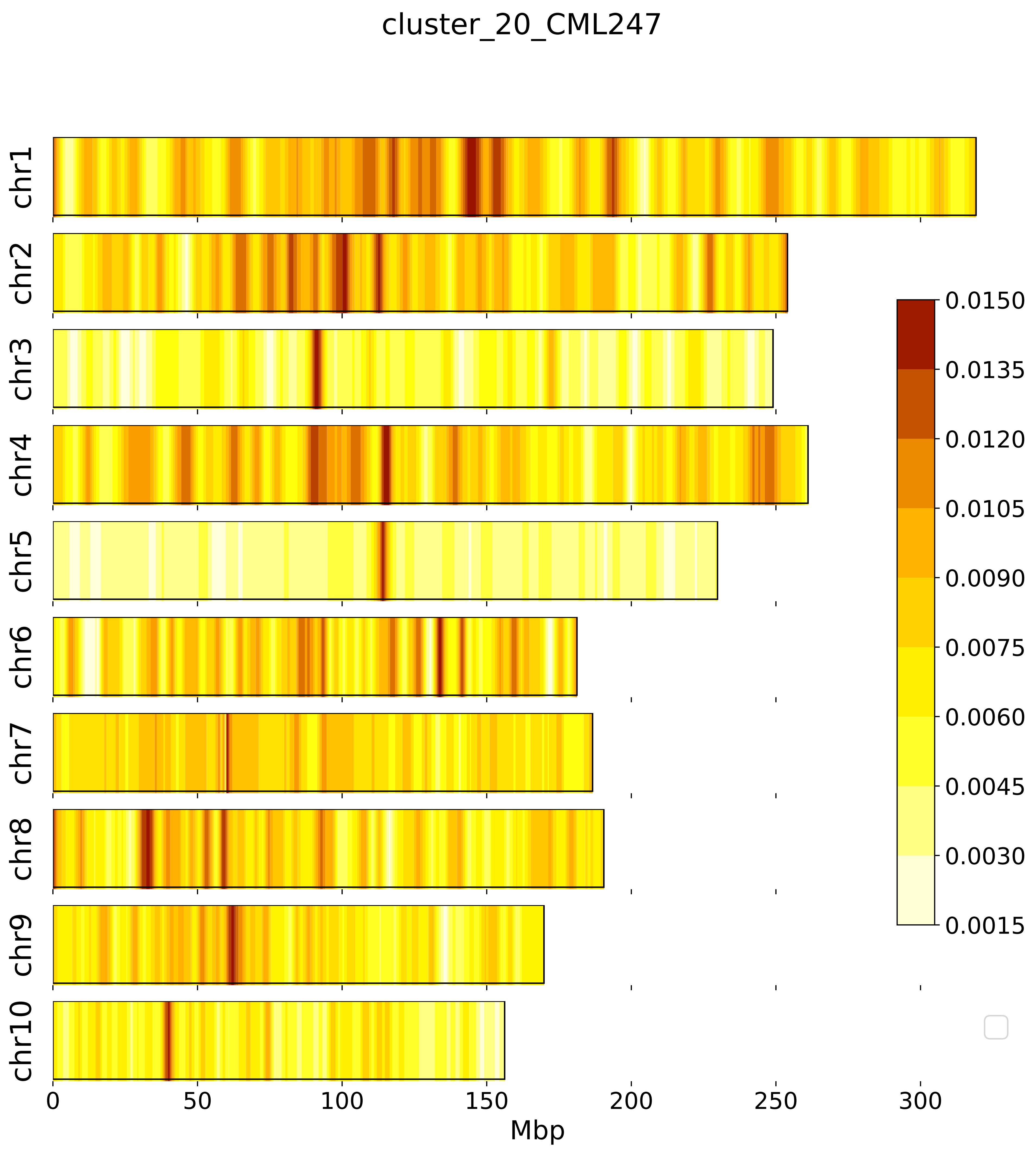

cluster_20_CML247
chr1
chr2
chr3
chr4
chr5
chr6
chr7
chr8
chr9
chr10
0
50
100
150
200
250
300
Mbp
0.0150
0.0135
0.0120
0.0105
0.0090
0.0075
0.0060
0.0045
0.0030
0.0015

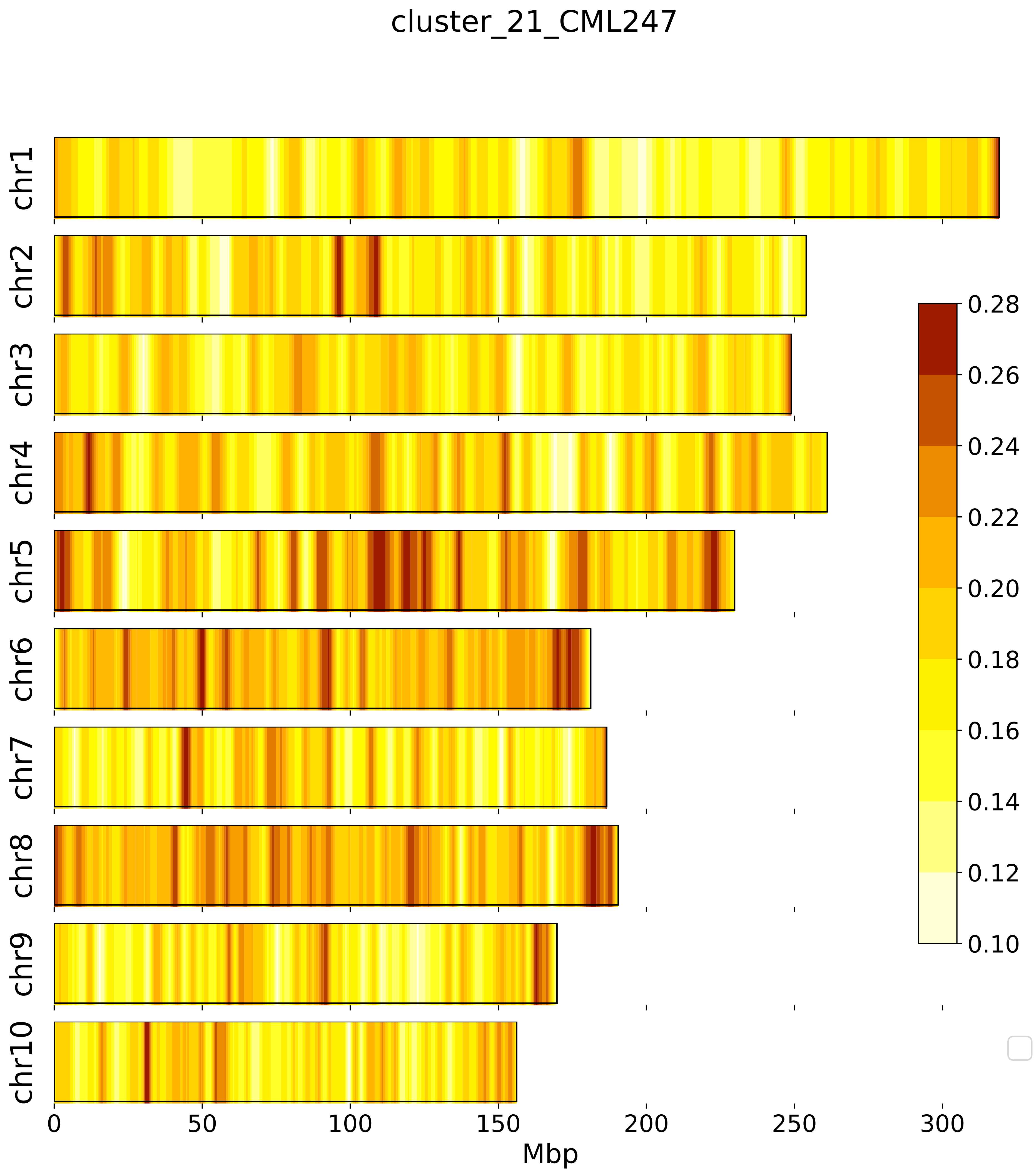
cluster_21_CML247
chr1
chr2
chr3
chr4
chr5
chr6
chr7
chr8
chr9
chr10
0
50
100
150
200
250
300
Mbp
0.28
0.26
0.24
0.22
0.20
0.18
0.16
0.14
0.12
0.10

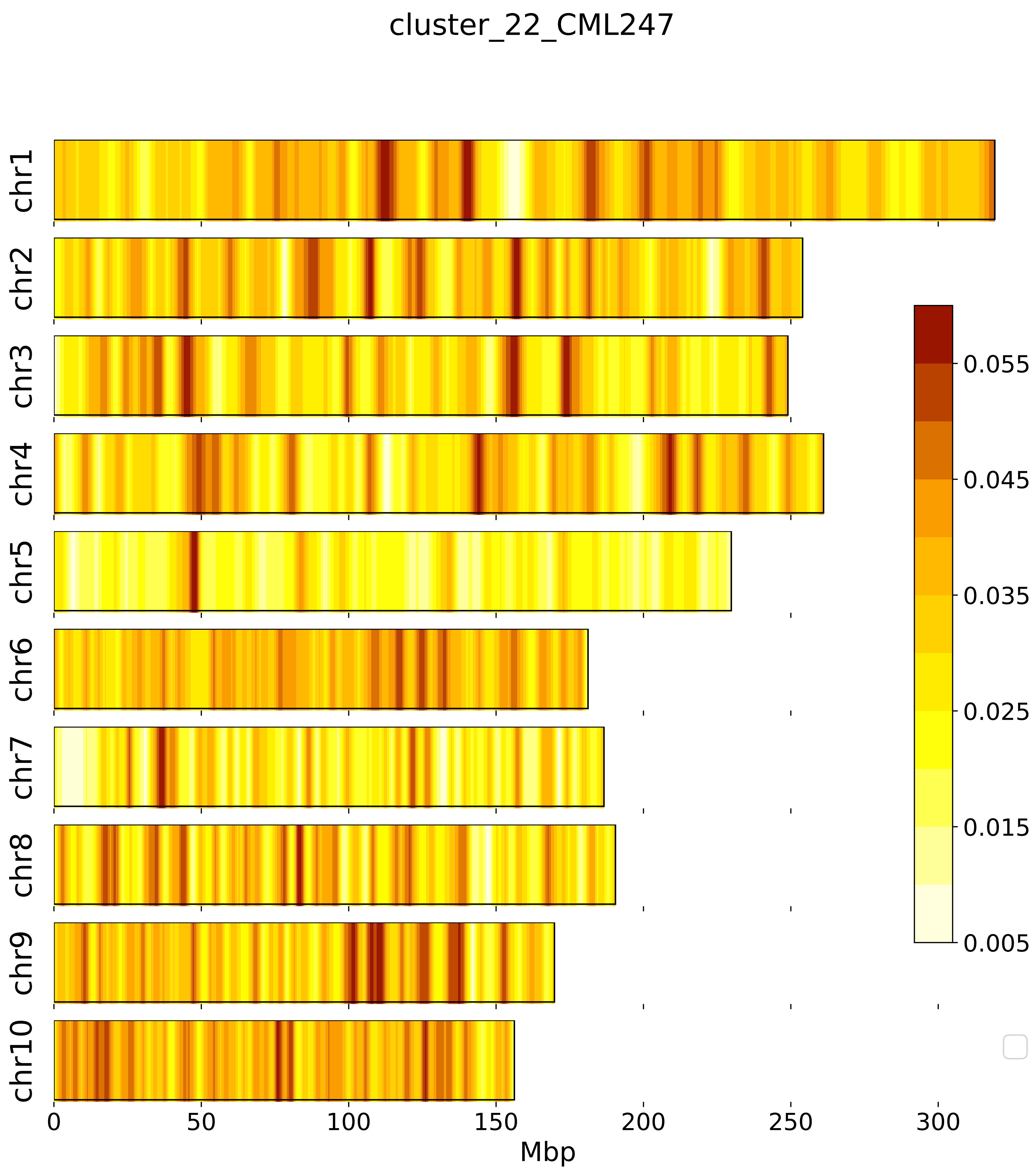
cluster_22_CML247
chr1
chr2
chr3
chr4
chr5
chr6
chr7
chr8
chr9
chr10
0
50
100
150
200
250
300
Mbp
0.055
0.045
0.035
0.025
0.015
0.005

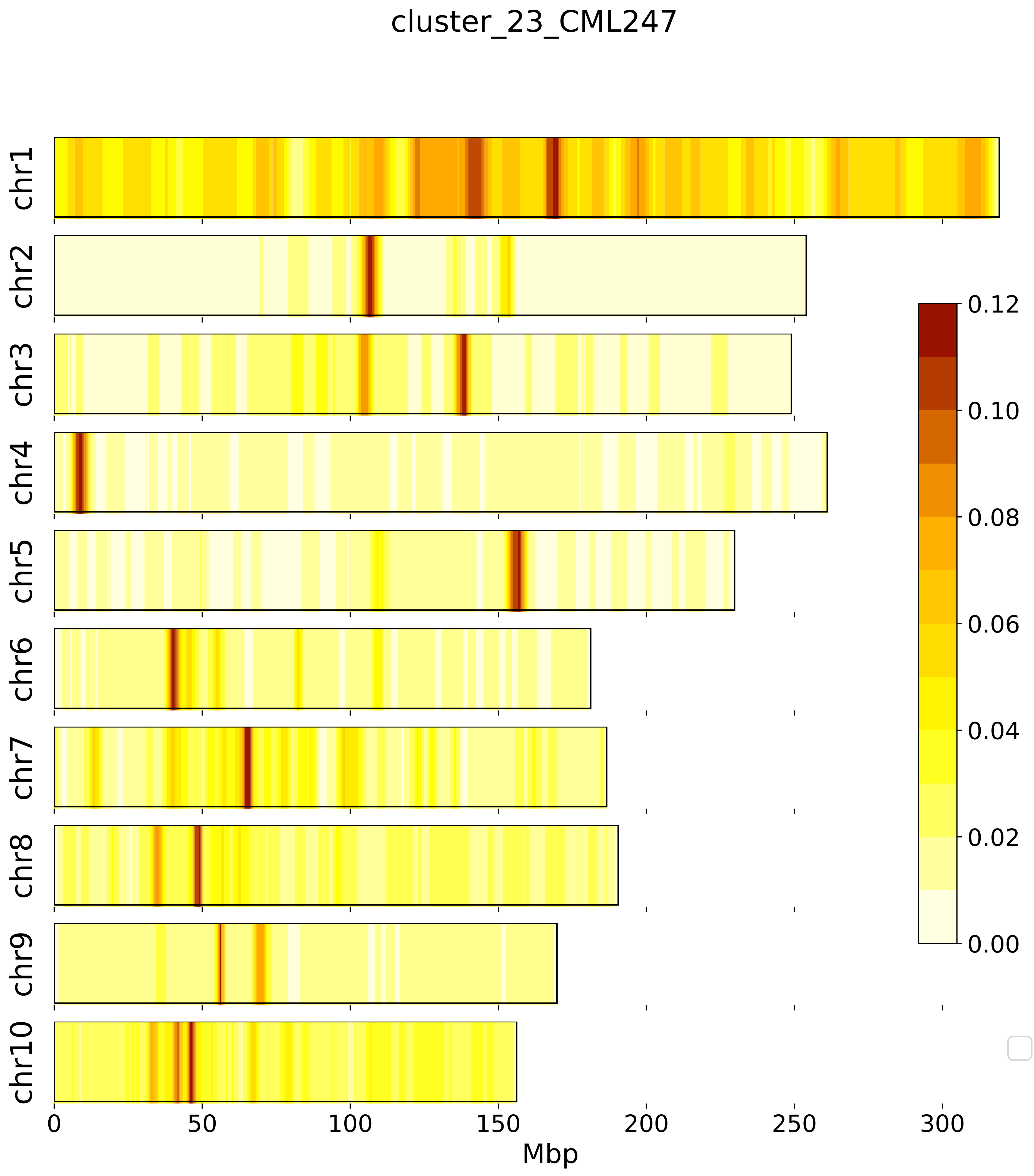
cluster_23_CML247
chr1
chr2
chr3
chr4
chr5
chr6
chr7
chr8
chr9
chr10
0
50
100
150
200
250
300
Mbp
0.00
0.02
0.04
0.06
0.08
0.10
0.12

cluster_24_CML247

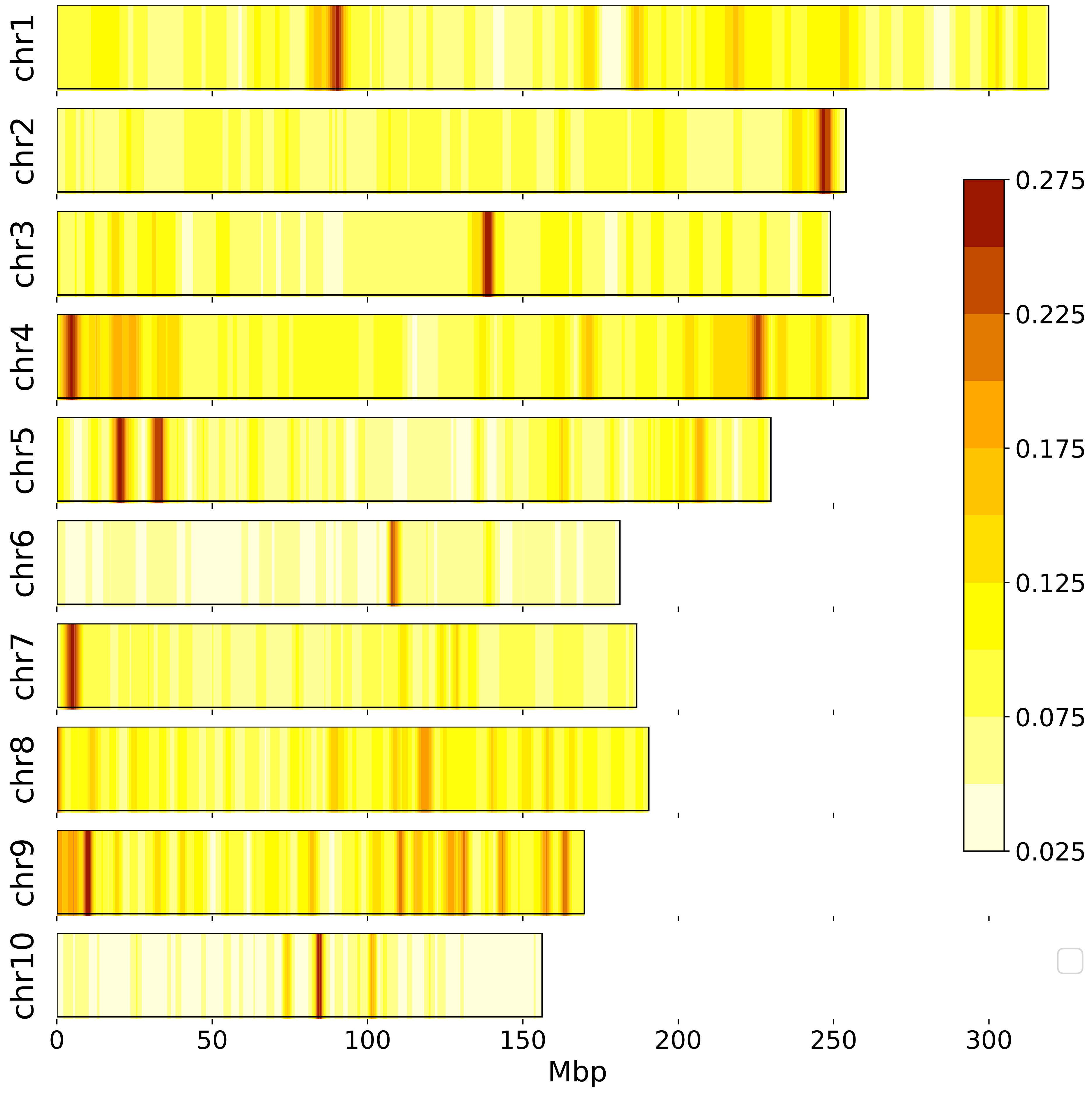

Supplement: Supplementary file 8 — Additional file 8 Genome-wide kmer distribution in CML247. The genome-wide K-mer distribution for each of the 25 largest repeat clusters. A Kernel density estimation is indicated by color, and associated gene density estimates are also given. [file 12864_2020_6517_MOESM8_ESM.pdf]
